# CMAUP: a database of collective molecular activities of useful plants

**DOI:** 10.1093/nar/gky965

**Published:** 2018-10-24

**Authors:** Xian Zeng, Peng Zhang, Yali Wang, Chu Qin, Shangying Chen, Weidong He, Lin Tao, Ying Tan, Dan Gao, Bohua Wang, Zhe Chen, Weiping Chen, Yu Yang Jiang, Yu Zong Chen

**Affiliations:** 1 The State Key Laboratory of Chemical Oncogenomics, Key Laboratory of Chemical Biology, Tsinghua University Shenzhen Graduate School, Shenzhen Technology and Engineering Laboratory for Personalized Cancer Diagnostics and Therapeutics, Shenzhen Kivita Innovative Drug Discovery Institute, Guangdong 518055, P. R. China; 2 Bioinformatics and Drug Design group, Department of Pharmacy, National University of Singapore, Singapore 117543, Singapore; 3 Zhejiang Key Laboratory of Gastro-intestinal Pathophysiology, Zhejiang Hospital of Traditional Chinese Medicine, Zhejiang Chinese Medical University, School of Medicine, Hangzhou Normal University, Hangzhou 310006, R. P. China; 4 Key Lab of Agricultural Products Processing and Quality Control of Nanchang City, Jiangxi Agricultural University, Nanchang 330045, P. R. China; 5 College of Life and Environmental Sciences, Collaborative Innovation Center for Efficient and Health Production of Fisheries in Hunan Province, Hunan University of Arts and Science, Changde, Hunan 415000, P. R. China

## Abstract

The beneficial effects of functionally useful plants (e.g. medicinal and food plants) arise from the multi-target activities of multiple ingredients of these plants. The knowledge of the collective molecular activities of these plants facilitates mechanistic studies and expanded applications. A number of databases provide information about the effects and targets of various plants and ingredients. More comprehensive information is needed for broader classes of plants and for the landscapes of individual plant’s multiple targets, collective activities and regulated biological pathways, processes and diseases. We therefore developed a new database, Collective Molecular Activities of Useful Plants (CMAUP), to provide the collective landscapes of multiple targets (ChEMBL target classes) and activity levels (in 2D target-ingredient heatmap), and regulated gene ontologies (GO categories), biological pathways (KEGG categories) and diseases (ICD blocks) for 5645 plants (2567 medicinal, 170 food, 1567 edible, 3 agricultural and 119 garden plants) collected from or traditionally used in 153 countries and regions. These landscapes were derived from 47 645 plant ingredients active against 646 targets in 234 KEGG pathways associated with 2473 gene ontologies and 656 diseases. CMAUP (http://bidd2.nus.edu.sg/CMAUP/) is freely accessible and searchable by keywords, plant usage classes, species families, targets, KEGG pathways, gene ontologies, diseases (ICD code) and geographical locations.

## INTRODUCTION

Many functionally useful plants have been extensively investigated and explored for benefiting humans. Herb-based indigenous medicines and medicinal plants have been and are continuously being used as therapeutics by huge population in the world (1–4). Food and edible plants have been studied for increased food productivity or nutrition yield under different environmental conditions (5,6). Their health-beneficiary (7,8) and toxic (9) effects have been studied for appropriate applications and safe uses. Expanding number of novel garden plants have been discovered or engineered by such methods as genetic manipulations (10).

The broader explorations of the functionally useful plants have been facilitated by the molecular and systems biology studies of their functional mechanisms. For instance, the mechanisms of the synergistic combinations of multiple herbal ingredients (11) and the traditionally used synergistic herb-pairs (12) in traditional Chinese medicine (TCM) have been revealed by the analysis of their molecular interaction profiles and chemical structural similarity landscapes from the perspectives of systems biology. The regulatory roles of the metabolic pathways and transporter genes of food plants have been investigated and manipulated for increased food productivity particularly in the hotter and drier environment (5) and under soil salinity conditions (6). The functions and the expression of the selected combinations of genes have been studied for deriving salt-resistant crops (6). Exotic garden plants have been produced by the alteration of the gene expression levels in specific biosynthetic pathways (10).

Such investigations and developmental efforts can be facilitated by the availability of the information about individual plant’s collective activities and multiple targets of its ingredients, and the regulated biological pathways, biological processes and diseases for more diverse classes of medicinal, food, edible, garden, and other functionally useful plants. Established plant databases (e.g. Plants of the World Online (http://www.plantsoftheworldonline.org), The Plant List (http://www.theplantlist.org), the PLANTS database (https://plants.sc.egov.usda.gov) and the Plant Trait Database (https://www.try-db.org/TryWeb/Home.php)) provide comprehensive information about the characteristics, taxonomy and resources of large number plants, but the plant ingredient and activity information are unincluded. Specialized databases provide additional information about medicinal plants (e.g. KANpSAcK (13), IMPPAT (14), BATMA-TCM (15), NuBBE (16), TM-MC (17), TCM@Taiwan (18), TCMID (19), TIPdb-3D (20), TCMSP (21), TCM-ID (22) and TCMAnalyzer (23), food and edible plants (e.g. KANpSAcK (13) and Nutrichem (24), ePlantLIBRA (25), and the species that produce bioactive compounds (e.g. NPASS (26) and HIT (27).

Some of these databases provide the information about the targets or activities of plant ingredients. In particular, TCMSP (21) and TCMAnalyzer (23) enable convenient access of the multiple targets of individual medicinal plants used in TCM and the regulated diseases by these plants. However, the targets are not grouped into functional categories and no regulated pathways and biological processes are given. These groupings and the systems-level information are highly useful for deciphering the complex multi-target activities of multiple ingredients and the underlying mechanisms of the functions of the individual plants. Moreover, these two databases cover a limited number (499 and 618, respectively) of TCM medicinal plants, lack the medicinal plants from other traditional medicines (e.g. Indian Ayurveda and Japanese Kampo) or used by folks of other regions (e.g. European and African countries) and are without coverage of other classes of functionally useful plants such as food, edible and garden plants are not covered. These limitations restrict our ability to analyze the collective activities and the functional implications of the multiple ingredients of individual plants from more diverse plant groups.

Hence, there is a need for a resource to conveniently access the information about individual plant’s multiple targets and collective activities of its ingredients, and the regulated biological pathways, biological processes and diseases for more diverse ranges of functionally useful plants. To complement the existing plant databases for catering this need, we developed a new database, Collective Molecular Activities of Useful Plants (CMAUP), to provide the collective landscapes of multiple targets (grouped into ChEMBL target classification classes, e.g. G Protein-Coupled Receptors), activity profiles (displayed in 2D target-ingredient heatmap), gene ontologies (grouped into gene ontology GO categories, e.g. GO:0002376 immune system process), biological pathways (grouped into KEGG pathway categories top and second level, e.g. ‘Environmental Information Processing’ and ‘Signal transduction’) and diseases (grouped into the international classification of disease ICD-10 blocks) for the classes of medicinal, food, edible, agricultural (exclude food and edible) and garden plants. The relevant information was extracted from the literature and the established internet resources of functionally useful plants, natural products, activities of natural products, proteins, gene ontologies, biological pathways and diseases. The facilities were set-up for convenient access of CMAUP by multiple search modes including keywords, plant usage classes, species families, targets, KEGG pathways, gene ontologies, diseases (ICD code) and geographical locations.

## DATA COLLECTION AND PROCESSING

The functionally useful plants were searched from the internet sources, books and literature database PubMed (28) by using the keywords ‘medicinal’, ‘food’, ‘edible’, ‘agricultural’ and ‘garden’ in combination with the keyword ‘plant’ or ‘plants’. The searched sources must be scientific journals, credible databases, books or official reports of government agencies, botanic gardens, international scientist networks and charitable organizations. Only those plants with the experimentally determined target and quantitative activity value were included in CMAUP. The geographic location of each plant is the location of plant collection or an indigenous medicine reported by the source or literature. The ingredients of the searched plants were from the literature (1562 research articles), published natural product databases or plant databases that provide the ingredients of individual plants reported in a publication.

The protein targets and the quantitative activity values of these plant ingredients were from the literatures and published databases that describe the experimentally determined targets and activity values of natural products reported in a research article. The quantitative activity value may be measured as half-maximal inhibition concentration IC50, equilibrium inhibition constant Ki, half-maximal effective concentration EC50, half-maximal activity concentration AC50, half-maximal growth inhibitory concentration GI50 and potency of half-maximal effect. The disease and its ICD-10 code regulated by each protein target was(were) from Therapeutic Target Database (TTD) (29) and the corresponding ChEMBL target classification class was from ChEMBL (30). The GO category and KEGG pathway affiliated with each target were generated from the enrichment tools GOATOOLS (31) and Enrichr python package (32). Crosslinks to the relevant databases (Plants of the World Online (http://www.plantsoftheworldonline.org), NCBI Taxonomy (33), PubChem (34), ZINC (35), ChEMBL (30), UniProt, (36), QuickGO (37), KEGG (38), TTD (29), PubMed (28) and doi.org) were curated and provided.

The 2D target-ingredient heatmap was created as follows: an activity matrix *M* of the ingredient-target activities was created with the matrix element *M*_ij_ representing the activity value of a plant ingredient *i* against a protein target *j*. The ‘heatmap.2’ R package was used to cluster the activity matrix *M* with respect to the targets using the default parameters (Distance method: ‘Euclidean’; Hierarchical cluster linkage method: ‘complete’) (https://cran.r-project.org/web/packages/gplots/gplots.pdf). The activity matrix after hierarchical clustering was displayed on the web interface by using the ‘HighCharts’ heatmap JavaScript library (https://www.highcharts.com).

## DATABASE CONTENTS, STRUCTURE AND ACCESS

**Figure 1.**
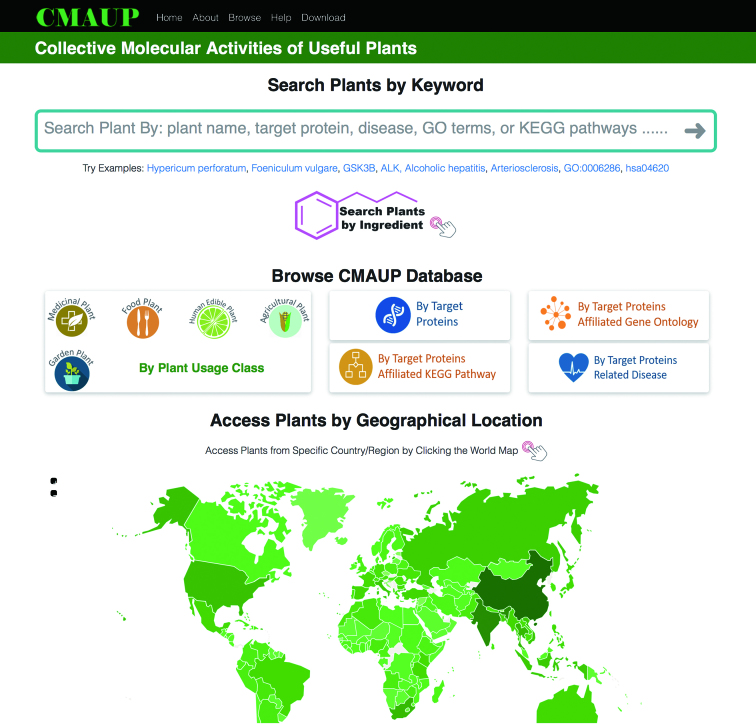
The homepage of CMAUP web interface. The webpage allows users to search plants by keywords (e.g. plant name, target, disease, pathway), search plants by ingredients, browse plants by plant usage classes and other options, and access plants by the interactive world map.

CMAUP (freely accessible at http://bidd2.nus.edu.sg/CMAUP/, the main webpage in Figure 1) currently contains 5645 plants (references in Supplementary Table S1), 70.2 and 99.9% of which are with identifiable taxonomic information at species and genus level, respectively, which were obtained through matching species name against NCBI Taxonomy database (33). The 5645 plants include 2567 medicinal, 170 food, 1567 edible (exclude food plants), 3 agricultural (exclude food and edible plants) and 119 garden plants collected from or medically used in 153 countries/regions. Medicinal plants include the herbs of indigenous medicines such as TCM, traditional Indian medicines (folk, Ayurveda, Siddha, Unani, Sowa-Rigpa, Homeopathy) and Japanese Kampo and the plants used for medicinal purposes in various 26 Asian, 19 African, 23 European, 8 North and South American and 3 Oceania countries, which were from a number of established databases summarized in Supplementary Table S1.

Food plants are the plants used as foods, flavours or preservatives by sizable populations or are edible with higher nutritional values, which were primarily from the European Tea Committee and European Herbal Infusions Association’s report ‘THIE Inventory List of Herbals Considered as Food’, the book chapter ‘Economic Botany- Plants as Food, and the websites of Food Plants International (http://foodplantsinternational.com) and the Gardener’s network (http://gardenersnet.com/gardening/plantbotanicalnames.htm#vegetables). Edible plants (exclude food plants) are the plants used by small population of folks as foods, flavours and preservatives and with less nutritional values, which were mainly from the Plants For A Future (https://pfaf.org) and KANpSAcK (13) databases. Agricultural plants (exclude food and edible plants) are the plants grown by humans for commercial purposes (e.g. timber and rubber), which were from European Commission’s Plant variety database (http://ec.europa.eu/food/plant/plant_propagation_material/ plant_variety_catalogues_databases/search/public/index.cfm), Plant Encyclopedia (https://www.bhg.com/gardening/plant-dictionary), and Wiki pages. Garden plants are for public, commercial and home gardens and landscapes, which were from the gardener’s network (http://gardenersnet.com/gardening/plantbotanicalnames.htm#flowers), Cornell University Home Garden-Based Learning (http://www.gardening.cornell.edu/homegardening), Japanese Garden Plants (http://www.jgarden.org/plants.asp) and Wiki Garden plants of Europe (https://en.wikipedia.org/wiki/Category:Garden_plants_of_Europe).

There are 6892 and 2275 ingredients from 5645 plants that are with both a target and experimentally measured activity value of IC50, Ki, EC50, AC50, GI50 or potency <10 μM (strong and moderate potency) and 10–50 μM (low potency), respectively. The 6892 strong and moderate potency ingredients are active against 646 human protein targets, which are grouped into 73 ChEMBL target classification level-3 classes (e.g. GSK3B in the ‘Protein Kinase’ class). The 646 targets are affiliated with 234 KEGG pathways, which are classified into the 7 top-level categories (e.g. mTOR signaling pathway in the ‘Environmental Information Processing’ category) and 39 second-level categories (e.g. mTOR signaling pathway in the ‘Signal transduction’ category) based on KEGG Orthology. The 646 targets are associated with 2473 gene ontologies, which are grouped into 41 GO categories. Also, 332 of these targets are associated with 656 diseases (a target-disease association means that the target has been reported as a therapeutic target for the disease treatment based on TTD), which are classified into 21 disease ICD-10 code blocks.

The 2275 experimentally determined low-potency ingredients and their targets are also provided in the respective plant ingredient page of CMAUP. Although some of these ingredients are expected to contribute to the collective activities and observed functions of their host plants, a large percentage of them unlikely make significant contribution. A comprehensive study of the synergistic combinations of sub-potent natural products has shown that only a small percentage of the synergistic combinations of sub-potent natural products produce drug-level potent activities (39). Therefore, additional information is needed for selecting those low-potency ingredients with experimentally confirmed or computationally demonstrated ability in producing significantly enhanced collective activities in combination with other ingredients of the same plant. The collection of such information is ongoing and expectedly takes some time. Hence, in the current version of CMAUP, the possible contribution of these ingredients to the collective activities of their host plants is tentatively un included until the completion of our collection works.

There are 47 645 known ingredients derived from the 5645 plants, 38 478 of them are without a reported activity value. To facilitate the investigations of these ingredients, CMAUP provides the experimentally determined targets of the molecules in the ChEMBL database (30) that are structurally similar to each of the 37 712 plant ingredients. These structurally similar molecules were generated by the similarity searching method, which is based on the hypothesis that structurally similar molecules tend to have similar activities (40). Hence, the targets of structurally similar molecules may indicate the potential targets or target classes of the plant ingredients. In our method, the chemical structures are represented by PubChem molecular fingerprints (41,42) and structural similarity between molecules is measured by Tanimoto coefficient (40,42). The targets of these structurally similar molecules are provided in the lower section of the target page of each plant.

**Figure 2.**
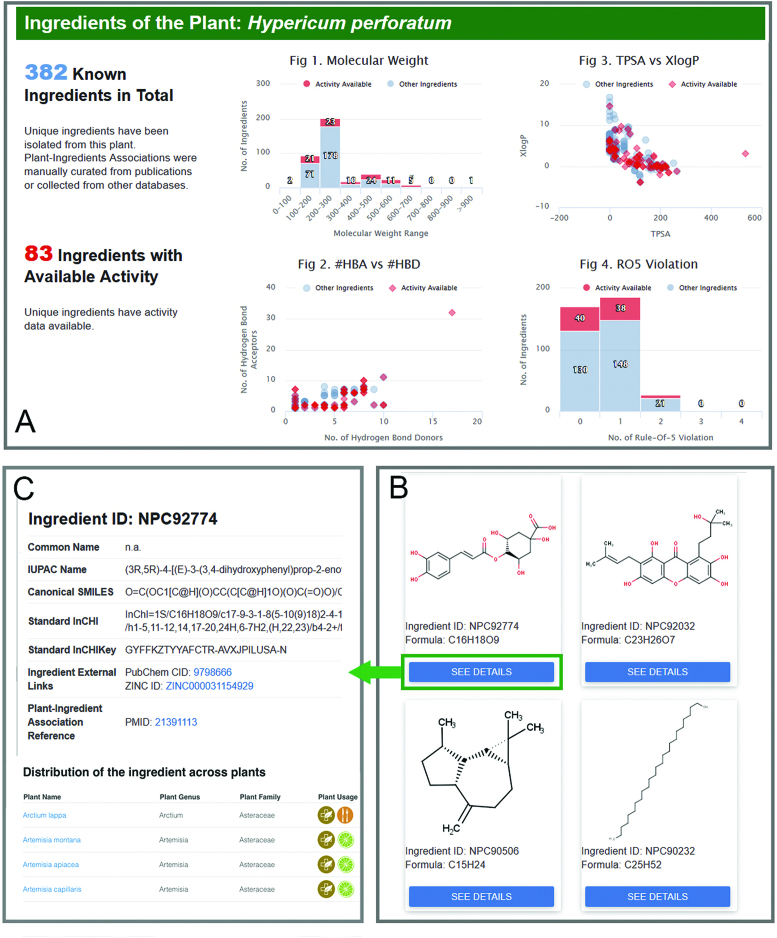
Ingredients of a specific plant. (**A**) Chemical ingredients are shown in the graphs based on distributions of few important physiochemical properties (e.g. molecular weight, XlogP, number of hydrogen bond donor). (**B**) Chemical structure images are provided when available. The chemical information, reference of plant-ingredient association and distribution of the ingredient across plants can be accessed by click the ‘SEE DETAILS’ button (**C**).

**Figure 3.**
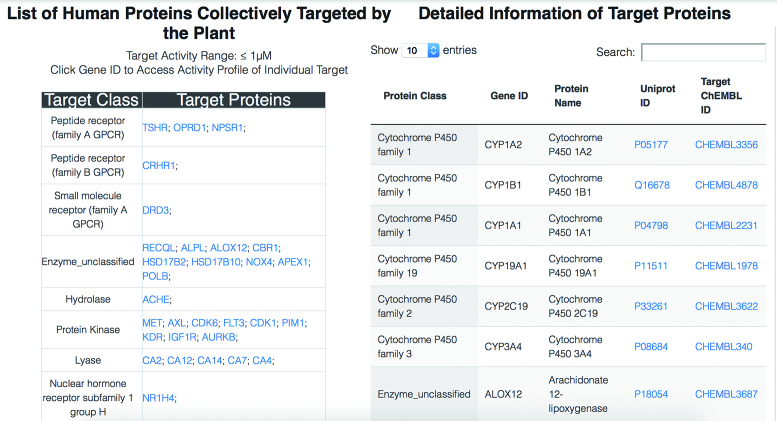
An example webpage of protein targets of a specific plant. Target proteins are categorized into classes based on ChEMBL target classification. External links are provided in the table in the right pane.

For each user-searched plant, its ingredients are visualized in physiochemical property spaces and the reference of each individual plant-ingredient association is provided in the popup window of each chemical structure (Figure 2). The landscape of the multiple targets of its ingredients (Figure 3) is generated with a default target activity cut-off value of IC50, Ki, EC50, AC50, GI50 or potency ≤1 μM. This cut-off gives rise to a landscape of nanomolar range potent activities, which ensures the landscape can reveal some of the observable effects of the plant. It is noted that the synergistic effects of multiple natural products may involve moderate (e.g. sub-micromolar range) activities (39). Therefore, an optional cut-off value of IC50, Ki, EC50, AC50, GI50 or potency <10 μM was introduced for generating an alternative target landscape to cater the need for the study of the contribution of moderate as well as potent activities. CMAUP also provide the overview of the target activity landscapes across all the plant species. Users can access the webpage of this cross-species target landscape by clicking the relevant button in the target page of each individual plant. Moreover, the distribution of each individual ingredient across plant species is also provided in CMAUP. Users can access the webpage of this cross-species ingredient distribution profile by clicking the relevant button in the ingredient page of each individual plant.

**Figure 4.**
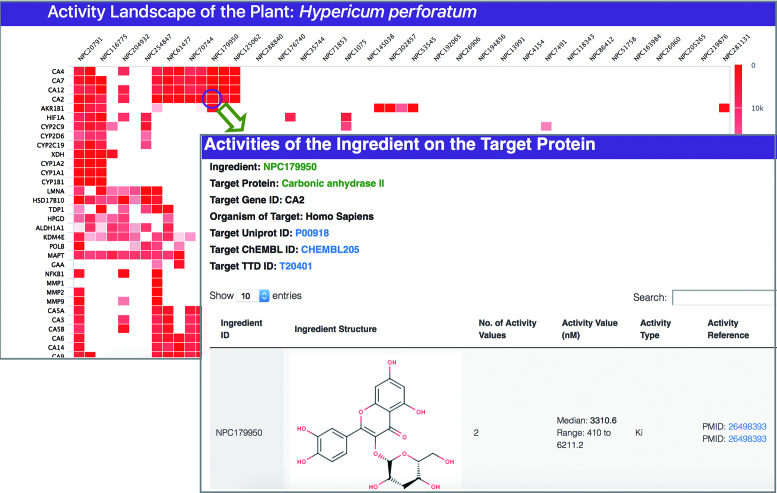
Activity landscape of a specific plant. The interactive heatmap allows users to access the detailed information of activity by clicking the individual cells.

**Figure 5.**
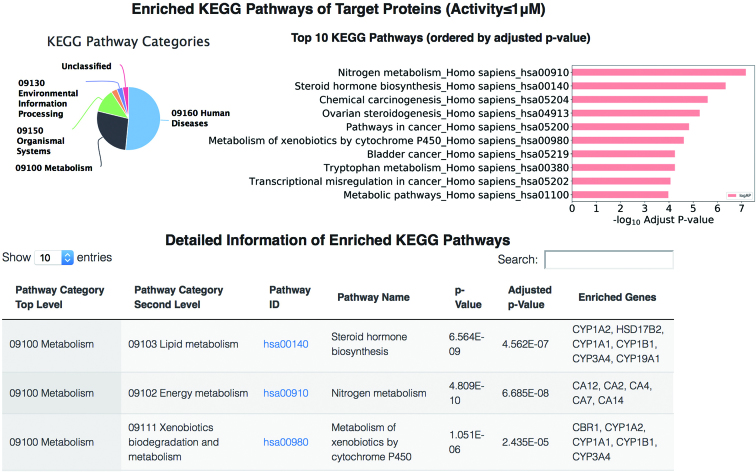
KEGG pathways of a specific plant. The pie chart shows the distribution of pathways in each KEGG Orthology category. The bar chart presents the top 10 enriched pathways ordered by the adjusted *P*-values. The bottom table lists detailed information of enriched KEGG pathways.

**Figure 6.**
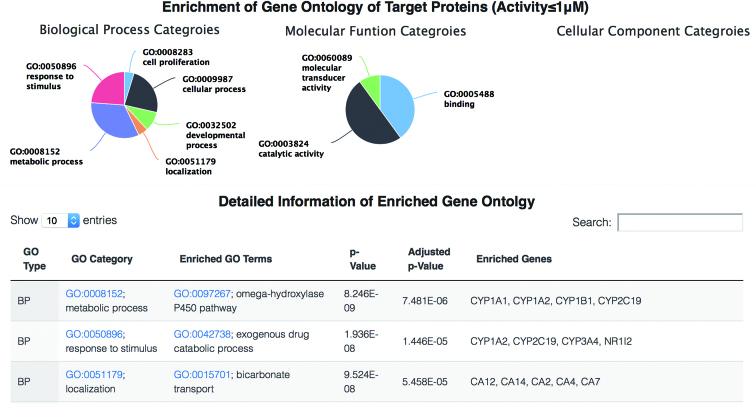
Gene Ontology terms of a specific plant. Distributions of GO terms in biological process, molecular function and cellular component categories are displayed in the top pie charts. The bottom table lists detailed information about enriched GO terms.

**Figure 7.**
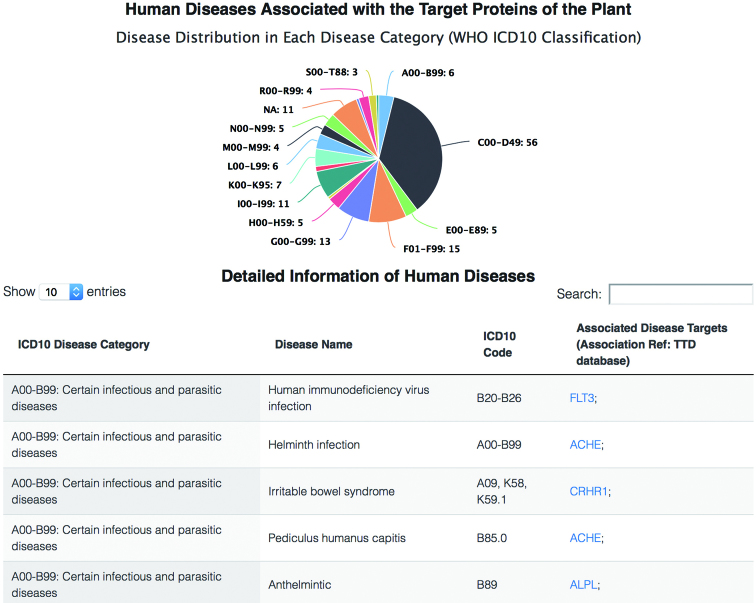
Human diseases related to a specific plant. The upper pie chart shows the number of diseases in each WHO ICD-10 disease category. The below table lists detailed information about these diseases.

The landscape of the target-ingredient activity profiles is further displayed by the 2D target-ingredient heatmap (Figure 4). In this heatmap, the activity values of the plant ingredients are clustered against their respective targets, with each red-colored small-block representing the activity value (unit of nM) of an individual ingredient against a target and the brightness of the red-color indicating the level of the activity (the higher activity with the darker color). Users can click the heatmap block to access the detailed information of the activities, including the referenced literature where the activity was reported. Based on the associations of the multiple targets of an individual plant with biological pathway, gene ontology and disease, the corresponding landscapes of the KEGG pathways (Figure 5), GO categories (Figure 6) and disease ICD-10 blocks (Figure 7) were also provided.

CMAUP entries can also be searched by plant ingredients. Multiple search options are provided. These include chemical name, chemical SMILES string and the user-drawing chemical structure by using an on-screen chemical structure drawing tool JSME (43) installed in the relevant structure search section of the CMAUP webpage. JSME is a well-established free molecule editor in JavaScript with multiple functionalities including substituent menu, copy/paste, drag, drop and undo/redo operations. The search options of SMILES strings and user-drawing structures are particularly useful for searching ingredients of unique chemical structures in the situation that an individual ingredient has multiple names or identifiers. Moreover, the structural search option returns additional set of ingredients that are structurally similar to the user-input molecule. These structurally similar ingredients are generated by a similarity searching (40) back-end engine wherein the chemical structures are represented by PubChem molecular fingerprints (41,42) and structural similarity between molecules is measured by Tanimoto coefficient (40,42).

CMAUP was developed on MySQL database and PHP server software. Its web-interfaces were built by using HTML, PHP and JavaScript, and were designed to enable the access of its entries by browsing or searching plant name, species family, plant usage class (e.g. medicinal, food, edible, garden plant), target gene name, target gene ontology (GO categories), target-regulated KEGG pathway and target-regulated disease. While applicable, the plant entries are cross-linked to xxx and NCBI Taxonomy database, ingredient entries are crosslinked to PubChem, ChEMBL, ZINC and SuperNatural (44) databases, and the target genes crosslinked to UniProt database. The references for the plant ingredients and their targets and activity values are also provided in the respective entry pages. All data can be freely and conveniently downloaded from the respective entry page. A download summary section provides hyperlinks for users to download data of interest by selecting specific data groups. Specifically, options are provided for users to download the data in the groups of all plants, all targets, all ingredients, all active ingredients, all plant-ingredient associations and all ingredient-target activity records, respectively.

## PERSPECTIVES

Functionally useful plants have been extensively studied and explored for broader applications from multiple perspectives including the species phylogenies (45), phytochemical informatics (46), altered metabolisms by multi-gene and pathway manipulations (5,6), improved activities by multi-targeted systems regulations (11,39), reverse pharmacokinetics (47), CMAUP complements existing plant related databases (13–27) for serving the expanding needs in plant research and development efforts by providing convenient access of the information about individual plant’s multiple targets and collective activities of its ingredients, and the regulated biological pathways, biological processes and diseases for more diverse ranges of functionally useful plants.

The rich collections of activity profiles of the plant ingredients in CMAUP may be combined with the plant metabolomics data (48) for facilitating the annotation of plant metabolomics (49,50) and for the interpretation of metabolic data in plant natural product discovery (51). CMAUP data may also be useful for facilitating other studies. These include the study of the metabolite distribution across plant species (52), the analysis of the relationships of the plant species (53), phylogenies (54), phytochemicals (55) and functions (56) across geographical regions and environmental conditions. There is a need for more comprehensive literature mining for the multi-target activities of multiple ingredients of more diverse ranges of functionally useful plants, and the incorporation of these profiles into this and other relevant databases.

## Supplementary Material

Supplementary Data
