# Supplementary material for: CMAUP: a database of collective molecular activities of useful plants
Source: Nucleic Acids Res. 2018 Oct 24;47(Database issue):D1118–27. doi: 10.1093/nar/gky965 (PMC6324012; doi:10.1093/nar/gky965)
Supplement: Supplementary Data [file gky965_supplemental_files.pdf]

Supplementary Material

CMAUP: A Database of Collective Molecular Activities of Useful Plants

Xian Zeng[1,2], Peng Zhang[2], Yali Wang[2], Chu Qin[2], Shangying Chen[2], Weidong He[2], Lin Tao[2,5], Ying Tan[1], Dan Gao[1], Bohua Wang[3,4], Zhe Chen[5], Weiping Chen[4*], Yu Yang Jiang[1*], Yu Zong Chen[2*]

[1]The State Key Laboratory of Chemical Oncogenomics, Key Laboratory of Chemical Biology, Tsinghua University Shenzhen Graduate School, Shenzhen Technology and Engineering Laboratory for Personalized Cancer Diagnostics and Therapeutics, Shenzhen Kivita Innovative Drug Discovery Institute, Guangdong, P. R. China.

[2]Bioinformatics and Drug Design group, Department of Pharmacy, National University of Singapore, Singapore 117543, Singapore.

[3]Key Lab of Agricultural Products Processing and Quality Control of Nanchang City, Jiangxi Agricultural University, Nanchang, 330045, P. R. China.

[4]College of Life and Environmental Sciences, Collaborative Innovation Center for Efficient and Health Production of Fisheries in Hunan Province, Hunan University of Arts and Science, Changde, Hunan, 415000, P. R. China.

[5]Zhejiang Key Laboratory of Gastro-intestinal Pathophysiology, Zhejiang Hospital of Traditional Chinese Medicine, Zhejiang Chinese Medical University, School of Medicine, Hangzhou Normal University, Hangzhou 310006, R. P. China.

* To whom correspondence should be addressed. Y.Z. Chen Tel: +65 6516 6877; Fax: +65 6774 6756; Email: phacyz@nus.edu.sg.

Correspondence may also be addressed to Y.Y. Jiang Tel: +86 755 2603 6430; Fax: +86 755 2603 6430; Email: jiangyy@sz.tsinghua.edu.cn and W.P. Chen Tel.:+86 791 8381 3420. Fax: +86 791 8381 3655. E-mail: iaochen@163.com.

Supplementary Table S1. List of databases and research articles used in this work to collect medicinal, food, human edible, agricultural, and garden plants as well as chemical ingredients of all plants.

**Databases:**	
**Database Name**	**Database URL**
NPASS	http://bidd2.nus.edu.sg/NPASS/
TCM-ID	http://bidd.nus.edu.sg/group/TCMsite/Default.aspx
UNPD	http://pkuxxj.pku.edu.cn/UNPD/
TCMID	http://www.megabionet.org/tcmid/
TCM@Taiwan	http://tcm.cmu.edu.tw/
TCMSP	http://lsp.nwu.edu.cn/tcmsp.php
TM-MC	http://informatics.kiom.re.kr/compound/
KANpSAcK	http://kanaya.naist.jp/KNApSAcK_World/top.jsp
HerDing	http://combio.gist.ac.kr/herding
Food Plants International	http://foodplantsinternational.com
Indian Medicinal Plants Database	http://www.medicinalplants.in/
Metabolomics.jp	http://metabolomics.jp/wiki/Persist:CrudeDrugList

Plant Encyclopedia	https://www.bhg.com/gardening/plant-dictionary/
Plants of the world online	http://www.plantsoftheworldonline.org/
Plants For A Future	https://pfaf.org/
European Commission's Plant variety database	http://ec.europa.eu/food/plant/plant_propagation_material/ plant_variety_catalogues_databases/search/public/index.cfm
Cornell University Garden-Based Learning	http://www.gardening.cornell.edu/homegardening/
Gardener's network	http://gardenersnet.com/gardening/plantbotanicalnames.htm
Japanese Garden Plants	http://www.jgarden.org/plants.asp
Plant Encyclopedia	https://www.bhg.com/gardening/plant-dictionary/
Wiki Garden plants of Europe	https://en.wikipedia.org/wiki/Category:Garden_plants_of_Europe

Journal Articles:

1. Abdallah, I.B., Tlili, N., Martinez-Force, E. *et al.* (2015) Content of carotenoids, tocopherols, sterols, triterpenic and aliphatic alcohols, and volatile compounds in six walnuts (Juglans regia L.) varieties. *Food Chem*, **173**, 972-978.
2. Abdel-Halim, O.B., Morikawa, T., Ando, S. *et al.* (2004) New crinine-type alkaloids with inhibitory effect on induction of inducible nitric oxide synthase from Crinum yemense. *J Nat Prod*, **67**, 1119-1124.
3. Abdel-Kader, M., Berger, J.M., Slebodnick, C. *et al.* (2002) Isolation and absolute configuration of ent-Halimane diterpenoids from Hymenaea courbaril from the Suriname rain forest. *J Nat Prod*, **65**, 11-15.
4. Abe, I., Kashiwagi, Y., Noguchi, H. *et al.* (2001) Ellagitannins and hexahydroxydiphenoyl esters as inhibitors of vertebrate squalene epoxidase. *J Nat Prod*, **64**, 1010-1014.
5. Aboul-Ela, M.A., El-Lakany, A.M. and Hammoda, H.M. (2004) Alkaloids from the bulbs of Crinum bulbispermum. *Pharmazie*, **59**, 894-896.
6. Acebey-Castellon, I.L., Voutquenne-Nazabadioko, L., Doan Thi Mai, H. *et al.* (2011) Triterpenoid saponins from Symplocos lancifolia. *J Nat Prod*, **74**, 163-168.
7. Acharya, D., Mitaine-Offer, A.C., Kaushik, N. *et al.* (2009) Cytotoxic spirostane-type saponins from the roots of Chlorophytum borivilianum. *J Nat Prod*, **72**, 177-181.
8. Acuna, U.M., Figueroa, M., Kavalier, A. *et al.* (2010) Benzophenones and biflavonoids from Rheedia edulis. *J Nat Prod*, **73**, 1775-1779.
9. Adams, M., Pacher, T., Greger, H. *et al.* (2005) Inhibition of leukotriene biosynthesis by stilbenoids from Stemona species. *J Nat Prod*, **68**, 83-85.
10. Adelekan, A.M., Prozesky, E.A., Hussein, A.A. *et al.* (2008) Bioactive diterpenes and other constituents of Croton steenkampianus. *J Nat Prod*, **71**, 1919-1922.
11. Adeoye, A.O., Oguntimein, B.O., Clark, A.M. *et al.* (1986) 3-Dimethylallylindole: an antibacterial and antifungal metabolite from Monodora tenuifolia. *J Nat Prod*, **49**, 534-537.
12. Adnyana, I.K., Tezuka, Y., Banskota, A.H. *et al.* (2001) Three new triterpenes from the seeds of Combretum quadrangulare and their hepatoprotective activity. *J Nat Prod*, **64**, 360-363.
13. Adnyana, I.K., Tezuka, Y., Banskota, A.H. *et al.* (2000) Quadranosides I-V, new triterpene glucosides from the seeds of Combretum quadrangulare. *J Nat Prod*, **63**, 496-500.
14. Adrian, M., Jeandet, P., Douillet-Breuil, A.C. *et al.* (2000) Stilbene content of mature Vitis vinifera berries in response to UV-C elicitation. *J Agric Food Chem*, **48**, 6103-6105.
15. Ahmed, S.A., Ross, S.A., Slade, D. *et al.* (2008) Cannabinoid ester constituents from high-potency Cannabis sativa. *J Nat Prod*, **71**, 536-542.
16. Ahn, B.T., Lee, S., Lee, S.B. *et al.* (2001) Low-density lipoprotein-antioxidant constituents of Saururus chinensis. *J Nat Prod*, **64**, 1562-1564.
17. Ahn, H., Kim, J.Y., Lee, H.J. *et al.* (2003) Inhibitors of inducible nitric oxide synthase expression from Artemisia iwayomogi. *Arch Pharm Res*, **26**, 301-305.
18. Ahn, J.H., Kim, E.S., Lee, C. *et al.* (2013) Chemical constituents from Nelumbo nucifera leaves and their anti-obesity effects. *Bioorg Med Chem Lett*, **23**, 3604-3608.
19. Ahn, M.J., Kim, C.Y., Yoon, K.D. *et al.* (2006) Steroidal saponins from the rhizomes of Polygonatum sibiricum. *J Nat Prod*, **69**, 360-364.
20. Ahn, M.J., Lee, M.K., Kim, Y.C. *et al.* (2008) The simultaneous determination of coumarins in Angelica gigas root by high performance liquid chromatography-diode array detector coupled with electrospray ionization/mass spectrometry. *J Pharm Biomed Anal*, **46**, 258-266.
21. Ajjawi, I., Rodriguez Milla, M.A., Cushman, J. *et al.* (2007) Thiamin pyrophosphokinase is required for thiamin cofactor activation in Arabidopsis. *Plant Mol Biol*, **65**, 151-162.
22. Akihisa, T., Kithsiri Wijeratne, E.M., Tokuda, H. *et al.* (2002) Eupha-7,9(11),24-trien-3beta-ol ("antiquol C") and other triterpenes from Euphorbia antiquorum latex and their inhibitory effects on Epstein-Barr virus activation. *J Nat Prod*, **65**, 158-162.

23. Akihisa, T., Matsumoto, K., Tokuda, H. *et al.* (2007) Anti-inflammatory and potential cancer chemopreventive constituents of the fruits of Morinda citrifolia (Noni). *J Nat Prod*, **70**, 754-757.
24. Akihisa, T., Tokuda, H., Hasegawa, D. *et al.* (2006) Chalcones and other compounds from the exudates of Angelica keiskei and their cancer chemopreventive effects. *J Nat Prod*, **69**, 38-42.
25. Al-Baghdadi, O.B., Prater, N.I., Van der Schyf, C.J. *et al.* (2012) Inhibition of monoamine oxidase by derivatives of piperine, an alkaloid from the pepper plant Piper nigrum, for possible use in Parkinson's disease. *Bioorg Med Chem Lett*, **22**, 7183-7188.
26. Alabdul Magid, A., Voutquenne, L., Harakat, D. *et al.* (2006) Triterpenoid saponins from the fruits of Caryocar villosum. *J Nat Prod*, **69**, 919-926.
27. Alali, F., El-Elimat, T., Albataineh, H. *et al.* (2015) Cytotoxic Homoisoflavones from the Bulbs of Bellevalia eigii. *J Nat Prod*, **78**, 1708-1715.
28. Alali, F.Q., El-Elimat, T., Li, C. *et al.* (2005) New colchicinoids from a native Jordanian meadow saffron, colchicum brachyphyllum: isolation of the first naturally occurring dextrorotatory colchicinoid. *J Nat Prod*, **68**, 173-178.
29. Alali, F.Q., Zhang, Y., Rogers, L. *et al.* (1997) (2,4-cis and trans)-gigantecinone and 4-deoxygigantecin, bioactive nonadjacent bis-tetrahydrofuran annonaceous acetogenins, from Goniothalamus giganteus. *J Nat Prod*, **60**, 929-933.
30. Ali, M.S., Tezuka, Y., Awale, S. *et al.* (2001) Six new diarylheptanoids from the seeds of Alpinia blepharocalyx. *J Nat Prod*, **64**, 289-293.
31. Ali, M.S., Tezuka, Y., Banskota, A.H. *et al.* (2001) Blepharocalyxins C--E, three new dimeric diarylheptanoids, and related compounds from the seeds of Alpinia blepharocalyx. *J Nat Prod*, **64**, 491-496.
32. Aliferis, K.A., Faubert, D. and Jabaji, S. (2014) A metabolic profiling strategy for the dissection of plant defense against fungal pathogens. *PLoS One*, **9**, e111930.
33. Amezqueta, S., Galan, E., Fuguet, E. *et al.* (2012) Determination of D-fagomine in buckwheat and mulberry by cation exchange HPLC/ESI-Q-MS. *Anal Bioanal Chem*, **402**, 1953-1960.
34. An, S., Park, Y.D., Paik, Y.K. *et al.* (2007) Human ACAT inhibitory effects of shikonin derivatives from Lithospermum erythrorhizon. *Bioorg Med Chem Lett*, **17**, 1112-1116.
35. Andersen, R.J. and Taglialatela-Scafati, O. (2005) Avrainvilloside, a 6-deoxy-6-aminoglucoglycerolipid from the green alga Avrainvillea nigricans. *J Nat Prod*, **68**, 1428-1430.
36. Angerhofer, C.K., Guinaudeau, H., Wongpanich, V. *et al.* (1999) Antiplasmodial and cytotoxic activity of natural bisbenzylisoquinoline alkaloids. *J Nat Prod*, **62**, 59-66.
37. Anis, E., Anis, I., Ahmed, S. *et al.* (2002) Alpha-glucosidase inhibitory constituents from Cuscuta reflexa. *Chem Pharm Bull (Tokyo)*, **50**, 112-114.
38. Ankisetty, S., ElSohly, H.N., Li, X.C. *et al.* (2006) Aromatic constituents of Uvaria grandiflora. *J Nat Prod*, **69**, 692-694.
39. Aoki, N., Muko, M., Ohta, E. *et al.* (2008) C-geranylated chalcones from the stems of Angelica keiskei with superoxide-scavenging activity. *J Nat Prod*, **71**, 1308-1310.
40. Appendino, G., Gibbons, S., Giana, A. *et al.* (2008) Antibacterial cannabinoids from Cannabis sativa: a structure-activity study. *J Nat Prod*, **71**, 1427-1430.
41. Appendino, G., Pollastro, F., Verotta, L. *et al.* (2009) Polyacetylenes from sardinian Oenanthe fistulosa: a molecular clue to risus sardonicus. *J Nat Prod*, **72**, 962-965.
42. Aquino, R., De Feo, V., De Simone, F. *et al.* (1991) Plant metabolites. New compounds and anti-inflammatory activity of Uncaria tomentosa. *J Nat Prod*, **54**, 453-459.
43. Aquino, R., De Simone, F., Pizza, C. *et al.* (1989) Plant metabolites. Structure and in vitro antiviral activity of quinovic acid glycosides from Uncaria tomentosa and Guettarda platypoda. *J Nat Prod*, **52**, 679-685.
44. Arai, M.A., Fujimatsu, T., Uchida, K. *et al.* (2013) Hh signaling inhibitors from Vitex negundo; naturally occurring inhibitors of the GLI1-DNA complex. *Mol Biosyst*, **9**, 1012-1018.
45. Arai, M.A., Uchida, K., Sadhu, S.K. *et al.* (2015) Hedgehog inhibitors from Artocarpus communis and Hyptis suaveolens. *Bioorg Med Chem*, **23**, 4150-4154.
46. Arciniegas, A., Gonzalez, K., Perez-Castorena, A.L. *et al.* (2011) Seco-eremophiladiolides and eremophilane glucosides from Pittocaulon velatum. *J Nat Prod*, **74**, 1584-1589.
47. Arisawa, M., Fujita, A., Morita, N. *et al.* (1985) Plant Anticancer Agents XXXV. Further Constituents of Simaba multiflora1,2. *Planta Med*, **51**, 348-349.
48. Arisawa, M., Kinghorn, A.D., Cordell, G.A. *et al.* (1983) Plant anticancer agents. XXIV. Alkaloid constituents of Simaba multiflora. *J Nat Prod*, **46**, 222-225.
49. Arslanian, R.L., Bailey, D.T., Kent, M.C. *et al.* (1995) Brevitaxin, a new diterpenolignan from the bark of Taxus brevifolia. *J Nat Prod*, **58**, 583-585.

50. Asada, Y., Sukemori, A., Watanabe, T. *et al.* (2013) Isolation, structure determination, and anti-HIV evaluation of tigliane-type diterpenes and biflavonoid from Stellera chamaejasme. *J Nat Prod*, **76**, 852-857.
51. Asano, N., Kato, A., Miyauchi, M. *et al.* (1998) Nitrogen-containing furanose and pyranose analogues from Hyacinthus orientalis. *J Nat Prod*, **61**, 625-628.
52. Ashihara, H., Sano, H. and Crozier, A. (2008) Caffeine and related purine alkaloids: biosynthesis, catabolism, function and genetic engineering. *Phytochemistry*, **69**, 841-856.
53. Asili, J., Lambert, M., Ziegler, H.L. *et al.* (2004) Labdanes and isopimaranes from Platycladus orientalis and their effects on erythrocyte membrane and on Plasmodium falciparum growth in the erythrocyte host cells. *J Nat Prod*, **67**, 631-637.
54. Athanasas, K., Magiatis, P., Fokialakis, N. *et al.* (2004) Hyperjovinols A and B: Two new phloroglucinol derivatives from Hypericum jovis with antioxidant activity in cell cultures. *J Nat Prod*, **67**, 973-977.
55. Atta-ur-Rahman, A., Ata, A., Naz, S. *et al.* (1999) New steroidal alkaloids from the roots of buxus sempervirens. *J Nat Prod*, **62**, 665-669.
56. Atta ur, R., Nasreen, A., Akhtar, F. *et al.* (1997) Antifungal diterpenoid alkaloids from Delphinium denudatum. *J Nat Prod*, **60**, 472-474.
57. Atta ur, R., Naz, H., Fadimatou *et al.* (2005) Bioactive constituents from Boswellia papyrifera. *J Nat Prod*, **68**, 189-193.
58. Awale, S., Miyamoto, T., Linn, T.Z. *et al.* (2009) Cytotoxic constituents of Soymida febrifuga from Myanmar. *J Nat Prod*, **72**, 1631-1636.
59. Awale, S., Tezuka, Y., Banskota, A.H. *et al.* (2003) Nitric oxide inhibitory isopimarane-type diterpenes from Orthosiphon stamineus of Indonesia. *J Nat Prod*, **66**, 255-258.
60. Awale, S., Tezuka, Y., Banskota, A.H. *et al.* (2003) Siphonols A-E: novel nitric oxide inhibitors from Orthosiphon stamineus of Indonesia. *Bioorg Med Chem Lett*, **13**, 31-35.
61. Awang, K., Chan, G., Litaudon, M. *et al.* (2010) 4-Phenylcoumarins from Mesua elegans with acetylcholinesterase inhibitory activity. *Bioorg Med Chem*, **18**, 7873-7877.
62. Awouafack, M.D., Spiteller, P., Lamshoft, M. *et al.* (2011) Antimicrobial isopropenyl-dihydrofuranoisoflavones from Crotalaria lachnophora. *J Nat Prod*, **74**, 272-278.
63. Baek, N.I., Chung, M.S., Shamon, L. *et al.* (1993) Selligueain A, a novel highly sweet proanthocyanidin from the rhizomes of Selliguea feei. *J Nat Prod*, **56**, 1532-1538.
64. Baek, Y.S., Ryu, Y.B., Curtis-Long, M.J. *et al.* (2009) Tyrosinase inhibitory effects of 1,3-diphenylpropanes from Broussonetia kazinoki. *Bioorg Med Chem*, **17**, 35-41.
65. Baggett, S., Protiva, P., Mazzola, E.P. *et al.* (2005) Bioactive benzophenones from Garcinia xanthochymus fruits. *J Nat Prod*, **68**, 354-360.
66. Bai, N., Lai, C.S., He, K. *et al.* (2006) Sesquiterpene lactones from Inula britannica and their cytotoxic and apoptotic effects on human cancer cell lines. *J Nat Prod*, **69**, 531-535.
67. Baloch, I.B., Baloch, M.K. and Baloch, A.K. (2009) Bio-active compounds from Euphorbia cornigera Boiss. *Eur J Med Chem*, **44**, 3188-3194.
68. Baloch, I.B., Baloch, M.K. and Saqib, Q.N. (2008) Anti-tumor 12-deoxyphorbol esters from Euphorbia cornigera. *Eur J Med Chem*, **43**, 274-281.
69. Bang, S.C., Kim, Y., Lee, J.H. *et al.* (2005) Triterpenoid saponins from the roots of Pulsatilla koreana. *J Nat Prod*, **68**, 268-272.
70. Bankeu, J.J., Khayala, R., Lenta, B.N. *et al.* (2011) Isoflavone dimers and other bioactive constituents from the figs of Ficus mucuso. *J Nat Prod*, **74**, 1370-1378.
71. Banskota, A.H., Tezuka, Y., Phung, L.K. *et al.* (1998) Cytotoxic cycloartane-type triterpenes from Combretum quadrangulare. *Bioorg Med Chem Lett*, **8**, 3519-3524.
72. Banskota, A.H., Tezuka, Y., Tran, K.Q. *et al.* (2000) Thirteen novel cycloartane-type triterpenes from Combretum quadrangulare. *J Nat Prod*, **63**, 57-64.
73. Banskota, A.H., Usia, T., Tezuka, Y. *et al.* (2002) Three new C-14 oxygenated taxanes from the wood of Taxus yunnanensis. *J Nat Prod*, **65**, 1700-1702.
74. Bao, M.F., Yan, J.M., Cheng, G.G. *et al.* (2013) Cytotoxic indole alkaloids from Tabernaemontana divaricata. *J Nat Prod*, **76**, 1406-1412.
75. Barnes, E.C., Carroll, A.R. and Davis, R.A. (2011) Mitchellenes A-E, cyclic sesquiterpenes from the Australian plant Eremophila mitchellii. *J Nat Prod*, **74**, 1888-1893.
76. Barros, L.F., Barison, A., Salvador, M.J. *et al.* (2009) Constituents of the leaves of Magnolia ovata. *J Nat Prod*, **72**, 1529-1532.
77. Barros, L.F., Ehrenfried, C.A., Riva, D. *et al.* (2012) Essential oil and other constituents from Magnolia ovata fruit. *Nat Prod Commun*, **7**, 1365-1367.

78. Basset, G.J., Quinlivan, E.P., Ravanel, S. *et al.* (2004) Folate synthesis in plants: the p-aminobenzoate branch is initiated by a bifunctional PabA-PabB protein that is targeted to plastids. *Proc Natl Acad Sci U S A*, **101**, 1496-1501.
79. Baumgartner, L., Sosa, S., Atanasov, A.G. *et al.* (2011) Lignan derivatives from Krameria lappacea roots inhibit acute inflammation in vivo and pro-inflammatory mediators in vitro. *J Nat Prod*, **74**, 1779-1786.
80. Begum, S., Wahab, A., Siddiqui, B.S. *et al.* (2000) Nematicidal constituents of the aerial parts of Lantana camara. *J Nat Prod*, **63**, 765-767.
81. Beldjoudi, N., Mambu, L., Labaied, M. *et al.* (2003) Flavonoids from Dalbergia louvelii and their antiplasmodial activity. *J Nat Prod*, **66**, 1447-1450.
82. Belofsky, G., French, A.N., Wallace, D.R. *et al.* (2004) New geranyl stilbenes from Dalea purpurea with in vitro opioid receptor affinity. *J Nat Prod*, **67**, 26-30.
83. Benavides, A., Napolitano, A., Bassarello, C. *et al.* (2009) Oxylipins from Dracontium loretense. *J Nat Prod*, **72**, 813-817.
84. Berim, A. and Gang, D.R. (2013) Characterization of two candidate flavone 8-O-methyltransferases suggests the existence of two potential routes to nevadensin in sweet basil. *Phytochemistry*, **92**, 33-41.
85. Bernardi, A.P., Ferraz, A.B., Albring, D.V. *et al.* (2005) Benzophenones from Hypericum carinatum. *J Nat Prod*, **68**, 784-786.
86. Bhattacharya, A.K., Chand, H.R., John, J. *et al.* (2015) Clerodane type diterpene as a novel antifungal agent from Polyalthia longifolia var. pendula. *Eur J Med Chem*, **94**, 1-7.
87. Bibi, N., Tanoli, S.A., Farheen, S. *et al.* (2010) In vitro antituberculosis activities of the constituents isolated from Haloxylon salicornicum. *Bioorg Med Chem Lett*, **20**, 4173-4176.
88. Bickii, J., Njifutie, N., Foyere, J.A. *et al.* (2000) In vitro antimalarial activity of limonoids from Khaya grandifoliola C.D.C. (Meliaceae). *J Ethnopharmacol*, **69**, 27-33.
89. Bocar, M., Jossang, A. and Bodo, B. (2003) New alkaloids from Cephalotaxus fortunei. *J Nat Prod*, **66**, 152-154.
90. Bohlke, M., Guinaudeau, H., Angerhofer, C.K. *et al.* (1996) Costaricine, a new antiplasmodial bisbenzylisoquinoline alkaloid from Nectandra salicifolia trunk bark. *J Nat Prod*, **59**, 576-580.
91. Bohr, G., Gerhauser, C., Knauft, J. *et al.* (2005) Anti-inflammatory acylphloroglucinol derivatives from Hops (Humulus lupulus). *J Nat Prod*, **68**, 1545-1548.
92. Boonnak, N., Chantrapromma, S., Fun, H.K. *et al.* (2014) Three types of cytotoxic natural caged-scaffolds: pure enantiomers or partial racemates. *J Nat Prod*, **77**, 1562-1571.
93. Boonphong, S., Puangsombat, P., Baramee, A. *et al.* (2007) Bioactive compounds from Bauhinia purpurea possessing antimalarial, antimycobacterial, antifungal, anti-inflammatory, and cytotoxic activities. *J Nat Prod*, **70**, 795-801.
94. Boonsri, S., Karalai, C., Ponglimanont, C. *et al.* (2008) Cytotoxic and antibacterial sesquiterpenes from Thespesia populnea. *J Nat Prod*, **71**, 1173-1177.
95. Boukhris, M., Simmonds, M.S., Sayadi, S. *et al.* (2013) Chemical composition and biological activities of polar extracts and essential oil of rose-scented geranium, Pelargonium graveolens. *Phytother Res*, **27**, 1206-1213.
96. Bourjot, M., Delang, L., Nguyen, V.H. *et al.* (2012) Prostratin and 12-O-tetradecanoylphorbol 13-acetate are potent and selective inhibitors of Chikungunya virus replication. *J Nat Prod*, **75**, 2183-2187.
97. Bournine, L., Bensalem, S., Wauters, J.N. *et al.* (2013) Identification and quantification of the main active anticancer alkaloids from the root of Glaucium flavum. *Int J Mol Sci*, **14**, 23533-23544.
98. Bovi Mitre, G., Kamiya, N., Bardon, A. *et al.* (2004) Africane-type sesquiterpenoids from the Argentine liverwort Porella swartziana and their antibacterial activity. *J Nat Prod*, **67**, 31-36.
99. Bozic, D., Papaefthimiou, D., Bruckner, K. *et al.* (2015) Towards Elucidating Carnosic Acid Biosynthesis in Lamiaceae: Functional Characterization of the Three First Steps of the Pathway in Salvia fruticosa and Rosmarinus officinalis. *PLoS One*, **10**, e0124106.
100. Brandt, G.E., Schmidt, M.D., Prisinzano, T.E. *et al.* (2008) Gedunin, a novel hsp90 inhibitor: semisynthesis of derivatives and preliminary structure-activity relationships. *J Med Chem*, **51**, 6495-6502.
101. Brindis, F., Rodriguez, R., Bye, R. *et al.* (2011) (Z)-3-butylidenephthalide from Ligusticum porteri , an alpha-glucosidase inhibitor. *J Nat Prod*, **74**, 314-320.
102. Brinker, A.M., Ma, J., Lipsky, P.E. *et al.* (2007) Medicinal chemistry and pharmacology of genus Tripterygium (Celastraceae). *Phytochemistry*, **68**, 732-766.
103. Brophy, J.J., Forster, P.I. and Goldsack, R.J. (2016) Coconut Laurels: The Leaf Essential Oils from Four Endemic Australian Cryptocarya Species: C. bellendenkerana, C. cocosoides, C. cunninghamii and C. lividula (Lauraceae). *Nat Prod Commun*, **11**, 255-258.

104. Buchanan, M.S., Carroll, A.R., Edser, A. *et al.* (2008) Lysianadioic acid, a carboxypeptidase B inhibitor from Lysiana subfalcata. *Bioorg Med Chem Lett*, **18**, 1495-1497.
105. Bueno Perez, L., Li, J., Lantvit, D.D. *et al.* (2013) Bioactive constituents of Indigofera spicata. *J Nat Prod*, **76**, 1498-1504.
106. Bunel, V., Antoine, M.H., Nortier, J. *et al.* (2014) Protective effects of schizandrin and schizandrin B towards cisplatin nephrotoxicity in vitro. *J Appl Toxicol*, **34**, 1311-1319.
107. Caboni, P., Ntalli, N.G., Aissani, N. *et al.* (2012) Nematicidal activity of (E,E)-2,4-decadienal and (E)-2-decenal from Ailanthus altissima against Meloidogyne javanica. *J Agric Food Chem*, **60**, 1146-1151.
108. Caboni, P., Sarais, G., Aissani, N. *et al.* (2012) Nematicidal activity of 2-thiophenecarboxaldehyde and methylisothiocyanate from caper (Capparis spinosa) against Meloidogyne incognita. *J Agric Food Chem*, **60**, 7345-7351.
109. Cahlikova, L., Zavadil, S., Macakova, K. *et al.* (2011) Isolation and cholinesterase activity of Amaryllidaceae alkaloids from Nerine bowdenii. *Nat Prod Commun*, **6**, 1827-1830.
110. Cai, B.C., Hattori, M. and Namba, T. (1990) Processing of nux vomica. II. Changes in alkaloid composition of the seeds of Strychnos nux-vomica on traditional drug-processing. *Chem Pharm Bull (Tokyo)*, **38**, 1295-1298.
111. Cai, J.N., Basnet, P., Wang, Z.T. *et al.* (2000) Coumarins from the fruits of Cnidium monnieri. *J Nat Prod*, **63**, 485-488.
112. Cai, M., He, S., Shi, Y. *et al.* (2013) Rapid and sensitive analysis of euonine and wilforidine in human plasma by high-performance liquid chromatography-atmospheric-pressure chemical ionization-mass spectrometry. *J Anal Toxicol*, **37**, 395-400.
113. Cai, X.F., Jin, X., Lee, D. *et al.* (2006) Phenanthroquinolizidine alkaloids from the roots of Boehmeria pannosa potently inhibit hypoxia-inducible factor-1 in AGS human gastric cancer cells. *J Nat Prod*, **69**, 1095-1097.
114. Cai, X.F., Park, B.Y., Ahn, K.S. *et al.* (2009) Cytotoxic triterpenoids from the rhizomes of Astilbe chinensis. *J Nat Prod*, **72**, 1241-1244.
115. Calis, I., Kuruuzum, A., Demirezer, L.O. *et al.* (1999) Phenylvaleric acid and flavonoid glycosides from Polygonum salicifolium. *J Nat Prod*, **62**, 1101-1105.
116. Callies, O., Bedoya, L.M., Beltran, M. *et al.* (2015) Isolation, Structural Modification, and HIV Inhibition of Pentacyclic Lupane-Type Triterpenoids from Cassine xylocarpa and Maytenus cuzcoina. *J Nat Prod*, **78**, 1045-1055.
117. Callies, O., Sanchez-Canete, M.P., Gamarro, F. *et al.* (2015) Restoration of Chemosensitivity in P-Glycoprotein-Dependent Multidrug-Resistant Cells by Dihydro-beta-agarofuran Sesquiterpenes from Celastrus vulcanicola. *J Nat Prod*, **78**, 736-745.
118. Calzada, F., Cedillo-Rivera, R. and Mata, R. (2001) Antiprotozoal activity of the constituents of Conyza filaginoides. *J Nat Prod*, **64**, 671-673.
119. Candido, L.P., Varela, R.M., Torres, A. *et al.* (2016) Evaluation of the Allelopathic Potential of Leaf, Stem, and Root Extracts of Ocotea pulchella Nees et Mart. *Chem Biodivers*, **13**, 1058-1067.
120. Cantrell, C.L., Abate, L., Fronczek, F.R. *et al.* (1999) Antimycobacterial eudesmanolides from Inula helenium and Rudbeckia subtomentosa. *Planta Med*, **65**, 351-355.
121. Cantrell, C.L., Duke, S.O., Fronczek, F.R. *et al.* (2007) Phytotoxic Eremophilanes from Ligularia macrophylla. *J Agric Food Chem*, **55**, 10656-10663.
122. Cao, J. and Li, G. (2012) [Chemical constituents of Centipeda minima]. *Zhongguo Zhong Yao Za Zhi*, **37**, 2301-2303.
123. Cao, S., Guza, R.C., Miller, J.S. *et al.* (2004) Cytotoxic triterpenoids from Acridocarpus vivy from the Madagascar rain forest. *J Nat Prod*, **67**, 986-989.
124. Cao, Y., Chen, J.J., Tan, N.H. *et al.* (2010) Antimicrobial selaginellin derivatives from Selaginella pulvinata. *Bioorg Med Chem Lett*, **20**, 2456-2460.
125. Carroll, A.R., Lamb, J., Moni, R. *et al.* (2008) Myrtucommulones F-I, phloroglucinols with thyrotropin-releasing hormone receptor-2 binding affinity from the seeds of Corymbia scabrida. *J Nat Prod*, **71**, 1564-1568.
126. Casero, C., Machin, F., Mendez-Alvarez, S. *et al.* (2015) Structure and antimicrobial activity of phloroglucinol derivatives from Achyrocline satureioides. *J Nat Prod*, **78**, 93-102.
127. Castro-Castillo, V., Rebolledo-Fuentes, M., Theoduloz, C. *et al.* (2010) Synthesis of lakshminine and antiproliferative testing of related oxoisoaporphines. *J Nat Prod*, **73**, 1951-1953.
128. Castro, A., Coll, J. and Arfan, M. (2011) neo-Clerodane diterpenoids from Ajuga bracteosa. *J Nat Prod*, **74**, 1036-1041.
129. Challenger, F. and Greenwood, D. (1949) Sulphur compounds of the genus Allium. Detection of n-propylthiol in the onion. The fission and methylation of diallyl disulphide in cultures of Scopulariopsis brevicaulis. *Biochem J*, **44**, 87-91.

130. Chambers, J.M., Huang, D.C., Lindqvist, L.M. *et al.* (2012) Total synthesis of 2''',5'''-diepisilvestrol and its C1''' epimer: key structure activity relationships at C1''' and C2'''. *J Nat Prod*, **75**, 1500-1504.
131. Chan, P.K., Zhao, M., Che, C.T. *et al.* (2008) Cytotoxic acylated triterpene saponins from the husks of Xanthoceras sorbifolia. *J Nat Prod*, **71**, 1247-1250.
132. Chan, S.C., Chang, Y.S., Wang, J.P. *et al.* (1998) Three new flavonoids and antiallergic, anti-inflammatory constituents from the heartwood of Dalbergia odorifera. *Planta Med*, **64**, 153-158.
133. Chan, S.C., Ko, H.H. and Lin, C.N. (2003) New prenylflavonoids from Artocarpus communis. *J Nat Prod*, **66**, 427-430.
134. Chang, C.C., Ho, S.L. and Lee, S.S. (2015) Acylated glucosylflavones as alpha-glucosidase inhibitors from Tinospora crispa leaf. *Bioorg Med Chem*, **23**, 3388-3396.
135. Chang, F.R. and Wu, Y.C. (2001) Novel cytotoxic annonaceous acetogenins from Annona muricata. *J Nat Prod*, **64**, 925-931.
136. Chang, F.R., Wu, Y.C., Duh, C.Y. *et al.* (1993) Studies on the acetogenins of Formosan annonaceous plants. II. Cytotoxic acetogenins from Annona reticulata. *J Nat Prod*, **56**, 1688-1694.
137. Chang, F.R., Yen, C.T., El-Shazly, M. *et al.* (2013) Spirostanoids with 1,4-dien-3-one or 3beta,7alpha-diol-5,6-ene moieties from Solanum violaceum. *Bioorg Med Chem Lett*, **23**, 2738-2742.
138. Chang, H.M., El-Fishawy, A.M., Slatkin, D.J. *et al.* (1984) Quaternary Alkaloids of Tinospora capillipes. *Planta Med*, **50**, 88-90.
139. Chang, H.T., Cheng, Y.H., Wu, C.L. *et al.* (2008) Antifungal activity of essential oil and its constituents from Calocedrus macrolepis var. formosana Florin leaf against plant pathogenic fungi. *Bioresour Technol*, **99**, 6266-6270.
140. Chang, L.C., Bhat, K.P., Pisha, E. *et al.* (1998) Activity-guided isolation of steroidal alkaloid antiestrogen-binding site inhibitors from Pachysandra procumbens. *J Nat Prod*, **61**, 1257-1262.
141. Chang, X., Li, W., Jia, Z. *et al.* (2007) Biologically active triterpenoid saponins from Ardisia japonica. *J Nat Prod*, **70**, 179-187.
142. Charisiadis, P., Primikyri, A., Exarchou, V. *et al.* (2011) Unprecedented ultra-high-resolution hydroxy group (1)H NMR spectroscopic analysis of plant extracts. *J Nat Prod*, **74**, 2462-2466.
143. Chattopadhyay, S.K., Pal, A., Maulik, P.R. *et al.* (2006) Taxoid from the needles of the Himalayan yew Taxus wallichiana with cytotoxic and immunomodulatory activities. *Bioorg Med Chem Lett*, **16**, 2446-2449.
144. Chaturvedula, V.S., Gao, Z., Jones, S.H. *et al.* (2004) A new ursane triterpene from Monochaetum vulcanicum that inhibits DNA polymerase beta lyase. *J Nat Prod*, **67**, 899-901.
145. Chaturvedula, V.S., Schilling, J.K., Miller, J.S. *et al.* (2004) New cytotoxic terpenoids from the wood of Vepris punctata from the Madagascar Rainforest. *J Nat Prod*, **67**, 895-898.
146. Chaturvedula, V.S., Schilling, J.K., Miller, J.S. *et al.* (2002) Two new triterpene esters from the twigs of Brachylaena ramiflora from the Madagascar rainforest. *J Nat Prod*, **65**, 1222-1224.
147. Chavan, M.J., Wakte, P.S. and Shinde, D.B. (2009) Saturated long-chain hydrocarbons from Annona squamosa L. bark. *Nat Prod Res*, **23**, 455-459.
148. Chawla, A.S., Sharma, A.K., Handa, S.S. *et al.* (1992) Chemical investigation and anti-inflammatory activity of Vitex negundo seeds. *J Nat Prod*, **55**, 163-167.
149. Cheenpracha, S., Karalai, C., Ponglimanont, C. *et al.* (2009) Candenatenins A-F, phenolic compounds from the heartwood of Dalbergia candenatensis. *J Nat Prod*, **72**, 1395-1398.
150. Chen, C., Qiang, S., Lou, L. *et al.* (2009) Cucurbitane-type triterpenoids from the stems of Cucumis melo. *J Nat Prod*, **72**, 824-829.
151. Chen, C.C., Hsin, W.C., Ko, F.N. *et al.* (1996) Antiplatelet arylnaphthalide lignans from Justicia procumbens. *J Nat Prod*, **59**, 1149-1150.
152. Chen, C.C., Huang, Y.L., Sun, C.M. *et al.* (1996) New prenylflavones from the leaves of Epimedium saggitatum. *J Nat Prod*, **59**, 412-414.
153. Chen, C.H., Lin, J.Y., Lin, C.N. *et al.* (1992) Inhibition of angiotensin-I-converting enzyme by tetrahydroxyxanthones isolated from Tripterospermum lanceolatum. *J Nat Prod*, **55**, 691-695.
154. Chen, C.H., Lo, W.L., Liu, Y.C. *et al.* (2006) Chemical and cytotoxic constituents from the leaves of Cinnamomum kotoense. *J Nat Prod*, **69**, 927-933.
155. Chen, C.Y., Chang, F.R., Shih, Y.C. *et al.* (2000) Cytotoxic constituents of Polyalthia longifolia var. pendula. *J Nat Prod*, **63**, 1475-1478.
156. Chen, C.Y., Chen, C.H., Wong, C.H. *et al.* (2007) Cytotoxic constituents of the stems of Cinnamomum subavenium. *J Nat Prod*, **70**, 103-106.
157. Chen, D.F., Zhang, S.X., Chen, K. *et al.* (1996) Two new lignans, interiotherins A and B, as anti-HIV principles from Kadsura interior. *J Nat Prod*, **59**, 1066-1068.

158. Chen, D.F., Zhang, S.X., Kozuka, M. *et al.* (2002) Interiotherins C and D, two new lignans from Kadsura interior and antitumor-promoting effects of related neolignans on Epstein-Barr virus activation. *J Nat Prod*, **65**, 1242-1245.
159. Chen, F.C., Peng, C.F., Tsai, I.L. *et al.* (2005) Antitubercular constituents from the stem wood of Cinnamomum kotoense. *J Nat Prod*, **68**, 1318-1323.
160. Chen, H., Bai, J., Fang, Z.F. *et al.* (2013) [Chemical constituents from stems of Brucea mollis and their cytotoxic activity]. *Zhongguo Zhong Yao Za Zhi*, **38**, 2321-2324.
161. Chen, H., Bai, J., Fang, Z.F. *et al.* (2011) Indole alkaloids and quassinoids from the stems of Brucea mollis. *J Nat Prod*, **74**, 2438-2445.
162. Chen, H.W., Lin, A.H., Chu, H.C. *et al.* (2011) Inhibition of TNF-alpha-Induced Inflammation by andrographolide via down-regulation of the PI3K/Akt signaling pathway. *J Nat Prod*, **74**, 2408-2413.
163. Chen, I.H., Chang, F.R., Wu, C.C. *et al.* (2006) Cytotoxic triterpenoids from the leaves of Microtropis fokienensis. *J Nat Prod*, **69**, 1543-1546.
164. Chen, I.H., Du, Y.C., Lu, M.C. *et al.* (2008) Lupane-type triterpenoids from Microtropis fokienensis and Perrottetia arisanensis and the apoptotic effect of 28-hydroxy-3-oxo-lup-20(29)-en-30-al. *J Nat Prod*, **71**, 1352-1357.
165. Chen, I.S., Chen, H.F., Cheng, M.J. *et al.* (2001) Quinoline alkaloids and other constituents of Melicope semecarpifolia with antiplatelet aggregation activity. *J Nat Prod*, **64**, 1143-1147.
166. Chen, I.S., Chen, T.L., Chang, Y.L. *et al.* (1999) Chemical constituents and biological activities of the fruit of Zanthoxylum integrifoliolum. *J Nat Prod*, **62**, 833-837.
167. Chen, I.S., Wu, S.J. and Tsai, I.L. (1994) Chemical and bioactive constituents from Zanthoxylum simulans. *J Nat Prod*, **57**, 1206-1211.
168. Chen, J., Yan, X.H., Dong, J.H. *et al.* (2009) Tobacco mosaic virus (TMV) inhibitors from Picrasma quassioides Benn. *J Agric Food Chem*, **57**, 6590-6595.
169. Chen, J.J., Chen, C.J., Yao, X.J. *et al.* (2014) Eremophilane-type sesquiterpenoids with diverse skeletons from Ligularia sagitta. *J Nat Prod*, **77**, 1329-1335.
170. Chen, J.J., Chen, P.H., Liao, C.H. *et al.* (2007) New phenylpropenoids, bis(1-phenylethyl)phenols, bisquinolinone alkaloid, and anti-inflammatory constituents from Zanthoxylum integrifoliolum. *J Nat Prod*, **70**, 1444-1448.
171. Chen, J.J., Cho, J.Y., Hwang, T.L. *et al.* (2008) Benzoic acid derivatives, acetophenones, and anti-inflammatory constituents from Melicope semecarpifolia. *J Nat Prod*, **71**, 71-75.
172. Chen, J.J., Chou, T.H., Duh, C.Y. *et al.* (2006) Cytotoxic dihydroagarofuranoid sesquiterpenes from the stem of Microtropis fokienensis. *J Nat Prod*, **69**, 685-688.
173. Chen, J.J., Chou, T.H., Peng, C.F. *et al.* (2007) Antitubercular dihydroagarofuranoid sesquiterpenes from the roots of Microtropis fokienensis. *J Nat Prod*, **70**, 202-205.
174. Chen, J.J., Chung, C.Y., Hwang, T.L. *et al.* (2009) Amides and benzenoids from Zanthoxylum ailanthoides with inhibitory activity on superoxide generation and elastase release by neutrophils. *J Nat Prod*, **72**, 107-111.
175. Chen, J.J., Li, W.X., Gao, K. *et al.* (2012) Absolute structures of monoterpenoids with a delta-lactone-containing skeleton from Ligularia hodgsonii. *J Nat Prod*, **75**, 1184-1188.
176. Chen, J.J., Tsai, Y.C., Hwang, T.L. *et al.* (2011) Thymol, benzofuranoid, and phenylpropanoid derivatives: anti-inflammatory constituents from Eupatorium cannabinum. *J Nat Prod*, **74**, 1021-1027.
177. Chen, J.J., Wang, T.Y. and Hwang, T.L. (2008) Neolignans, a coumarinolignan, lignan derivatives, and a chromene: anti-inflammatory constituents from Zanthoxylum avicennae. *J Nat Prod*, **71**, 212-217.
178. Chen, J.J., Wu, H.M., Peng, C.F. *et al.* (2009) seco-Abietane diterpenoids, a phenylethanoid derivative, and antitubercular constituents from Callicarpa pilosissima. *J Nat Prod*, **72**, 223-228.
179. Chen, J.J., Yang, C.S., Peng, C.F. *et al.* (2008) Dihydroagarofuranoid sesquiterpenes, a lignan derivative, a benzenoid, and antitubercular constituents from the stem of Microtropis japonica. *J Nat Prod*, **71**, 1016-1021.
180. Chen, J.L., Gerwick, W.H., Schatzman, R. *et al.* (1994) Isorawsonol and related IMP dehydrogenase inhibitors from the tropical green alga Avrainvillea rawsonii. *J Nat Prod*, **57**, 947-952.
181. Chen, K., Shi, Q., Kashiwada, Y. *et al.* (1992) Anti-aids agents, 6. Salaspermic acid, an anti-HIV principle from Tripterygium wilfordii, and the structure-activity correlation with its related compounds. *J Nat Prod*, **55**, 340-346.
182. Chen, L., Liang, Y., Song, T. *et al.* (2015) Synthesis and bioactivity of tripolinolate A from Tripolium vulgare and its analogs. *Bioorg Med Chem Lett*, **25**, 2629-2633.
183. Chen, L., Zhu, H., Wang, R. *et al.* (2008) ent-Labdane diterpenoid lactone stereoisomers from Andrographis paniculata. *J Nat Prod*, **71**, 852-855.
184. Chen, M., Gan, L., Lin, S. *et al.* (2012) Alkaloids from the root of Isatis indigotica. *J Nat Prod*, **75**, 1167-1176.

185. Chen, M., Kilgore, N., Lee, K.H. *et al.* (2006) Rubrisandrins A and B, lignans and related anti-HIV compounds from Schisandra rubriflora. *J Nat Prod*, **69**, 1697-1701.
186. Chen, Q.B., Xin, X.L., Yang, Y. *et al.* (2014) Highly conjugated norditerpenoid and pyrroloquinoline alkaloids with potent PTP1B inhibitory activity from Nigella glandulifera. *J Nat Prod*, **77**, 807-812.
187. Chen, Q.C., Youn, U., Min, B.S. *et al.* (2008) Pyronane monoterpenoids from the fruit of Gardenia jasminoides. *J Nat Prod*, **71**, 995-999.
188. Chen, Q.L., Shi, Z.Y., Tu, G.Z. *et al.* (2005) [Studies on the chemical constituents in root of Gentiana macrophylla from Shaanxi]. *Zhongguo Zhong Yao Za Zhi*, **30**, 1519-1522.
189. Chen, Q.L., Shi, Z.Y., Zhang, Y.H. *et al.* (2011) [Study on the chemical constituents in roots of Gentiana dahurica]. *Zhong Yao Cai*, **34**, 1214-1216.
190. Chen, R.M., Hu, L.H., An, T.Y. *et al.* (2002) Natural PTP1B inhibitors from Broussonetia papyrifera. *Bioorg Med Chem Lett*, **12**, 3387-3390.
191. Chen, S.N., Friesen, J.B., Webster, D. *et al.* (2011) Phytoconstituents from Vitex agnus-castus fruits. *Fitoterapia*, **82**, 528-533.
192. Chen, W.H., Wang, R. and Shi, Y.P. (2010) Flavonoids in the poisonous plant Oxytropis falcata. *J Nat Prod*, **73**, 1398-1403.
193. Chen, X.Y., Wang, H.Q., Zhang, T. *et al.* (2013) Aromatic glucosides from the seeds of Prunus davidiana. *J Nat Prod*, **76**, 1528-1534.
194. Chen, Y., Chen, J.W. and Li, X. (2011) Cytotoxic bistetrahydrofuran annonaceous acetogenins from the seeds of Annona squamosa. *J Nat Prod*, **74**, 2477-2481.
195. Chen, Y. and Dai, G. (2015) Acaricidal activity of compounds from Cinnamomum camphora (L.) Presl against the carmine spider mite, Tetranychus cinnabarinus. *Pest Manag Sci*, **71**, 1561-1571.
196. Chen, Y., Fu, T., Tao, T. *et al.* (2005) Macrophage activating effects of new alkamides from the roots of Echinacea species. *J Nat Prod*, **68**, 773-776.
197. Chen, Y.C., Liaw, C.C., Cheng, Y.B. *et al.* (2013) Anti-liver fibrotic lignans from the fruits of Schisandra arisanensis and Schisandra sphenanthera. *Bioorg Med Chem Lett*, **23**, 880-885.
198. Chen, Y.L., Lan, Y.H., Hsieh, P.W. *et al.* (2008) Bioactive cembrane diterpenoids of Anisomeles indica. *J Nat Prod*, **71**, 1207-1212.
199. Chen, Y.S., Yu, H.M., Shie, J.J. *et al.* (2014) Chemical constituents of Plectranthus amboinicus and the synthetic analogs possessing anti-inflammatory activity. *Bioorg Med Chem*, **22**, 1766-1772.
200. Chen, Z., Liu, Y.M., Yang, S. *et al.* (2008) Studies on the chemical constituents and anticancer activity of Saxifraga stolonifera (L) Meeb. *Bioorg Med Chem*, **16**, 1337-1344.
201. Chen, Z., Wang, S., Zeng, K.W. *et al.* (2014) Rupestonic acids B-G, NO inhibitory sesquiterpenoids from Artemisia rupestris. *Bioorg Med Chem Lett*, **24**, 4318-4322.
202. Cheng, H.H., Wang, H.K., Ito, J. *et al.* (2001) Cytotoxic pheophorbide-related compounds from Clerodendrum calamitosum and C. cyrtophyllum. *J Nat Prod*, **64**, 915-919.
203. Cheng, J.J., Zhang, L.J., Cheng, H.L. *et al.* (2010) Cytotoxic Hexacyclic Triterpene Acids from Euscaphis japonica. *J Nat Prod*, **73**, 1655-1658.
204. Cheng, M.C., Li, C.Y., Ko, H.C. *et al.* (2006) Antidepressant principles of the roots of Polygala tenuifolia. *J Nat Prod*, **69**, 1305-1309.
205. Cheng, Y.B., Chang, M.T., Lo, Y.W. *et al.* (2009) Oxygenated lignans from the fruits of Schisandra arisanensis. *J Nat Prod*, **72**, 1663-1668.
206. Cheng, Y.B., Liao, T.C., Lo, Y.W. *et al.* (2010) Nortriterpene lactones from the fruits of Schisandra arisanensis. *J Nat Prod*, **73**, 1228-1233.
207. Chernetsova, E.S., Shikov, A.N., Crawford, E.A. *et al.* (2014) Characterization of volatile and semi-volatile compounds in green and fermented leaves of Bergenia crassifolia L. by gas chromatography-mass spectrometry and ID-CUBE direct analysis in real time-high resolution mass spectrometry. *Eur J Mass Spectrom (Chichester)*, **20**, 199-205.
208. Chia, Y.C., Chang, F.R., Teng, C.M. *et al.* (2000) Aristolactams and dioxoaporphines from Fissistigma balansae and Fissistigma oldhamii. *J Nat Prod*, **63**, 1160-1163.
209. Chianese, G., Yerbanga, S.R., Lucantoni, L. *et al.* (2010) Antiplasmodial triterpenoids from the fruits of neem, Azadirachta indica. *J Nat Prod*, **73**, 1448-1452.
210. Chiang, Y.M., Kuo, Y.H., Oota, S. *et al.* (2003) Xanthones and benzophenones from the stems of Garcinia multiflora. *J Nat Prod*, **66**, 1070-1073.
211. Chiang, Y.M., Lo, C.P., Chen, Y.P. *et al.* (2005) Ethyl caffeate suppresses NF-kappaB activation and its downstream inflammatory mediators, iNOS, COX-2, and PGE2 in vitro or in mouse skin. *Br J Pharmacol*, **146**, 352-363.
212. Chiari, M.E., Vera, D.M., Palacios, S.M. *et al.* (2011) Tyrosinase inhibitory activity of a 6-isoprenoid-substituted flavanone isolated from Dalea elegans. *Bioorg Med Chem*, **19**, 3474-3482.

213. Chin, Y.W., Lim, S.W., Kim, S.H. *et al.* (2003) Hepatoprotective pyrrole derivatives of Lycium chinense fruits. *Bioorg Med Chem Lett*, **13**, 79-81.
214. Chintalwar, G.J. and Chattopadhyay, S. (2006) Structural confirmation of decussatin, a Swertia decussata xanthone. *Nat Prod Res*, **20**, 53-56.
215. Cho, J.K., Curtis-Long, M.J., Lee, K.H. *et al.* (2013) Geranylated flavonoids displaying SARS-CoV papain-like protease inhibition from the fruits of Paulownia tomentosa. *Bioorg Med Chem*, **21**, 3051-3057.
216. Cho, J.K., Ryu, Y.B., Curtis-Long, M.J. *et al.* (2012) Cholinestrase inhibitory effects of geranylated flavonoids from Paulownia tomentosa fruits. *Bioorg Med Chem*, **20**, 2595-2602.
217. Cho, J.Y., Baik, K.U., Yoo, E.S. *et al.* (2000) In vitro antiinflammatory effects of neolignan woorenosides from the rhizomes of Coptis japonica. *J Nat Prod*, **63**, 1205-1209.
218. Choi, H.J., Lee, J.H. and Jung, Y.S. (2014) (+)-Nootkatone inhibits tumor necrosis factor alpha/interferon gamma-induced production of chemokines in HaCaT cells. *Biochem Biophys Res Commun*, **447**, 278-284.
219. Choi, J.H., Kim, D.W., Yun, N. *et al.* (2011) Protective effects of hyperoside against carbon tetrachloride-induced liver damage in mice. *J Nat Prod*, **74**, 1055-1060.
220. Choi, J.S., Yokozawa, T. and Oura, H. (1991) Antihyperlipidemic effect of flavonoids from Prunus davidiana. *J Nat Prod*, **54**, 218-224.
221. Choi, S.Z., Kwon, H.C., Choi, S.U. *et al.* (2002) Five new labdane diterpenes from Aster oharai. *J Nat Prod*, **65**, 1102-1106.
222. Choi, Y.H., Hussain, R.A., Pezzuto, J.M. *et al.* (1989) Abrusosides A-D, four novel sweet-tasting triterpene glycosides from the leaves of Abrus precatorius. *J Nat Prod*, **52**, 1118-1127.
223. Choi, Y.H., Zhou, W., Oh, J. *et al.* (2012) Rhododendric acid A, a new ursane-type PTP1B inhibitor from the endangered plant Rhododendron brachycarpum G. Don. *Bioorg Med Chem Lett*, **22**, 6116-6119.
224. Chou, T.H., Chen, J.J., Lee, S.J. *et al.* (2010) Cytotoxic flavonoids from the leaves of Cryptocarya chinensis. *J Nat Prod*, **73**, 1470-1475.
225. Chou, T.H., Ding, H.Y., Lin, R.J. *et al.* (2010) Inhibition of melanogenesis and oxidation by protocatechuic acid from Origanum vulgare (oregano). *J Nat Prod*, **73**, 1767-1774.
226. Choudhary, M.I., Ismail, M., Shaari, K. *et al.* (2010) cis-Clerodane-type furanoditerpenoids from Tinospora crispa. *J Nat Prod*, **73**, 541-547.
227. Choudhary, M.I., Jan, S., Abbaskhan, A. *et al.* (2008) Cycloartane triterpenoids from Astragalus bicuspis. *J Nat Prod*, **71**, 1557-1560.
228. Chumkaew, P., Karalai, C., Ponglimanont, C. *et al.* (2003) Antimycobacterial activity of phorbol esters from the fruits of Sapium indicum. *J Nat Prod*, **66**, 540-543.
229. Chung, I.M., Ahmad, A., Ali, M. *et al.* (2009) Flavonoid glucosides from the hairy roots of Catharanthus roseus. *J Nat Prod*, **72**, 613-620.
230. Chung, M.I., Gan, K.H., Lin, C.N. *et al.* (1993) Antiplatelet effects and vasorelaxing action of some constituents of Formosan plants. *J Nat Prod*, **56**, 929-934.
231. Chung, M.I., Jou, S.J., Cheng, T.H. *et al.* (1994) Antiplatelet constituents of formosan Rubia akane. *J Nat Prod*, **57**, 313-316.
232. Chung, Y.M., Chang, F.R., Tseng, T.F. *et al.* (2011) A novel alkaloid, aristopyridinone A and anti-inflammatory phenanthrenes isolated from Aristolochia manshuriensis. *Bioorg Med Chem Lett*, **21**, 1792-1794.
233. Cimanga, K., De Bruyne, T., Lasure, A. *et al.* (1995) In vitro anticomplementary activity of constituents from Morinda morindoides. *J Nat Prod*, **58**, 372-378.
234. Cimanga, K., Hermans, N., Apers, S. *et al.* (2003) Complement-inhibiting iridoids from Morinda morindoides. *J Nat Prod*, **66**, 97-102.
235. Cioffi, G., Dal Piaz, F., De Caprariis, P. *et al.* (2006) Antiproliferative triterpene saponins from Entada africana. *J Nat Prod*, **69**, 1323-1329.
236. Cioffi, G., Dal Piaz, F., Vassallo, A. *et al.* (2008) Antiproliferative oleanane saponins from Meryta denhamii. *J Nat Prod*, **71**, 1000-1004.
237. Cioffi, G., Morales Escobar, L., Braca, A. *et al.* (2003) Antioxidant chalcone glycosides and flavanones from Maclura (Chlorophora) tinctoria. *J Nat Prod*, **66**, 1061-1064.
238. Citova, I., Ganzera, M., Stuppner, H. *et al.* (2008) Determination of gentisin, isogentisin, and amarogentin in Gentiana lutea L. by capillary electrophoresis. *J Sep Sci*, **31**, 195-200.
239. Collins, D.O., Gallimore, W.A., Reynolds, W.F. *et al.* (2000) New skeletal sesquiterpenoids, caprariolides A-D, from Capraria biflora and their insecticidal activity. *J Nat Prod*, **63**, 1515-1518.
240. Colon, M., Guevara, P., Gerwick, W.H. *et al.* (1987) 5'-Hydroxyisoavrainvilleol, a new diphenylmethane derivative from the tropical green alga Avrainvillea nigricans. *J Nat Prod*, **50**, 368-374.

241. Cong, H.J., Zhang, S.W., Shen, Y. *et al.* (2013) Guanidine alkaloids from Plumbago zeylanica. *J Nat Prod*, **76**, 1351-1357.
242. Costa, S.M., Lemos, T.L., Pessoa, O.D. *et al.* (2001) Chemical constituents from Lippia sidoides and cytotoxic activity. *J Nat Prod*, **64**, 792-795.
243. Cottet, K., Neudorffer, A., Kritsanida, M. *et al.* (2015) Polycyclic Polyprenylated Xanthones from Symphonia globulifera: Isolation and Biomimetic Electrosynthesis. *J Nat Prod*, **78**, 2136-2140.
244. Cui, B., Chai, H., Santisuk, T. *et al.* (1998) Novel cytotoxic acylated oligorhamnosides from Mezzettia leptopoda. *J Nat Prod*, **61**, 1535-1538.
245. Cui, H., Xu, B., Wu, T. *et al.* (2014) Potential antiviral lignans from the roots of Saururus chinensis with activity against Epstein-Barr virus lytic replication. *J Nat Prod*, **77**, 100-110.
246. Cui, L., Ndinteh, D.T., Na, M. *et al.* (2007) Isoprenylated flavonoids from the stem bark of Erythrina abyssinica. *J Nat Prod*, **70**, 1039-1042.
247. Cui, L., Thuong, P.T., Lee, H.S. *et al.* (2008) Flavanones from the stem bark of Erythrina abyssinica. *Bioorg Med Chem*, **16**, 10356-10362.
248. Cuong, N.X., Minh, C.V., Kiem, P.V. *et al.* (2009) Inhibitors of osteoclast formation from rhizomes of Cibotium barometz. *J Nat Prod*, **72**, 1673-1677.
249. Cuong, N.X., Nhiem, N.X., Thao, N.P. *et al.* (2010) Inhibitors of osteoclastogenesis from Lawsonia inermis leaves. *Bioorg Med Chem Lett*, **20**, 4782-4784.
250. Cuong, T.D., Hung, T.M., Kim, J.C. *et al.* (2012) Phenolic compounds from Caesalpinia sappan heartwood and their anti-inflammatory activity. *J Nat Prod*, **75**, 2069-2075.
251. Cuong, T.D., Hung, T.M., Lee, J.S. *et al.* (2015) Anti-inflammatory activity of phenolic compounds from the whole plant of Scutellaria indica. *Bioorg Med Chem Lett*, **25**, 1129-1134.
252. D'Abrosca, B., Buommino, E., D'Angelo, G. *et al.* (2013) Spectroscopic identification and anti-biofilm properties of polar metabolites from the medicinal plant Helichrysum italicum against Pseudomonas aeruginosa. *Bioorg Med Chem*, **21**, 7038-7046.
253. Dai, S.J., Chen, M., Liu, K. *et al.* (2006) Four new neo-clerodane diterpenoid alkaloids from Scutellaria barbata with cytotoxic activities. *Chem Pharm Bull (Tokyo)*, **54**, 869-872.
254. Dai, S.J., Liang, D.D., Ren, Y. *et al.* (2008) New neo-clerodane diterpenoid alkaloids from Scutellaria barbata with cytotoxic activities. *Chem Pharm Bull (Tokyo)*, **56**, 207-209.
255. Dai, S.J., Peng, W.B., Zhang, D.W. *et al.* (2009) Cytotoxic neo-clerodane diterpenoid alkaloids from Scutellaria barbata. *J Nat Prod*, **72**, 1793-1797.
256. Dai, Y., Harinantenaina, L., Bowman, J.D. *et al.* (2014) Isolation of antiplasmodial anthraquinones from Kniphofia ensifolia, and synthesis and structure-activity relationships of related compounds. *Bioorg Med Chem*, **22**, 269-276.
257. Damu, A.G., Kuo, P.C., Shi, L.S. *et al.* (2005) Phenanthroindolizidine alkaloids from the stems of Ficus septica. *J Nat Prod*, **68**, 1071-1075.
258. Dang, Q.L., Kim, W.K., Nguyen, C.M. *et al.* (2011) Nematicidal and antifungal activities of annonaceous acetogenins from Annona squamosa against various plant pathogens. *J Agric Food Chem*, **59**, 11160-11167.
259. Dao, T.T., Tran, T.L., Kim, J. *et al.* (2012) Terpenylated coumarins as SIRT1 activators isolated from Ailanthus altissima. *J Nat Prod*, **75**, 1332-1338.
260. Das Sarma, M., Ghosh, R., Patra, A. *et al.* (2007) Synthesis and antiproliferative activity of some novel derivatives of diospyrin, a plant-derived naphthoquinonoid. *Bioorg Med Chem*, **15**, 3672-3677.
261. Dat le, D., Thao, N.P., Tai, B.H. *et al.* (2015) Chemical constituents from Kandelia candel with their inhibitory effects on pro-inflammatory cytokines production in LPS-stimulated bone marrow-derived dendritic cells (BMDCs). *Bioorg Med Chem Lett*, **25**, 1412-1416.
262. Dat, N.T., Bae, K., Wamiru, A. *et al.* (2007) A dimeric lactone from Ardisia japonica with inhibitory activity for HIV-1 and HIV-2 ribonuclease H. *J Nat Prod*, **70**, 839-841.
263. Dat, N.T., Jin, X., Lee, J.H. *et al.* (2007) Abietane diterpenes from Salvia miltiorrhiza inhibit the activation of hypoxia-inducible factor-1. *J Nat Prod*, **70**, 1093-1097.
264. Dat, N.T., Lee, J.H., Lee, K. *et al.* (2008) Phenolic constituents of Amorpha fruticosa that inhibit NF-kappaB activation and related gene expression. *J Nat Prod*, **71**, 1696-1700.
265. Davenport, J., Manjarrez, J.R., Peterson, L. *et al.* (2011) Gambogic acid, a natural product inhibitor of Hsp90. *J Nat Prod*, **74**, 1085-1092.
266. Davis, R.A., Barnes, E.C., Longden, J. *et al.* (2009) Isolation, structure elucidation and cytotoxic evaluation of endiandrin B from the Australian rainforest plant Endiandra anthropophagorum. *Bioorg Med Chem*, **17**, 1387-1392.
267. Davis, R.A., Carroll, A.R., Duffy, S. *et al.* (2007) Endiandrin A, a potent glucocorticoid receptor binder isolated from the Australian plant Endiandra anthropophagorum. *J Nat Prod*, **70**, 1118-1121.

268. Day, S.H., Chiu, N.Y., Tsao, L.T. *et al.* (2000) New lignan glycosides with potent antiinflammatory effect, isolated from Justicia ciliata. *J Nat Prod*, **63**, 1560-1562.
269. Day, S.H., Chiu, N.Y., Won, S.J. *et al.* (1999) Cytotoxic lignans of Justicia ciliata. *J Nat Prod*, **62**, 1056-1058.
270. Day, S.H., Lin, Y.C., Tsai, M.L. *et al.* (2002) Potent cytotoxic lignans from Justicia procumbens and their effects on nitric oxide and tumor necrosis factor-alpha production in mouse macrophages. *J Nat Prod*, **65**, 379-381.
271. de Lima, F.O., Nonato, F.R., Couto, R.D. *et al.* (2011) Mechanisms involved in the antinociceptive effects of 7-hydroxycoumarin. *J Nat Prod*, **74**, 596-602.
272. de Sousa, L.R., Ramalho, S.D., Burger, M.C. *et al.* (2014) Isolation of arginase inhibitors from the bioactivity-guided fractionation of Byrsonima coccolobifolia leaves and stems. *J Nat Prod*, **77**, 392-396.
273. Delgado, G., del Socorro Olivares, M., Chavez, M.I. *et al.* (2001) Antiinflammatory constituents from Heterotheca inuloides. *J Nat Prod*, **64**, 861-864.
274. DellaGreca, M., Cutillo, F., D'Abrosca, B. *et al.* (2009) Antioxidant and radical scavenging properties of Malva sylvestris. *Nat Prod Commun*, **4**, 893-896.
275. DellaGreca, M., Di Marino, C., Zarrelli, A. *et al.* (2004) Isolation and phytotoxicity of apocarotenoids from Chenopodium album. *J Nat Prod*, **67**, 1492-1495.
276. Deng, J.Z., Starck, S.R. and Hecht, S.M. (1999) bis-5-Alkylresorcinols from Panopsis rubescens that inhibit DNA polymerase beta. *J Nat Prod*, **62**, 477-480.
277. Deng, S., Chen, S.N., Yao, P. *et al.* (2006) Serotonergic activity-guided phytochemical investigation of the roots of Angelica sinensis. *J Nat Prod*, **69**, 536-541.
278. Deng, S., Palu, K., West, B.J. *et al.* (2007) Lipoxygenase inhibitory constituents of the fruits of noni (Morinda citrifolia) collected in Tahiti. *J Nat Prod*, **70**, 859-862.
279. Deng, Y., Balunas, M.J., Kim, J.A. *et al.* (2009) Bioactive 5,6-dihydro-alpha-pyrone derivatives from Hyptis brevipes. *J Nat Prod*, **72**, 1165-1169.
280. Deng, Y., Chin, Y.W., Chai, H. *et al.* (2007) Anthraquinones with quinone reductase-inducing activity and benzophenones from Morinda citrifolia (noni) roots. *J Nat Prod*, **70**, 2049-2052.
281. Devkota, K.P., Lenta, B.N., Choudhary, M.I. *et al.* (2007) Cholinesterase inhibiting and antiplasmodial steroidal alkaloids from Sarcococca hookeriana. *Chem Pharm Bull (Tokyo)*, **55**, 1397-1401.
282. Devkota, K.P., Lenta, B.N., Wansi, J.D. *et al.* (2008) Bioactive 5alpha-pregnane-type steroidal alkaloids from Sarcococca hookeriana. *J Nat Prod*, **71**, 1481-1484.
283. Dharmaratne, H.R., Nanayakkara, N.P. and Khan, I.A. (2002) Kavalactones from Piper methysticum, and their 13C NMR spectroscopic analyses. *Phytochemistry*, **59**, 429-433.
284. Diaz Chavez, M.L., Rolf, M., Gesell, A. *et al.* (2011) Characterization of two methylenedioxy bridge-forming cytochrome P450-dependent enzymes of alkaloid formation in the Mexican prickly poppy Argemone mexicana. *Arch Biochem Biophys*, **507**, 186-193.
285. Diaz, F., Chavez, D., Lee, D. *et al.* (2003) Cytotoxic flavone analogues of vitexicarpin, a constituent of the leaves of Vitex negundo. *J Nat Prod*, **66**, 865-867.
286. Diaz, J.G., Ruiz, J.G. and de La Fuente, G. (2000) Alkaloids from Delphinium staphisagria. *J Nat Prod*, **63**, 1136-1139.
287. Ding, L.S., Chen, Y.Z. and Wu, F.E. (1991) Diterpenoid Alkaloids from Aconitum vilmorrianum. *Planta Med*, **57**, 275-277.
288. Ding, W., Zeng, F., Xu, L. *et al.* (2011) Bioactive dammarane-type saponins from Operculina turpethum. *J Nat Prod*, **74**, 1868-1874.
289. Ding, X.F., Feng, X., Dong, Y.F. *et al.* (2008) [Studies on chemical constituents of the roots of Angelica pubescens]. *Zhong Yao Cai*, **31**, 516-518.
290. Ding, Y., Su, Y., Guo, H. *et al.* (2010) Phenylpropanoyl esters from Horseweed (Conyza canadensis) and their inhibitory effects on catecholamine secretion. *J Nat Prod*, **73**, 270-274.
291. Dirican, E. and Turkez, H. (2014) In vitro studies on protective effect of Glycyrrhiza glabra root extracts against cadmium-induced genetic and oxidative damage in human lymphocytes. *Cytotechnology*, **66**, 9-16.
292. Dixit, P., Chillara, R., Khedgikar, V. *et al.* (2012) Constituents of Dalbergia sissoo Roxb. leaves with osteogenic activity. *Bioorg Med Chem Lett*, **22**, 890-897.
293. Doebbe, A., Keck, M., La Russa, M. *et al.* (2010) The interplay of proton, electron, and metabolite supply for photosynthetic H2 production in Chlamydomonas reinhardtii. *J Biol Chem*, **285**, 30247-30260.
294. Don, M.J., Shen, C.C., Lin, Y.L. *et al.* (2005) Nitrogen-containing compounds from Salvia miltiorrhiza. *J Nat Prod*, **68**, 1066-1070.
295. Donega, M.A., Mello, S.C., Moraes, R.M. *et al.* (2014) Pharmacological activities of cilantro's aliphatic aldehydes against Leishmania donovani. *Planta Med*, **80**, 1706-1711.

296. Dong, L.B., He, J., Wang, Y.Y. *et al.* (2011) Terpenoids and norlignans from Metasequoia glyptostroboides. *J Nat Prod*, **74**, 234-239.
297. Dong, S.H., Zhang, C.R., Xu, C.H. *et al.* (2011) Daphnane-type diterpenoids from Trigonostemon howii. *J Nat Prod*, **74**, 1255-1261.
298. Donoso-Fierro, C., Tiezzi, A., Ovidi, E. *et al.* (2015) Antiproliferative activity of yatein isolated from Austrocedrus chilensis against murine myeloma cells: cytological studies and chemical investigations. *Pharm Biol*, **53**, 378-385.
299. Duan, H., Takaishi, Y., Imakura, Y. *et al.* (2000) Sesquiterpene alkaloids from Tripterygium hypoglaucum and Tripterygium wilfordii: a new class of potent anti-HIV agents. *J Nat Prod*, **63**, 357-361.
300. Duan, H., Takaishi, Y., Momota, H. *et al.* (1999) Immunosuppressive diterpenoids from Tripterygium wilfordii. *J Nat Prod*, **62**, 1522-1525.
301. Duan, H., Takaishi, Y., Momota, H. *et al.* (2001) Immunosuppressive sesquiterpene alkaloids from Tripterygium wilfordii. *J Nat Prod*, **64**, 582-587.
302. Duelund, L., Amiot, A., Fillon, A. *et al.* (2012) Influence of the active compounds of Perilla frutescens leaves on lipid membranes. *J Nat Prod*, **75**, 160-166.
303. Duh, C.Y., Pezzuto, J.M., Kinghorn, A.D. *et al.* (1987) Plant anticancer agents XLIV. Cytotoxic constituents from Stizophyllum riparium. *J Nat Prod*, **50**, 63-74.
304. Ee, G.C., Daud, S., Izzaddin, S.A. *et al.* (2008) Garcinia mangostana: a source of potential anti-cancer lead compounds against CEM-SS cell line. *J Asian Nat Prod Res*, **10**, 475-479.
305. Efdi, M., Ninomiya, M., Suryani, E. *et al.* (2012) Sentulic acid: a cytotoxic ring A-seco triterpenoid from Sandoricum koetjape Merr. *Bioorg Med Chem Lett*, **22**, 4242-4245.
306. Egger, K. and Keil, M. (1969) [Flavoneglycosides in the flowers of Paeonia arborea and P. suffruticosa]. *Planta*, **88**, 154-156.
307. El-Ghorab, A.H., Nauman, M., Anjum, F.M. *et al.* (2010) A comparative study on chemical composition and antioxidant activity of ginger (Zingiber officinale) and cumin (Cuminum cyminum). *J Agric Food Chem*, **58**, 8231-8237.
308. El-Sayed, A., Handy, G.A. and Cordell, G.A. (1983) Catharanthus alkaloids, XXXVIII. Confirming structural evidence and antineoplastic activity of the bisindole alkaloids leurosine-N'b-oxide (pleurosine), roseadine and vindolicine from Catharanthus roseus. *J Nat Prod*, **46**, 517-527.
309. Engelmeier, D., Hadacek, F., Hofer, O. *et al.* (2004) Antifungal 3-butylisocoumarins from Asteraceae-Anthemideae. *J Nat Prod*, **67**, 19-25.
310. Eparvier, V., Nguyen, V.H., Thoison, O. *et al.* (2006) Cytotoxic monotetrahydrofuran acetogenins from Disepalum plagioneurum. *J Nat Prod*, **69**, 1289-1294.
311. Epifano, F., Genovese, S., James Squires, E. *et al.* (2012) Nelumal A, the active principle from Ligularia nelumbifolia, is a novel farnesoid X receptor agonist. *Bioorg Med Chem Lett*, **22**, 3130-3135.
312. Ernstsen, A., Sandberg, G. and Lundstrom, K. (1987) Identification of oxindole-3-acetic acid, and metabolic conversion of indole-3-acetic acid to oxindole-3-acetic acid in Pinus sylvestris seeds. *Planta*, **172**, 47-52.
313. Es-Safi, N.E., Khlifi, S., Kerhoas, L. *et al.* (2005) Antioxidant constituents of the aerial parts of Globularia alypum growing in Morocco. *J Nat Prod*, **68**, 1293-1296.
314. Estevez-Braun, A., Estevez-Reyes, R., Moujir, L.M. *et al.* (1994) Antibiotic activity and absolute configuation of 8S-heptadeca-2(Z),9(Z)-diene-4,6-diyne-1,8-diol from Bupleurum salicifolium. *J Nat Prod*, **57**, 1178-1182.
315. Falcao, M.J., Pouliquem, Y.B., Lima, M.A. *et al.* (2005) Cytotoxic flavonoids from Platymiscium floribundum. *J Nat Prod*, **68**, 423-426.
316. Fall, D., Duval, R.A., Gleye, C. *et al.* (2004) Chamuvarinin, an acetogenin bearing a tetrahydropyran ring from the roots of Uvaria chamae. *J Nat Prod*, **67**, 1041-1043.
317. Fan, J.T., Kuang, B., Zeng, G.Z. *et al.* (2011) Biologically active arborinane-type triterpenoids and anthraquinones from Rubia yunnanensis. *J Nat Prod*, **74**, 2069-2080.
318. Fan, J.T., Su, J., Peng, Y.M. *et al.* (2010) Rubiyunnanins C-H, cytotoxic cyclic hexapeptides from Rubia yunnanensis inhibiting nitric oxide production and NF-kappaB activation. *Bioorg Med Chem*, **18**, 8226-8234.
319. Fang, L., Du, D., Ding, G.Z. *et al.* (2010) Neolignans and glycosides from the stem bark of Illicium difengpi. *J Nat Prod*, **73**, 818-824.
320. Fang, N. and Casida, J.E. (1999) New bioactive flavonoids and stilbenes in cube resin insecticide. *J Nat Prod*, **62**, 205-210.
321. Fang, P.L., Cao, Y.L., Yan, H. *et al.* (2011) Lindenane disesquiterpenoids with anti-HIV-1 activity from Chloranthus japonicus. *J Nat Prod*, **74**, 1408-1413.

322. Fang, X.P., Anderson, J.E., Smith, D.L. *et al.* (1992) Gigantetronenin and gigantrionenin: novel cytotoxic acetogenins from Goniothalamus giganteus. *J Nat Prod*, **55**, 1655-1663.
323. Farmer, R.L., Biddle, M.M., Nibbs, A.E. *et al.* (2010) Concise syntheses of the abyssinones and discovery of new inhibitors of prostate cancer and MMP-2 expression. *ACS Med Chem Lett*, **1**, 400-405.
324. Fatmawati, S., Ersam, T., Yu, H. *et al.* (2014) 20(S)-Ginsenoside Rh2 as aldose reductase inhibitor from Panax ginseng. *Bioorg Med Chem Lett*, **24**, 4407-4409.
325. Fei, X., Jo, M., Lee, B. *et al.* (2014) Synthesis of xanthone derivatives based on alpha-mangostin and their biological evaluation for anti-cancer agents. *Bioorg Med Chem Lett*, **24**, 2062-2065.
326. Feldman, M., Santos, J. and Grenier, D. (2011) Comparative evaluation of two structurally related flavonoids, isoliquiritigenin and liquiritigenin, for their oral infection therapeutic potential. *J Nat Prod*, **74**, 1862-1867.
327. Felpin, F.X., Girard, S., Vo-Thanh, G. *et al.* (2001) Efficient enantiomeric synthesis of pyrrolidine and piperidine alkaloids from tobacco. *J Org Chem*, **66**, 6305-6312.
328. Feng, T., Li, Y., Cai, X.H. *et al.* (2009) Monoterpenoid indole alkaloids from Alstonia yunnanensis. *J Nat Prod*, **72**, 1836-1841.
329. Feng, Z., Li, X., Zheng, G. *et al.* (2009) Synthesis and activity in enhancing long-term potentiation (LTP) of clausenamide stereoisomers. *Bioorg Med Chem Lett*, **19**, 2112-2115.
330. Fernandes, M.B., Scotti, M.T., Ferreira, M.J. *et al.* (2008) Use of self-organizing maps and molecular descriptors to predict the cytotoxic activity of sesquiterpene lactones. *Eur J Med Chem*, **43**, 2197-2205.
331. Ferrer, J.P., Zampini, I.C., Cuello, A.S. *et al.* (2016) Cytotoxic Compounds from Aerial Organs of Xanthium strumarium. *Nat Prod Commun*, **11**, 371-374.
332. Festa, M., Capasso, A., D'Acunto, C.W. *et al.* (2011) Xanthohumol induces apoptosis in human malignant glioblastoma cells by increasing reactive oxygen species and activating MAPK pathways. *J Nat Prod*, **74**, 2505-2513.
333. Fiebig, M., Duh, C.Y., Pezzuto, J.M. *et al.* (1985) Plant anticancer agents, XLI. Cardiac glycosides from Streblus asper. *J Nat Prod*, **48**, 981-985.
334. Fiehn, O., Kopka, J., Trethewey, R.N. *et al.* (2000) Identification of uncommon plant metabolites based on calculation of elemental compositions using gas chromatography and quadrupole mass spectrometry. *Anal Chem*, **72**, 3573-3580.
335. Fischedick, J.T., Standiford, M., Johnson, D.A. *et al.* (2013) Structure activity relationship of phenolic diterpenes from Salvia officinalis as activators of the nuclear factor E2-related factor 2 pathway. *Bioorg Med Chem*, **21**, 2618-2622.
336. Fliegmann, J., Schroder, G., Schanz, S. *et al.* (1992) Molecular analysis of chalcone and dihydropinosylvin synthase from Scots pine (Pinus sylvestris), and differential regulation of these and related enzyme activities in stressed plants. *Plant Mol Biol*, **18**, 489-503.
337. Flores, N., Jimenez, I.A., Gimenez, A. *et al.* (2008) Benzoic acid derivatives from Piper species and their antiparasitic activity. *J Nat Prod*, **71**, 1538-1543.
338. Fogliani, B., Raharivelomanana, P., Bianchini, J.P. *et al.* (2005) Bioactive ellagitannins from Cunonia macrophylla, an endemic Cunoniaceae from New Caledonia. *Phytochemistry*, **66**, 241-247.
339. Fotie, J., Bohle, D.S., Leimanis, M.L. *et al.* (2006) Lupeol long-chain fatty acid esters with antimalarial activity from Holarrhena floribunda. *J Nat Prod*, **69**, 62-67.
340. Fotie, J., Bohle, D.S., Olivier, M. *et al.* (2007) Trypanocidal and antileishmanial dihydrochelerythrine derivatives from Garcinia lucida. *J Nat Prod*, **70**, 1650-1653.
341. Fraga, B.M., Diaz, C.E., Guadano, A. *et al.* (2005) Diterpenes from Salvia broussonetii transformed roots and their insecticidal activity. *J Agric Food Chem*, **53**, 5200-5206.
342. Frederich, M., Jacquier, M.J., Thepenier, P. *et al.* (2002) Antiplasmodial activity of alkaloids from various strychnos species. *J Nat Prod*, **65**, 1381-1386.
343. Frederich, M., Tits, M., Hayette, M.P. *et al.* (1999) 10'-Hydroxyusambarensine, a new antimalarial bisindole alkaloid from the roots of Strychnos usambarensis. *J Nat Prod*, **62**, 619-621.
344. Fronza, M., Murillo, R., Slusarczyk, S. *et al.* (2011) In vitro cytotoxic activity of abietane diterpenes from Peltodon longipes as well as Salvia miltiorrhiza and Salvia sahendica. *Bioorg Med Chem*, **19**, 4876-4881.
345. Fu, G., Liu, Y., Yu, S. *et al.* (2006) Cytotoxic oxygenated triterpenoid saponins from Symplocos chinensis. *J Nat Prod*, **69**, 1680-1686.
346. Fu, L., Zhang, S., Li, N. *et al.* (2005) Three new triterpenes from Nerium oleander and biological activity of the isolated compounds. *J Nat Prod*, **68**, 198-206.
347. Fu, M., Qiu, S.X., Xu, Y. *et al.* (2013) A new xanthone from the pericarp of Garcinia mangostana. *Nat Prod Commun*, **8**, 1733-1734.

348. Fu, Q., Zan, K., Zhao, M. *et al.* (2010) Triterpene saponins from Clematis chinensis and their potential anti-inflammatory activity. *J Nat Prod*, **73**, 1234-1239.
349. Fu, S., Zhang, F., Zhang, X. *et al.* (2005) Physicochemical characterization of the Strychnos alkaloids by capillary zone electrophoresis. *Anal Sci*, **21**, 1303-1308.
350. Fu, X., Li, X.C., Smillie, T.J. *et al.* (2008) Cycloartane glycosides from Sutherlandia frutescens. *J Nat Prod*, **71**, 1749-1753.
351. Fujioka, T., Furumi, K., Fujii, H. *et al.* (1999) Antiproliferative constituents from umbelliferae plants. V. A new furanocoumarin and falcarindiol furanocoumarin ethers from the root of Angelica japonica. *Chem Pharm Bull (Tokyo)*, **47**, 96-100.
352. Fujita, R., Duan, H. and Takaishi, Y. (2000) Terpenoids from Tripterigyum hypoglaucum. *Phytochemistry*, **53**, 715-722.
353. Fujiwara, A., Mori, T., Iida, A. *et al.* (1998) Antitumor-promoting naphthoquinones from Catalpa ovata. *J Nat Prod*, **61**, 629-632.
354. Funakoshi, M., Sekine, M., Katane, M. *et al.* (2008) Cloning and functional characterization of Arabidopsis thaliana D-amino acid aminotransferase--D-aspartate behavior during germination. *Febs j*, **275**, 1188-1200.
355. Gachet, M.S., Kunert, O., Kaiser, M. *et al.* (2011) Antiparasitic compounds from Cupania cinerea with activities against Plasmodium falciparum and Trypanosoma brucei rhodesiense. *J Nat Prod*, **74**, 559-566.
356. Gafner, S., Bergeron, C., Batcha, L.L. *et al.* (2003) Inhibition of [3H]-LSD binding to 5-HT7 receptors by flavonoids from Scutellaria lateriflora. *J Nat Prod*, **66**, 535-537.
357. Gafner, S., Bergeron, C., Villinski, J.R. *et al.* (2011) Isoflavonoids and coumarins from Glycyrrhiza uralensis: antibacterial activity against oral pathogens and conversion of isoflavans into isoflavan-quinones during purification. *J Nat Prod*, **74**, 2514-2519.
358. Gafner, S., Dietz, B.M., McPhail, K.L. *et al.* (2006) Alkaloids from Eschscholzia californica and their capacity to inhibit binding of [3H]8-Hydroxy-2-(di-N-propylamino)tetralin to 5-HT1A receptors in Vitro. *J Nat Prod*, **69**, 432-435.
359. Gamarnik, A. and Frydman, R.B. (1991) Cadaverine, an Essential Diamine for the Normal Root Development of Germinating Soybean (Glycine max) Seeds. *Plant Physiol*, **97**, 778-785.
360. Gamez, E.J., Luyengi, L., Lee, S.K. *et al.* (1998) Antioxidant flavonoid glycosides from Daphniphyllum calycinum. *J Nat Prod*, **61**, 706-708.
361. Gamiotea-Turro, D., Cuesta-Rubio, O., Prieto-Gonzalez, S. *et al.* (2004) Antioxidative constituents from the leaves of Hypericum styphelioides. *J Nat Prod*, **67**, 869-871.
362. Gan, L.S., Zheng, D.J., Liu, Q. *et al.* (2015) Eight new cycloartane triterpenoids from Beesia calthifolia with hepatoprotective effects against D-galactosamine induced L02 cell damage. *Bioorg Med Chem Lett*, **25**, 3845-3849.
363. Gan, M., Zhang, Y., Lin, S. *et al.* (2008) Glycosides from the root of Iodes cirrhosa. *J Nat Prod*, **71**, 647-654.
364. Gao, F., Li, Y.Y., Wang, D. *et al.* (2012) Diterpenoid alkaloids from the Chinese traditional herbal "Fuzi" and their cytotoxic activity. *Molecules*, **17**, 5187-5194.
365. Gao, G., Lu, Z., Tao, S. *et al.* (2011) Triterpenoid saponins with antifeedant activities from stem bark of Catunaregam spinosa (Rubiaceae) against Plutella xylostella (Plutellidae). *Carbohydr Res*, **346**, 2200-2205.
366. Gao, H., Wu, L., Kuroyanagi, M. *et al.* (2003) Antitumor-promoting constituents from Chaenomeles sinensis KOEHNE and their activities in JB6 mouse epidermal cells. *Chem Pharm Bull (Tokyo)*, **51**, 1318-1321.
367. Gao, J., Caballero-George, C., Wang, B. *et al.* (2009) 5-OHKF and NorKA, depsipeptides from a Hawaiian collection of Bryopsis pennata: binding properties for NorKA to the human neuropeptide Y Y1 receptor. *J Nat Prod*, **72**, 2172-2176.
368. Gao, L., Peng, X.M., Huo, S.X. *et al.* (2015) Memory Enhancement of Acteoside (Verbascoside) in a Senescent Mice Model Induced by a Combination of D-gal and AlCl3. *Phytother Res*, **29**, 1131-1136.
369. Gao, L., Xiang, L., Luo, Y. *et al.* (2010) Gentisides C-K: nine new neuritogenic compounds from the traditional Chinese medicine Gentiana rigescens Franch. *Bioorg Med Chem*, **18**, 6995-7000.
370. Gao, X.M., Pu, J.X., Huang, S.X. *et al.* (2008) Kadcoccilactones A-J, triterpenoids from Kadsura coccinea. *J Nat Prod*, **71**, 1182-1188.
371. Gao, X.M., Wang, R.R., Niu, D.Y. *et al.* (2013) Bioactive dibenzocyclooctadiene lignans from the stems of Schisandra neglecta. *J Nat Prod*, **76**, 1052-1057.
372. Gao, X.M., Yu, T., Cui, M.Z. *et al.* (2012) Identification and evaluation of apoptotic compounds from Garcinia oligantha. *Bioorg Med Chem Lett*, **22**, 2350-2353.

373. Gao, X.M., Yu, T., Lai, F.S. *et al.* (2010) Identification and evaluation of apoptotic compounds from Garcinia paucinervis. *Bioorg Med Chem*, **18**, 4957-4964.
374. Gaspar-Marques, C., Simoes, M.F., Duarte, A. *et al.* (2003) Labdane and kaurane diterpenoids from Plectranthus fruticosus. *J Nat Prod*, **66**, 491-496.
375. Gaspar-Marques, C., Simoes, M.F. and Rodriguez, B. (2004) Further labdane and kaurane diterpenoids and other constituents from Plectranthus fruticosus. *J Nat Prod*, **67**, 614-621.
376. Gatica-Arias, A., Farag, M.A., Stanke, M. *et al.* (2012) Flavonoid production in transgenic hop (Humulus lupulus L.) altered by PAP1/MYB75 from Arabidopsis thaliana L. *Plant Cell Rep*, **31**, 111-119.
377. Ge, X., Ye, G., Li, P. *et al.* (2008) Cytotoxic diterpenoids and sesquiterpenoids from Pteris multifida. *J Nat Prod*, **71**, 227-231.
378. Gentry, E.J., Jampani, H.B., Keshavarz-Shokri, A. *et al.* (1998) Antitubercular natural products: berberine from the roots of commercial Hydrastis canadensis powder. Isolation of inactive 8-oxotetrahydrothalifendine, canadine, beta-hydrastine, and two new quinic acid esters, hycandinic acid esters-1 and -2. *J Nat Prod*, **61**, 1187-1193.
379. Gfeller, A., Laloux, M., Barsics, F. *et al.* (2013) Characterization of volatile organic compounds emitted by barley (Hordeum vulgare L.) roots and their attractiveness to wireworms. *J Chem Ecol*, **39**, 1129-1139.
380. Ghribia, L., Ghouilaa, H., Omrib, A. *et al.* (2014) Antioxidant and anti-acetylcholinesterase activities of extracts and secondary metabolites from Acacia cyanophylla. *Asian Pac J Trop Biomed*, **4**, S417-423.
381. Giner, E., El Alami, M., Manez, S. *et al.* (2011) Phenolic substances from Phagnalon rupestre protect against 2,4,6-trinitrochlorobenzene-induced contact hypersensitivity. *J Nat Prod*, **74**, 1079-1084.
382. Gleye, C., Lewin, G., Laurens, A. *et al.* (2003) Acaricidal activity of tonka bean extracts. Synthesis and structure-activity relationships of bioactive derivatives. *J Nat Prod*, **66**, 690-692.
383. Gonda, R., Takeda, T. and Akiyama, T. (2000) Studies on the constituents of Anaxagorea luzonensis A. GRAY. *Chem Pharm Bull (Tokyo)*, **48**, 1219-1222.
384. Gonzalez-Pena, D., Colina-Coca, C., Char, C.D. *et al.* (2013) Hyaluronidase inhibiting activity and radical scavenging potential of flavonols in processed onion. *J Agric Food Chem*, **61**, 4862-4872.
385. Gonzalez, A.G., Hernandez, J.C., Leon, F. *et al.* (2003) Steroidal saponins from the bark of Dracaena draco and their cytotoxic activities. *J Nat Prod*, **66**, 793-798.
386. Gopalakrishnan, G., Banumathi, B. and Suresh, G. (1997) Evaluation of the antifungal activity of natural xanthones from Garcinia mangostana and their synthetic derivatives. *J Nat Prod*, **60**, 519-524.
387. Govindachari, T.R., Krishna Kumari, G.N., Gopalakrishnan, G. *et al.* (2001) Insect antifeedant and growth regulating activities of quassinoids from Samadera indica. *Fitoterapia*, **72**, 568-571.
388. Gray, C.A., Kaye, P.T. and Nchinda, A.T. (2003) Chromone studies. Part 13. Synthesis and electron-impact mass spectrometric studies of 5-hydroxy-2-isopropyl-7-methoxychromone, a constituent of the medicinal plant Baeckea frutescens, and side-chain analogues. *J Nat Prod*, **66**, 1144-1146.
389. Greenham, J., Vassiliades, D.D., Harborne, J.B. *et al.* (2001) A distinctive flavonoid chemistry for the anomalous genus Biebersteinia. *Phytochemistry*, **56**, 87-91.
390. Grobosch, T., Schwarze, B., Felgenhauer, N. *et al.* (2013) Eight cases of fatal and non-fatal poisoning with Taxus baccata. *Forensic Sci Int*, **227**, 118-126.
391. Grougnet, R., Magiatis, P., Fokialakis, N. *et al.* (2005) Koniamborine, the first pyrano[3,2-b]indole alkaloid and other secondary metabolites from Boronella koniambiensis. *J Nat Prod*, **68**, 1083-1086.
392. Groweiss, A., Cardellina, J.H. and Boyd, M.R. (2000) HIV-Inhibitory prenylated xanthones and flavones from Maclura tinctoria. *J Nat Prod*, **63**, 1537-1539.
393. Gu, C.Z., Lv, J.J., Zhang, X.X. *et al.* (2015) Triterpenoids with Promoting Effects on the Differentiation of PC12 Cells from the Steamed Roots of Panax notoginseng. *J Nat Prod*, **78**, 1829-1840.
394. Gu, J., Sun, X., Wang, G. *et al.* (2011) Icariside II enhances Nrf2 nuclear translocation to upregulate phase II detoxifying enzyme expression coupled with the ERK, Akt and JNK signaling pathways. *Molecules*, **16**, 9234-9244.
395. Gu, J.Q., Graf, T.N., Lee, D. *et al.* (2004) Cytotoxic and antimicrobial constituents of the bark of Diospyros maritima collected in two geographical locations in Indonesia. *J Nat Prod*, **67**, 1156-1161.
396. Gu, J.Q., Wang, Y., Franzblau, S.G. *et al.* (2004) Constituents of Senecio chionophilus with potential antitubercular activity. *J Nat Prod*, **67**, 1483-1487.
397. Guan, S.H., Sattler, I., Lin, W.H. *et al.* (2005) p-Aminoacetophenonic acids produced by a mangrove endophyte: Streptomyces griseus subsp. *J Nat Prod*, **68**, 1198-1200.
398. Guan, S.P., Kong, L.R., Cheng, C. *et al.* (2011) Protective role of 14-deoxy-11,12-didehydroandrographolide, a noncytotoxic analogue of andrographolide, in allergic airway inflammation. *J Nat Prod*, **74**, 1484-1490.

399. Guerram, M., Jiang, Z.Z., Sun, L. *et al.* (2015) Antineoplastic effects of deoxypodophyllotoxin, a potent cytotoxic agent of plant origin, on glioblastoma U-87 MG and SF126 cells. *Pharmacol Rep*, **67**, 245-252.
400. Guerrero, I.C., Andres, L.S., Leon, L.G. *et al.* (2006) Abietane diterpenoids from Salvia pachyphylla and S. clevelandii with cytotoxic activity against human cancer cell lines. *J Nat Prod*, **69**, 1803-1805.
401. Gui, M.Y., Aoyagi, Y., Jin, Y.R. *et al.* (2004) Excisanin H, a novel cytotoxic 14,20-epoxy-ent-kaurene diterpenoid, and three new ent-kaurene diterpenoids from Rabdosia excisa. *J Nat Prod*, **67**, 373-376.
402. Guimaraes, K.G., de Souza Filho, J.D., Dos Mares-Guia, T.R. *et al.* (2008) Dihydroisocoumarin from Xyris pterygoblephara active against dermatophyte fungi. *Phytochemistry*, **69**, 439-444.
403. Guinaudeau, H., Bohlke, M., Lin, L.Z. *et al.* (1997) (+)-Angchibangkine, a new type of bisbenzylisoquinoline alkaloid, and other dimers from Pachygone dasycarpa. *J Nat Prod*, **60**, 258-260.
404. Guinaudeau, H., Lin, L.Z., Ruangrungsi, N. *et al.* (1993) Bisbenzylisoquinoline alkaloids from Cyclea barbata. *J Nat Prod*, **56**, 1989-1992.
405. Gulcin, I., Elias, R., Gepdiremen, A. *et al.* (2010) Antioxidant activity of bisbenzylisoquinoline alkaloids from Stephania rotunda: cepharanthine and fangchinoline. *J Enzyme Inhib Med Chem*, **25**, 44-53.
406. Guo, H., Koike, K., Li, W. *et al.* (2004) Saponins from the flower buds of Buddleja officinalis. *J Nat Prod*, **67**, 10-13.
407. Guo, H., Liu, A.H., Li, L. *et al.* (2007) Simultaneous determination of 12 major constituents in Forsythia suspensa by high performance liquid chromatography--DAD method. *J Pharm Biomed Anal*, **43**, 1000-1006.
408. Guo, H., Zhao, H., Kanno, Y. *et al.* (2013) A dihydrochalcone and several homoisoflavonoids from Polygonatum odoratum are activators of adenosine monophosphate-activated protein kinase. *Bioorg Med Chem Lett*, **23**, 3137-3139.
409. Guo, P., Yan, W., Han, Q. *et al.* (2015) Simultaneous quantification of 25 active constituents in the total flavonoids extract from Herba Desmodii Styracifolii by high-performance liquid chromatography with electrospray ionization tandem mass spectrometry. *J Sep Sci*, **38**, 1156-1163.
410. Ha do, T., Ngoc, T.M., Lee, I. *et al.* (2009) Inhibitors of aldose reductase and formation of advanced glycation end-products in moutan cortex (Paeonia suffruticosa). *J Nat Prod*, **72**, 1465-1470.
411. Hajdu, Z., Hohmann, J., Forgo, P. *et al.* (2007) Diterpenoids and flavonoids from the fruits of Vitex agnus-castus and antioxidant activity of the fruit extracts and their constituents. *Phytother Res*, **21**, 391-394.
412. Hajto, T., Hostanska, K., Fischer, J. *et al.* (1997) Immunomodulatory effects of Viscum album agglutinin-I on natural immunity. *Anticancer Drugs*, **8 Suppl 1**, S43-46.
413. Hakkinen, S.T., Tilleman, S., Swiatek, A. *et al.* (2007) Functional characterisation of genes involved in pyridine alkaloid biosynthesis in tobacco. *Phytochemistry*, **68**, 2773-2785.
414. Halabalaki, M., Alexi, X., Aligiannis, N. *et al.* (2008) Ebenfurans IV-VIII from Onobrychis ebenoides: evidence that C-prenylation is the key determinant of the cytotoxicity of 3-formyl-2-arylbenzofurans. *J Nat Prod*, **71**, 1934-1937.
415. Halabalaki, M., Aligiannis, N., Papoutsi, Z. *et al.* (2000) Three new arylobenzofurans from Onobrychis ebenoides and evaluation of their binding affinity for the estrogen receptor. *J Nat Prod*, **63**, 1672-1674.
416. Hallock, Y.F., Cardellina, J.H., 2nd, Schaffer, M. *et al.* (1998) Korundamine A, a novel HIV-inhibitory and antimalarial "hybrid" naphthylisoquinoline alkaloid heterodimer from Ancistrocladus korupensis. *Bioorg Med Chem Lett*, **8**, 1729-1734.
417. Hallock, Y.F., Manfredi, K.P., Dai, J.R. *et al.* (1997) Michellamines D-F, new HIV-inhibitory dimeric naphthylisoquinoline alkaloids, and korupensamine E, a new antimalarial monomer, from Ancistrocladus korupensis. *J Nat Prod*, **60**, 677-683.
418. Hamao, M., Matsuda, H., Nakamura, S. *et al.* (2011) Anti-obesity effects of the methanolic extract and chakasaponins from the flower buds of Camellia sinensis in mice. *Bioorg Med Chem*, **19**, 6033-6041.
419. Hamburger, M.O., Cordell, G.A., Tantivatana, P. *et al.* (1987) Traditional medicinal plants of Thailand, VIII. Isoflavonoids of Dalbergia candenatensis. *J Nat Prod*, **50**, 696-699.
420. Han, A.R., Kang, Y.J., Windono, T. *et al.* (2006) Prenylated flavonoids from the heartwood of Artocarpus communis with inhibitory activity on lipopolysaccharide-induced nitric oxide production. *J Nat Prod*, **69**, 719-721.
421. Han, A.R., Kim, J.A., Lantvit, D.D. *et al.* (2009) Cytotoxic xanthone constituents of the stem bark of Garcinia mangostana (mangosteen). *J Nat Prod*, **72**, 2028-2031.
422. Han, N., Huang, T., Wang, Y.C. *et al.* (2011) Flavanone glycosides from viscum coloratum and their inhibitory effects on osteoclast formation. *Chem Biodivers*, **8**, 1682-1688.
423. Han, X.H., Hong, S.S., Jin, Q. *et al.* (2009) Prenylated and benzylated flavonoids from the fruits of Cudrania tricuspidata. *J Nat Prod*, **72**, 164-167.

424. Hanakova, Z., Hosek, J., Babula, P. *et al.* (2015) C-Geranylated Flavanones from Paulownia tomentosa Fruits as Potential Anti-inflammatory Compounds Acting via Inhibition of TNF-alpha Production. *J Nat Prod*, **78**, 850-863.
425. Hao, Z.Y., Liang, D., Luo, H. *et al.* (2012) Bioactive sesquiterpenoids from the rhizomes of Acorus calamus. *J Nat Prod*, **75**, 1083-1089.
426. Hari Babu, T., Manjulatha, K., Suresh Kumar, G. *et al.* (2010) Gastroprotective flavonoid constituents from Oroxylum indicum Vent. *Bioorg Med Chem Lett*, **20**, 117-120.
427. Harinantenaina, L., Asakawa, Y. and De Clercq, E. (2007) Cinnamacrins A-C, cinnafragrin D, and cytostatic metabolites with alpha-glucosidase inhibitory activity from Cinnamosma macrocarpa. *J Nat Prod*, **70**, 277-282.
428. Harinantenaina, L., Brodie, P.J., Slebodnick, C. *et al.* (2010) Antiproliferative compounds from Pongamiopsis pervilleana from the Madagascar Dry Forest. *J Nat Prod*, **73**, 1559-1562.
429. Harinantenaina, L., Matsunami, K., Otsuka, H. *et al.* (2008) Secondary metabolites of Cinnamosma madagascariensis and their alpha-glucosidase inhibitory properties. *J Nat Prod*, **71**, 123-126.
430. Harrigan, G.G., Gunatilaka, A.A., Kingston, D.G. *et al.* (1994) Isolation of bioactive and other oxoaporphine alkaloids from two annonaceous plants, Xylopia aethiopica and Miliusa cf. banacea. *J Nat Prod*, **57**, 68-73.
431. Hashimoto, T., Suganuma, M., Fujiki, H. *et al.* (2003) Isolation and synthesis of TNF-alpha release inhibitors from Fijian kawa (Piper methysticum). *Phytomedicine*, **10**, 309-317.
432. Hasrat, J.A., Pieters, L., Claeys, M. *et al.* (1997) Adenosine-1 active ligands: cirsimarin, a flavone glycoside from Microtea debilis. *J Nat Prod*, **60**, 638-641.
433. Hay, A.E., Ioset, J.R., Ahua, K.M. *et al.* (2007) Limonoid orthoacetates and antiprotozoal compounds from the roots of Pseudocedrela kotschyi. *J Nat Prod*, **70**, 9-13.
434. Hayashi, K., Hayashi, T., Otsuka, H. *et al.* (1997) Antiviral activity of 5,6,7-trimethoxyflavone and its potentiation of the antiherpes activity of acyclovir. *J Antimicrob Chemother*, **39**, 821-824.
435. Hayashi, T., Asano, S., Mizutani, M. *et al.* (1991) Scopadulciol, an inhibitor of gastric H+, K(+)-ATPase from Scoparia dulcis, and its structure-activity relationships. *J Nat Prod*, **54**, 802-809.
436. Hayashi, T., Kawasaki, M., Okamura, K. *et al.* (1992) Scoparic acid A, a beta-glucuronidase inhibitor from Scoparia dulcis. *J Nat Prod*, **55**, 1748-1755.
437. Hazawa, M., Takahashi, K., Sugata, S. *et al.* (2011) (-)-Epigallocatechin-3-O-gallate induces nonapoptotic cell death in leukemia cells independent of the 67 kDa laminin receptor. *J Nat Prod*, **74**, 695-700.
438. He, C.M., Cheng, Z.H. and Chen, D.F. (2013) Qualitative and quantitative analysis of flavonoids in Sophora tonkinensis by LC/MS and HPLC. *Chin J Nat Med*, **11**, 690-698.
439. He, F., Wang, M., Gao, M. *et al.* (2014) Chemical composition and biological activities of Gerbera anandria. *Molecules*, **19**, 4046-4057.
440. He, J., Chen, L., Heber, D. *et al.* (2006) Antibacterial compounds from Glycyrrhiza uralensis. *J Nat Prod*, **69**, 121-124.
441. He, K., Zeng, L., Shi, G. *et al.* (1997) Bioactive compounds from Taiwania cryptomerioides. *J Nat Prod*, **60**, 38-40.
442. He, X.F., Yin, S., Ji, Y.C. *et al.* (2010) Sesquiterpenes and dimeric sesquiterpenoids from Sarcandra glabra. *J Nat Prod*, **73**, 45-50.
443. Hegde, V.R., Borges, S., Patel, M. *et al.* (2010) New potential antitumor compounds from the plant Aristolochia manshuriensis as inhibitors of the CDK2 enzyme. *Bioorg Med Chem Lett*, **20**, 1344-1346.
444. Hehmann, M., Lukacin, R., Ekiert, H. *et al.* (2004) Furanocoumarin biosynthesis in Ammi majus L. Cloning of bergaptol O-methyltransferase. *Eur J Biochem*, **271**, 932-940.
445. Henry, G.E., Raithore, S., Zhang, Y. *et al.* (2006) Acylphloroglucinol derivatives from Hypericum prolificum. *J Nat Prod*, **69**, 1645-1648.
446. Hernandez-Romero, Y., Rojas, J.I., Castillo, R. *et al.* (2004) Spasmolytic effects, mode of action, and structure-activity relationships of stilbenoids from Nidema boothii. *J Nat Prod*, **67**, 160-167.
447. Herraiz, T. (1999) 1-methyl-1,2,3,4-tetrahydro-beta-carboline-3-carboxylic acid and 1,2, 3,4-tetrahydro-beta-carboline-3-carboxylic acid in fruits. *J Agric Food Chem*, **47**, 4883-4887.
448. Hirasawa, Y., Kato, E., Kobayashi, J. *et al.* (2008) Lycoparins A-C, new alkaloids from Lycopodium casuarinoides inhibiting acetylcholinesterase. *Bioorg Med Chem*, **16**, 6167-6171.
449. Hisamatsu, Y., Goto, N., Sekiguchi, M. *et al.* (2005) Oxylipins arabidopsides C and D from Arabidopsis thaliana. *J Nat Prod*, **68**, 600-603.
450. Ho, C.L., Yang, S.S., Chang, T.M. *et al.* (2012) Composition, antioxidant, antimicrobial and anti-wood-decay fungal activities of the twig essential oil of Taiwania cryptomerioides from Taiwan. *Nat Prod Commun*, **7**, 261-264.

451. Hodges, T.W., Hossain, C.F., Kim, Y.P. *et al.* (2004) Molecular-targeted antitumor agents: the Saururus cernuus dineolignans manassantin B and 4-O-demethylmanassantin B are potent inhibitors of hypoxia-activated HIF-1. *J Nat Prod*, **67**, 767-771.
452. Hoenicke, K., Borchert, O., Gruning, K. *et al.* (2002) "Untypical aging off-flavor" in wine: synthesis of potential degradation compounds of indole-3-acetic acid and kynurenine and their evaluation as precursors of 2-aminoacetophenone. *J Agric Food Chem*, **50**, 4303-4309.
453. Hong, H., Chen, H.B., Yang, D.H. *et al.* (2011) Comparison of contents of five ephedrine alkaloids in three official origins of Ephedra Herb in China by high-performance liquid chromatography. *J Nat Med*, **65**, 623-628.
454. Hong, J.Y., Chung, H.J., Lee, H.J. *et al.* (2011) Growth inhibition of human lung cancer cells via down-regulation of epidermal growth factor receptor signaling by yuanhuadine, a daphnane diterpene from Daphne genkwa. *J Nat Prod*, **74**, 2102-2108.
455. Hong, S.S., Lee, S.A., Han, X.H. *et al.* (2008) ent-Kaurane diterpenoids from Isodon japonicus. *J Nat Prod*, **71**, 1055-1058.
456. Hopp, D.C., Zeng, L., Gu, Z.M. *et al.* (1997) Novel mono-tetrahydrofuran ring acetogenins, from the bark of Annona squamosa, showing cytotoxic selectivities for the human pancreatic carcinoma cell line, PACA-2. *J Nat Prod*, **60**, 581-586.
457. Horiuch, M., Murakami, C., Fukamiya, N. *et al.* (2006) Tripfordines A-C, sesquiterpene pyridine alkaloids from Tripterygium wilfordii, and structure anti-HIV activity relationships of Tripterygium alkaloids. *J Nat Prod*, **69**, 1271-1274.
458. Hosek, J., Bartos, M., Chudik, S. *et al.* (2011) Natural compound cudraflavone B shows promising anti-inflammatory properties in vitro. *J Nat Prod*, **74**, 614-619.
459. Hou, J., Dong, H., Yan, M. *et al.* (2014) New guaiane sesquiterpenes from Artemisia rupestris and their inhibitory effects on nitric oxide production. *Bioorg Med Chem Lett*, **24**, 4435-4438.
460. Hou, J.Q., Guo, C., Zhao, J.J. *et al.* (2017) Anti-inflammatory Meroterpenoids from Baeckea frutescens. *J Nat Prod*, **80**, 2204-2214.
461. Hou, Y., Cao, S., Brodie, P. *et al.* (2009) Antiproliferative cardenolide glycosides of Elaeodendron alluaudianum from the Madagascar Rainforest. *Bioorg Med Chem*, **17**, 2215-2218.
462. Hsieh, P.W., Huang, Z.Y., Chen, J.H. *et al.* (2007) Cytotoxic withanolides from Tubocapsicum anomalum. *J Nat Prod*, **70**, 747-753.
463. Hsieh, T.J., Chang, F.R., Chia, Y.C. *et al.* (2001) The alkaloids of Artabotrys uncinatus. *J Nat Prod*, **64**, 1157-1161.
464. Hsieh, Y.J., Lin, L.C. and Tsai, T.H. (2005) Determination and identification of plumbagin from the roots of Plumbago zeylanica L. by liquid chromatography with tandem mass spectrometry. *J Chromatogr A*, **1083**, 141-145.
465. Hu, C., Tohge, T., Chan, S.A. *et al.* (2016) Identification of Conserved and Diverse Metabolic Shifts during Rice Grain Development. *Sci Rep*, **6**, 20942.
466. Hu, C.Q., Chen, K., Shi, Q. *et al.* (1994) Anti-AIDS agents, 10. Acacetin-7-O-beta-D-galactopyranoside, an anti-HIV principle from Chrysanthemum morifolium and a structure-activity correlation with some related flavonoids. *J Nat Prod*, **57**, 42-51.
467. Hu, J.F., Garo, E., Goering, M.G. *et al.* (2006) Bacterial biofilm inhibitors from Diospyros dendo. *J Nat Prod*, **69**, 118-120.
468. Hu, Q.F., Zhou, B., Huang, J.M. *et al.* (2013) Antiviral phenolic compounds from Arundina gramnifolia. *J Nat Prod*, **76**, 292-296.
469. Hu, Q.F., Zhou, B., Huang, J.M. *et al.* (2013) Cytotoxic oxepinochromenone and flavonoids from the flower buds of Rosa rugosa. *J Nat Prod*, **76**, 1866-1871.
470. Hu, X., Wu, J.W., Wang, M. *et al.* (2012) 2-Arylbenzofuran, flavonoid, and tyrosinase inhibitory constituents of Morus yunnanensis. *J Nat Prod*, **75**, 82-87.
471. Hu, Y.M., Liu, C., Cheng, K.W. *et al.* (2008) Sesquiterpenoids from Homalomena occulta affect osteoblast proliferation, differentiation and mineralization in vitro. *Phytochemistry*, **69**, 2367-2373.
472. Huang, H., Morgan, C.M., Asolkar, R.N. *et al.* (2010) Phytotoxicity of sarmentine isolated from long pepper (Piper longum) fruit. *J Agric Food Chem*, **58**, 9994-10000.
473. Huang, H., She, Z., Lin, Y. *et al.* (2007) Cyclic peptides from an endophytic fungus obtained from a mangrove leaf (Kandelia candel). *J Nat Prod*, **70**, 1696-1699.
474. Huang, H.L., Wang, C.M., Wang, Z.H. *et al.* (2011) Tirucallane-type triterpenoids from Dysoxylum lenticellatum. *J Nat Prod*, **74**, 2235-2242.
475. Huang, N.K., Chern, Y., Fang, J.M. *et al.* (2007) Neuroprotective principles from Gastrodia elata. *J Nat Prod*, **70**, 571-574.
476. Huang, S.C., Yen, G.C., Chang, L.W. *et al.* (2003) Identification of an antioxidant, ethyl protocatechuate, in peanut seed testa. *J Agric Food Chem*, **51**, 2380-2383.

477. Huang, S.X., Feng, C., Zhou, Y. *et al.* (2009) Bioassay-guided isolation of xanthones and polycyclic prenylated acylphloroglucinols from Garcinia oblongifolia. *J Nat Prod*, **72**, 130-135.
478. Huang, X.X., Zhou, C.C., Li, L.Z. *et al.* (2013) The cytotoxicity of 8-O-4' neolignans from the seeds of Crataegus pinnatifida. *Bioorg Med Chem Lett*, **23**, 5599-5604.
479. Huang, Y.T., Chang, H.S., Wang, G.J. *et al.* (2011) Anti-inflammatory endiandric acid analogues from the roots of Beilschmiedia tsangii. *J Nat Prod*, **74**, 1875-1880.
480. Hung, T.M., Thu, C.V., Dat, N.T. *et al.* (2010) Homoisoflavonoid derivatives from the roots of Ophiopogon japonicus and their in vitro anti-inflammation activity. *Bioorg Med Chem Lett*, **20**, 2412-2416.
481. Huo, J., Yang, S.P., Ding, J. *et al.* (2004) Cytotoxic sesquiterpene lactones from Eupatorium lindleyanum. *J Nat Prod*, **67**, 1470-1475.
482. Hussein, G., Miyashiro, H., Nakamura, N. *et al.* (2000) Inhibitory effects of sudanese medicinal plant extracts on hepatitis C virus (HCV) protease. *Phytother Res*, **14**, 510-516.
483. Hwang, B.Y., Kim, H.S., Lee, J.H. *et al.* (2001) Antioxidant benzoylated flavan-3-ol glycoside from Celastrus orbiculatus. *J Nat Prod*, **64**, 82-84.
484. Hwang, J.H., Hong, S.S., Han, X.H. *et al.* (2007) Prenylated xanthones from the root bark of Cudrania tricuspidata. *J Nat Prod*, **70**, 1207-1209.
485. Hymavathi, A., Devanand, P., Suresh Babu, K. *et al.* (2011) Vapor-phase toxicity of Derris scandens Benth.-derived constituents against four stored-product pests. *J Agric Food Chem*, **59**, 1653-1657.
486. Ibrahim, M.A., Mansoor, A.A., Gross, A. *et al.* (2009) Methicillin-resistant Staphylococcus aureus (MRSA)-active metabolites from Platanus occidentalis (American Sycamore). *J Nat Prod*, **72**, 2141-2144.
487. Ichimaru, M., Nakatani, N., Takahashi, T. *et al.* (2004) Cytotoxic C-benzylated dihydrochalcones from Uvaria acuminata. *Chem Pharm Bull (Tokyo)*, **52**, 138-141.
488. Ikeda, T., Fujiwara, S., Araki, K. *et al.* (1997) Cytotoxic glycosides from Albizia julibrissin. *J Nat Prod*, **60**, 102-107.
489. Ikezawa, N., Iwasa, K. and Sato, F. (2007) Molecular cloning and characterization of methylenedioxy bridge-forming enzymes involved in stylopine biosynthesis in Eschscholzia californica. *Febs j*, **274**, 1019-1035.
490. Inbaraj, J.J. and Chignell, C.F. (2004) Cytotoxic action of juglone and plumbagin: a mechanistic study using HaCaT keratinocytes. *Chem Res Toxicol*, **17**, 55-62.
491. Inoue, M., Tanabe, H., Nakashima, K. *et al.* (2014) Rexinoids isolated from Sophora tonkinensis with a gene expression profile distinct from the synthetic rexinoid bexarotene. *J Nat Prod*, **77**, 1670-1677.
492. Inoue, T., Shinbori, T., Fujioka, M. *et al.* (1978) [Studies on the pungent principle of Alpinia officinarum Hance (author's transl)]. *Yakugaku Zasshi*, **98**, 1255-1257.
493. Isakovic, A., Jankovic, T., Harhaji, L. *et al.* (2008) Antiglioma action of xanthones from Gentiana kochiana: Mechanistic and structure-activity requirements. *Bioorg Med Chem*, **16**, 5683-5694.
494. Ishida, J., Wang, H.K., Oyama, M. *et al.* (2001) Anti-AIDS agents. 46. Anti-HIV activity of harman, an anti-HIV principle from Symplocos setchuensis, and its derivatives. *J Nat Prod*, **64**, 958-960.
495. Ito, A., Chai, H.B., Kardono, L.B. *et al.* (2004) Saponins from the bark of Nephelium maingayi. *J Nat Prod*, **67**, 201-205.
496. Ito, C., Itoigawa, M., Aizawa, K. *et al.* (2009) Gamma-lactone carbazoles from Clausena anisata. *J Nat Prod*, **72**, 1202-1204.
497. Ito, C., Itoigawa, M., Kumagaya, M. *et al.* (2006) Isoflavonoids with antiestrogenic activity from Millettia pachycarpa. *J Nat Prod*, **69**, 138-141.
498. Ito, C., Itoigawa, M., Otsuka, T. *et al.* (2000) Constituents of Boronia pinnata. *J Nat Prod*, **63**, 1344-1348.
499. Ito, C., Katsuno, S., Itoigawa, M. *et al.* (2000) New carbazole alkaloids from Clausena anisata with antitumor promoting activity. *J Nat Prod*, **63**, 125-128.
500. Itoigawa, M., Kashiwada, Y., Ito, C. *et al.* (2000) Antitumor agents. 203. Carbazole alkaloid murrayaquinone A and related synthetic carbazolequinones as cytotoxic agents. *J Nat Prod*, **63**, 893-897.
501. Ivanov, A., Kameka, A., Pajak, A. *et al.* (2012) Arabidopsis mutants lacking asparaginases develop normally but exhibit enhanced root inhibition by exogenous asparagine. *Amino Acids*, **42**, 2307-2318.
502. Iwagawa, T., Nakashima, R., Takayama, K. *et al.* (1999) New cembranes from the soft coral sarcophyton species. *J Nat Prod*, **62**, 1046-1049.
503. Iwai, Y., Murakami, K., Gomi, Y. *et al.* (2011) Anti-influenza activity of marchantins, macrocyclic bisbibenzyls contained in liverworts. *PLoS One*, **6**, e19825.

504. Iwanaga, A., Kusano, G., Warashina, T. *et al.* (2010) Hyaluronidase inhibitors from "Cimicifugae Rhizoma" (a mixture of the rhizomes of Cimicifuga dahurica and C. heracleifolia). *J Nat Prod*, **73**, 573-578.
505. Iwata, N., Wang, N., Yao, X. *et al.* (2004) Structures and histamine release inhibitory effects of prenylated orcinol derivatives from Rhododendron dauricum. *J Nat Prod*, **67**, 1106-1109.
506. Jahaniani, F., Ebrahimi, S.A., Rahbar-Roshandel, N. *et al.* (2005) Xanthomicrol is the main cytotoxic component of Dracocephalum kotschyii and a potential anti-cancer agent. *Phytochemistry*, **66**, 1581-1592.
507. Jaiswal, Y., Liang, Z., Yong, P. *et al.* (2013) A comparative study on the traditional Indian Shodhana and Chinese processing methods for aconite roots by characterization and determination of the major components. *Chem Cent J*, **7**, 169.
508. Jang, D.S., Park, E.J., Hawthorne, M.E. *et al.* (2003) Potential cancer chemopreventive constituents of the seeds of Dipteryx odorata (tonka bean). *J Nat Prod*, **66**, 583-587.
509. Jang, H.Y., Ahn, K.S., Park, M.J. *et al.* (2012) Skullcapflavone II inhibits ovalbumin-induced airway inflammation in a mouse model of asthma. *Int Immunopharmacol*, **12**, 666-674.
510. Jantan, I., Pisar, M.M., Idris, M.S. *et al.* (2002) In vitro inhibitory effect of rubraxanthone isolated from Garcinia parvifolia on platelet-activating factor receptor binding. *Planta Med*, **68**, 1133-1134.
511. Jao, C.W., Lin, W.C., Wu, Y.T. *et al.* (2008) Isolation, structure elucidation, and synthesis of cytotoxic tryptanthrin analogues from Phaius mishmensis. *J Nat Prod*, **71**, 1275-1279.
512. Jayasuriya, H., Herath, K.B., Ondeyka, J.G. *et al.* (2005) Diterpenoid, steroid, and triterpenoid agonists of liver X receptors from diversified terrestrial plants and marine sources. *J Nat Prod*, **68**, 1247-1252.
513. Jayasuriya, H., Zink, D.L., Borris, R.P. *et al.* (2004) Rediocides B-E, potent insecticides from Trigonostemon reidioides. *J Nat Prod*, **67**, 228-231.
514. Jeong, E.J., Cho, J.H., Sung, S.H. *et al.* (2011) Inhibition of nitric oxide production in lipopolysaccharide-stimulated RAW264.7 macrophage cells by lignans isolated from Euonymus alatus leaves and twigs. *Bioorg Med Chem Lett*, **21**, 2283-2286.
515. Jeong, G.S., Lee, D.S., Kwon, T.O. *et al.* (2009) Cytoprotective constituents of the heartwood of Caesalpinia sappan on glutamate-induced oxidative damage in HT22 cells. *Biol Pharm Bull*, **32**, 945-949.
516. Jeong, H.J., Lee, C.S., Choi, J. *et al.* (2015) Flavokawains B and C, melanogenesis inhibitors, isolated from the root of Piper methysticum and synthesis of analogs. *Bioorg Med Chem Lett*, **25**, 799-802.
517. Jeong, H.J., Ryu, Y.B., Park, S.J. *et al.* (2009) Neuraminidase inhibitory activities of flavonols isolated from Rhodiola rosea roots and their in vitro anti-influenza viral activities. *Bioorg Med Chem*, **17**, 6816-6823.
518. Jeong, J.Y., Jo, Y.H., Kim, S.B. *et al.* (2015) Pancreatic lipase inhibitory constituents from Morus alba leaves and optimization for extraction conditions. *Bioorg Med Chem Lett*, **25**, 2269-2274.
519. Jia, R., Guo, Y.W., Mollo, E. *et al.* (2006) Sarcophytonolides E-H, cembranolides from the Hainan soft coral Sarcophyton latum. *J Nat Prod*, **69**, 819-822.
520. Jiang, B., Chi, C., Fu, Y.W. *et al.* (2013) In vivo anthelmintic effect of flavonol rhamnosides from Dryopteris crassirhizoma against Dactylogyrus intermedius in goldfish (Carassius auratus). *Parasitol Res*, **112**, 4097-4104.
521. Jiang, H.Y., Wang, W.G., Zhou, M. *et al.* (2013) Enmein-type 6,7-seco-ent-kauranoids from Isodon sculponeatus. *J Nat Prod*, **76**, 2113-2119.
522. Jiang, R.W., Zhou, J.R., Hon, P.M. *et al.* (2007) Lignans from Dysosma versipellis with inhibitory effects on prostate cancer cell lines. *J Nat Prod*, **70**, 283-286.
523. Jiang, Y.Y., Liu, B., Shi, R.B. *et al.* (2007) [Isolation and structure identification of chemical constituents from Saposhnikovia divaricata (Turcz.) Schischk]. *Yao Xue Xue Bao*, **42**, 505-510.
524. Jiang, Z.H., Wang, J.R., Li, M. *et al.* (2005) Hemiterpene glucosides with anti-platelet aggregation activities from Ilex pubescens. *J Nat Prod*, **68**, 397-399.
525. Jiang, Z.H., Wen, X.Y., Tanaka, T. *et al.* (2008) Cytotoxic hydrolyzable tannins from Balanophora japonica. *J Nat Prod*, **71**, 719-723.
526. Jiang, Z.Y., Liu, W.F., Zhang, X.M. *et al.* (2013) Anti-HBV active constituents from Piper longum. *Bioorg Med Chem Lett*, **23**, 2123-2127.
527. Jiao, R.H., Ge, H.M., Shi da, H. *et al.* (2006) An apigenin-derived xanthine oxidase inhibitor from Palhinhaea cernua. *J Nat Prod*, **69**, 1089-1091.
528. Jiao, W.H., Gao, H., Li, C.Y. *et al.* (2010) Quassidines A-D, bis-beta-carboline alkaloids from the stems of Picrasma quassioides. *J Nat Prod*, **73**, 167-171.
529. Jilani, K., Abed, M., Zelenak, C. *et al.* (2011) Triggering of erythrocyte cell membrane scrambling by ursolic acid. *J Nat Prod*, **74**, 2181-2186.

530. Jin, H.Z., Hwang, B.Y., Kim, H.S. *et al.* (2002) Antiinflammatory constituents of Celastrus orbiculatus inhibit the NF-kappaB activation and NO production. *J Nat Prod*, **65**, 89-91.
531. Jin, H.Z., Wang, X.L., Wang, H.B. *et al.* (2008) Morphinane alkaloid dimers from Sinomenium acutum. *J Nat Prod*, **71**, 127-129.
532. Jin, J.M., Zhang, Y.J., Li, H.Z. *et al.* (2004) Cytotoxic steroidal saponins from Polygonatum zanlanscianense. *J Nat Prod*, **67**, 1992-1995.
533. Jin, Q., Han, X.H., Hong, S.S. *et al.* (2012) Antioxidative oligostilbenes from Caragana sinica. *Bioorg Med Chem Lett*, **22**, 973-976.
534. Jin, Q., Lee, C., Lee, J.W. *et al.* (2014) 2-Phenoxychromones and prenylflavonoids from Epimedium koreanum and their inhibitory effects on LPS-induced nitric oxide and interleukin-1beta production. *J Nat Prod*, **77**, 1724-1728.
535. Jo, Y.H., Kim, S.B., Liu, Q. *et al.* (2015) Benzylated and prenylated flavonoids from the root barks of Cudrania tricuspidata with pancreatic lipase inhibitory activity. *Bioorg Med Chem Lett*, **25**, 3455-3457.
536. Jo, Y.H., Shin, B., Liu, Q. *et al.* (2014) Antiproliferative prenylated xanthones and benzophenones from the roots of Cudrania tricuspidata in HSC-T6 cells. *J Nat Prod*, **77**, 2361-2366.
537. Joa, H., Vogl, S., Atanasov, A.G. *et al.* (2011) Identification of ostruthin from Peucedanum ostruthium rhizomes as an inhibitor of vascular smooth muscle cell proliferation. *J Nat Prod*, **74**, 1513-1516.
538. Johansen, K.T., Wubshet, S.G., Nyberg, N.T. *et al.* (2011) From retrospective assessment to prospective decisions in natural product isolation: HPLC-SPE-NMR analysis of Carthamus oxyacantha. *J Nat Prod*, **74**, 2454-2461.
539. Jong, T.T., Hwang, H.C., Jean, M.Y. *et al.* (1992) An antiplatelet aggregation principle and X-ray structural analysis of cis-khellactone diester from Peucedanum japonicum. *J Nat Prod*, **55**, 1396-1401.
540. Jonville, M.C., Capel, M., Frederich, M. *et al.* (2008) Fagraldehyde, a secoiridoid isolated from Fagraea fragrans. *J Nat Prod*, **71**, 2038-2040.
541. Joshi, R., Poonam, Saini, R. *et al.* (2011) Characterization of volatile components of tea flowers (Camellia sinensis) growing in Kangra by GC/MS. *Nat Prod Commun*, **6**, 1155-1158.
542. Juan, M.E., Wenzel, U., Daniel, H. *et al.* (2008) Erythrodiol, a natural triterpenoid from olives, has antiproliferative and apoptotic activity in HT-29 human adenocarcinoma cells. *Mol Nutr Food Res*, **52**, 595-599.
543. Juck, D.B., De Rezende, L.C., David, J.P. *et al.* (2006) Two new isoflavonoids from Bowdichia virgilioides. *Nat Prod Res*, **20**, 27-30.
544. Juergenliemk, G., Boje, K., Huewel, S. *et al.* (2003) In vitro studies indicate that miquelianin (quercetin 3-O-beta-D-glucuronopyranoside) is able to reach the CNS from the small intestine. *Planta Med*, **69**, 1013-1017.
545. Jung, H.J., Kang, S.S., Woo, J.J. *et al.* (2005) A new lavandulylated flavonoid with free radical and ONOO- scavenging activities from Sophora flavescens. *Arch Pharm Res*, **28**, 1333-1336.
546. Jung, J.H., Lee, C.O., Kim, Y.C. *et al.* (1996) New bioactive cerebrosides from Arisaema amurense. *J Nat Prod*, **59**, 319-322.
547. Jung, J.W., Ko, W.M., Park, J.H. *et al.* (2015) Isoprenylated flavonoids from the root bark of Morus alba and their hepatoprotective and neuroprotective activities. *Arch Pharm Res*, **38**, 2066-2075.
548. Jung, M.J., Kang, S.S., Jung, H.A. *et al.* (2004) Isolation of flavonoids and a cerebroside from the stem bark of Albizzia julibrissin. *Arch Pharm Res*, **27**, 593-599.
549. Junio, H.A., Sy-Cordero, A.A., Ettefagh, K.A. *et al.* (2011) Synergy-directed fractionation of botanical medicines: a case study with goldenseal (Hydrastis canadensis). *J Nat Prod*, **74**, 1621-1629.
550. Kagan, I.A., Rimando, A.M. and Dayan, F.E. (2003) Chromatographic separation and in vitro activity of sorgoleone congeners from the roots of sorghum bicolor. *J Agric Food Chem*, **51**, 7589-7595.
551. Kalani, K., Kushwaha, V., Verma, R. *et al.* (2013) Glycyrrhetinic acid and its analogs: a new class of antifilarial agents. *Bioorg Med Chem Lett*, **23**, 2566-2570.
552. Kamel, M.S., Mohamed, K.M., Hassanean, H.A. *et al.* (2001) Acylated flavonoid glycosides from Bassia muricata. *Phytochemistry*, **57**, 1259-1262.
553. Kaneda, N., Pezzuto, J.M., Kinghorn, A.D. *et al.* (1992) Plant anticancer agents, L. cytotoxic triterpenes from Sandoricum koetjape stems. *J Nat Prod*, **55**, 654-659.
554. Kang, C., Han, J.H., Oh, J. *et al.* (2015) Steroidal Alkaloids from Veratrum nigrum Enhance Glucose Uptake in Skeletal Muscle Cells. *J Nat Prod*, **78**, 803-810.
555. Kang, H., Jang, S.W., Pak, J.H. *et al.* (2015) Glaucine inhibits breast cancer cell migration and invasion by inhibiting MMP-9 gene expression through the suppression of NF-kappaB activation. *Mol Cell Biochem*, **403**, 85-94.
556. Kang, K., Lee, H.J., Kim, C.Y. *et al.* (2007) The chemopreventive effects of Saussurea salicifolia through induction of apoptosis and phase II detoxification enzyme. *Biol Pharm Bull*, **30**, 2352-2359.

557. Kang, S.H., Jung, H., Kim, N. *et al.* (2000) Micellar electrokinetic chromatography for the analysis of D-amygdalin and its epimer in apricot kernel. *J Chromatogr A*, **866**, 253-259.
558. Kang, S.Y., Lee, K.Y., Sung, S.H. *et al.* (2005) Four new neuroprotective dihydropyranocoumarins from Angelica gigas. *J Nat Prod*, **68**, 56-59.
559. Kang, S.Y., Lee, K.Y., Sung, S.H. *et al.* (2001) Coumarins isolated from Angelica gigas inhibit acetylcholinesterase: structure-activity relationships. *J Nat Prod*, **64**, 683-685.
560. Kang, T.H., Han, N.R., Kim, H.M. *et al.* (2011) Blockade of IL-6 secretion pathway by the sesquiterpenoid atractylenolide III. *J Nat Prod*, **74**, 223-227.
561. Kang, T.H., Jeong, S.J., Ko, W.G. *et al.* (2000) Cytotoxic lavandulyl flavanones from Sophora flavescens. *J Nat Prod*, **63**, 680-681.
562. Kang, Y.J., Chung, H.J., Nam, J.W. *et al.* (2011) Cytotoxic and antineoplastic activity of timosaponin A-III for human colon cancer cells. *J Nat Prod*, **74**, 701-706.
563. Kanokmedhakul, S., Kanokmedhakul, K., Kantikeaw, I. *et al.* (2006) 2-substituted furans from the roots of Polyalthia evecta. *J Nat Prod*, **69**, 68-72.
564. Kape, R., Parniske, M., Brandt, S. *et al.* (1992) Isoliquiritigenin, a strong nod gene- and glyceollin resistance-inducing flavonoid from soybean root exudate. *Appl Environ Microbiol*, **58**, 1705-1710.
565. Kardono, L.B., Angerhofer, C.K., Tsauri, S. *et al.* (1991) Cytotoxic and antimalarial constituents of the roots of Eurycoma longifolia. *J Nat Prod*, **54**, 1360-1367.
566. Karioti, A., Protopappa, A., Megoulas, N. *et al.* (2007) Identification of tyrosinase inhibitors from Marrubium velutinum and Marrubium cylleneum. *Bioorg Med Chem*, **15**, 2708-2714.
567. Karthivashan, G., Tangestani Fard, M., Arulselvan, P. *et al.* (2013) Identification of bioactive candidate compounds responsible for oxidative challenge from hydro-ethanolic extract of Moringa oleifera leaves. *J Food Sci*, **78**, C1368-1375.
568. Kasahara, Y., Itou, T., Numazawa, T. *et al.* (2013) [Aconitine analogues in wild Aconitum plants: contents toxicity to mice and decrease by boiling]. *Shokuhin Eiseigaku Zasshi*, **54**, 364-369.
569. Kashiwada, Y., Nishizawa, M., Yamagishi, T. *et al.* (1995) Anti-AIDS agents, 18. Sodium and potassium salts of caffeic acid tetramers from Arnebia euchroma as anti-HIV agents. *J Nat Prod*, **58**, 392-400.
570. Kashman, Y., Gustafson, K.R., Fuller, R.W. *et al.* (1992) The calanolides, a novel HIV-inhibitory class of coumarin derivatives from the tropical rainforest tree, Calophyllum lanigerum. *J Med Chem*, **35**, 2735-2743.
571. Katavic, P.L., Venables, D.A., Rali, T. *et al.* (2007) Indolizidine alkaloids with delta-opioid receptor binding affinity from the leaves of Elaeocarpus fuscoides. *J Nat Prod*, **70**, 872-875.
572. Kato, A., Asano, N., Kizu, H. *et al.* (1997) Fagomine isomers and glycosides from Xanthocercis zambesiaca. *J Nat Prod*, **60**, 312-314.
573. Kato, A., Kato, N., Adachi, I. *et al.* (2007) Isolation of glycosidase-inhibiting hyacinthacines and related alkaloids from Scilla socialis. *J Nat Prod*, **70**, 993-997.
574. Kato, T., Saito, N., Kashimura, K. *et al.* (2002) Germination and growth inhibitors from wheat (Triticum aestivum L.) husks. *J Agric Food Chem*, **50**, 6307-6312.
575. Kaur, K., Jain, M., Kaur, T. *et al.* (2009) Antimalarials from nature. *Bioorg Med Chem*, **17**, 3229-3256.
576. Kawakami, S., Harinantenaina, L., Matsunami, K. *et al.* (2008) Macaflavanones A-G, prenylated flavanones from the leaves of Macaranga tanarius. *J Nat Prod*, **71**, 1872-1876.
577. Kawazoe, K., Tsubouchi, Y., Abdullah, N. *et al.* (2003) Sesquiterpenoids from Artemisia gilvescens and an anti-MRSA compound. *J Nat Prod*, **66**, 538-539.
578. Kawazoe, K., Yutani, A., Tamemoto, K. *et al.* (2001) Phenylnaphthalene compounds from the subterranean part of Vitex rotundifolia and their antibacterial activity against methicillin-resistant Staphylococcus aureus. *J Nat Prod*, **64**, 588-591.
579. Kendziorek, M. and Paszkowski, A. (2008) Properties of serine:glyoxylate aminotransferase purified from Arabidopsis thaliana leaves. *Acta Biochim Biophys Sin (Shanghai)*, **40**, 102-110.
580. Kernan, M.R., Sendl, A., Chen, J.L. *et al.* (1997) Two new lignans with activity against influenza virus from the medicinal plant Rhinacanthus nasutus. *J Nat Prod*, **60**, 635-637.
581. Khan, I.A., Avery, M.A., Burandt, C.L. *et al.* (2000) Antigiardial activity of isoflavones from Dalbergia frutescens bark. *J Nat Prod*, **63**, 1414-1416.
582. Khera, S., Woldemichael, G.M., Singh, M.P. *et al.* (2003) A novel antibacterial iridoid and triterpene from Caiophora coronata. *J Nat Prod*, **66**, 1628-1631.
583. Kigodi, P.G., Blasko, G., Thebtaranonth, Y. *et al.* (1989) Spectroscopic and biological investigation of nimbolide and 28-deoxonimbolide from Azadirachta indica. *J Nat Prod*, **52**, 1246-1251.
584. Kikuchi, H., Ohtsuki, T., Koyano, T. *et al.* (2007) Brandisianins A-F, isoflavonoids isolated from Millettia brandisiana in a screening program for death-receptor expression enhancement activity. *J Nat Prod*, **70**, 1910-1914.

585. Kikuchi, H., Ohtsuki, T., Koyano, T. *et al.* (2009) Death receptor 5 targeting activity-guided isolation of isoflavones from Millettia brandisiana and Ardisia colorata and evaluation of ability to induce TRAIL-mediated apoptosis. *Bioorg Med Chem*, **17**, 1181-1186.
586. Kikuchi, T., Ishii, K., Noto, T. *et al.* (2011) Cytotoxic and apoptosis-inducing activities of limonoids from the seeds of Azadirachta indica (neem). *J Nat Prod*, **74**, 866-870.
587. Kil, Y.S., Kim, S.M., Kang, U. *et al.* (2017) Peroxynitrite-Scavenging Glycosides from the Stem Bark of Catalpa ovata. *J Nat Prod*, **80**, 2240-2251.
588. Kilic, O., Kocak, A. and Bagci, E. (2011) Composition of the volatile oils of two Anthemis L. taxa from Turkey. *Z Naturforsch C*, **66**, 535-540.
589. Killday, K.B., Davey, M.H., Glinski, J.A. *et al.* (2011) Bioactive A-type proanthocyanidins from Cinnamomum cassia. *J Nat Prod*, **74**, 1833-1841.
590. Kim, C.S., Subedi, L., Kim, S.Y. *et al.* (2015) Lignan Glycosides from the Twigs of Chaenomeles sinensis and Their Biological Activities. *J Nat Prod*, **78**, 1174-1178.
591. Kim, D.C., Choi, S.Y., Kim, S.H. *et al.* (2006) Isoliquiritigenin selectively inhibits H(2) histamine receptor signaling. *Mol Pharmacol*, **70**, 493-500.
592. Kim, D.K., Lim, J.P., Yang, J.H. *et al.* (2002) Acetylcholinesterase inhibitors from the roots of Angelica dahurica. *Arch Pharm Res*, **25**, 856-859.
593. Kim, H.J., Kim, E.J., Seo, S.H. *et al.* (2006) Vanillic acid glycoside and quinic acid derivatives from Gardeniae Fructus. *J Nat Prod*, **69**, 600-603.
594. Kim, H.M., Kim, S.J., Kim, H.Y. *et al.* (2015) Constituents of the stem barks of Ailanthus altissima and their potential to inhibit LPS-induced nitric oxide production. *Bioorg Med Chem Lett*, **25**, 1017-1020.
595. Kim, J.H., Ryu, Y.B., Lee, W.S. *et al.* (2014) Neuraminidase inhibitory activities of quaternary isoquinoline alkaloids from Corydalis turtschaninovii rhizome. *Bioorg Med Chem*, **22**, 6047-6052.
596. Kim, J.Y., Lim, H.J. and Ryu, J.H. (2008) In vitro anti-inflammatory activity of 3-O-methyl-flavones isolated from Siegesbeckia glabrescens. *Bioorg Med Chem Lett*, **18**, 1511-1514.
597. Kim, K.H., Choi, J.W., Ha, S.K. *et al.* (2010) Neolignans from Piper kadsura and their anti-neuroinflammatory activity. *Bioorg Med Chem Lett*, **20**, 409-412.
598. Kim, K.H., Choi, S.U., Ha, S.K. *et al.* (2009) Biphenyls from Berberis koreana. *J Nat Prod*, **72**, 2061-2064.
599. Kim, K.H., Choi, S.U., Kim, Y.C. *et al.* (2011) Tirucallane triterpenoids from Cornus walteri. *J Nat Prod*, **74**, 54-59.
600. Kim, K.H., Kim, C.S., Park, Y.J. *et al.* (2015) Anti-inflammatory and antitumor phenylpropanoid sucrosides from the seeds of Raphanus sativus. *Bioorg Med Chem Lett*, **25**, 96-99.
601. Kim, K.H., Kim, H.K., Choi, S.U. *et al.* (2011) Bioactive lignans from the rhizomes of Acorus gramineus. *J Nat Prod*, **74**, 2187-2192.
602. Kim, K.H., Kim, M.A., Moon, E. *et al.* (2011) Furostanol saponins from the rhizomes of Dioscorea japonica and their effects on NGF induction. *Bioorg Med Chem Lett*, **21**, 2075-2078.
603. Kim, K.H., Lee, I.K., Piao, C.J. *et al.* (2010) Benzylisoquinoline alkaloids from the tubers of Corydalis ternata and their cytotoxicity. *Bioorg Med Chem Lett*, **20**, 4487-4490.
604. Kim, K.H., Moon, E., Choi, S.U. *et al.* (2011) Biological evaluation of phenolic constituents from the trunk of Berberis koreana. *Bioorg Med Chem Lett*, **21**, 2270-2273.
605. Kim, K.H., Moon, E., Kim, H.K. *et al.* (2012) Phenolic constituents from the rhizomes of Acorus gramineus and their biological evaluation on antitumor and anti-inflammatory activities. *Bioorg Med Chem Lett*, **22**, 6155-6159.
606. Kim, M.K., Choi, G.J. and Lee, H.S. (2003) Fungicidal property of Curcuma longa L. rhizome-derived curcumin against phytopathogenic fungi in a greenhouse. *J Agric Food Chem*, **51**, 1578-1581.
607. Kim, S.B., Ahn, J.H., Han, S.B. *et al.* (2012) Anti-adipogenic chromone glycosides from Cnidium monnieri fruits in 3T3-L1 cells. *Bioorg Med Chem Lett*, **22**, 6267-6271.
608. Kim, S.E., Kim, H.S., Hong, Y.S. *et al.* (1999) Sesquiterpene esters from Celastrus orbiculatus and their structure-activity relationship on the modulation of multidrug resistance. *J Nat Prod*, **62**, 697-700.
609. Kim, S.E., Kim, Y.H., Lee, J.J. *et al.* (1998) A new sesquiterpene ester from Celastrus orbiculatus reversing multidrug resistance in cancer cells. *J Nat Prod*, **61**, 108-111.
610. Kim, S.H., Park, J.H., Kim, T.B. *et al.* (2010) Inhibition of antigen-induced degranulation by aryl compounds isolated from the bark of Betula platyphylla in RBL-2H3 cells. *Bioorg Med Chem Lett*, **20**, 2824-2827.
611. Kim, S.J., Moon, Y.J. and Lee, S.M. (2010) Protective effects of baicalin against ischemia/reperfusion injury in rat liver. *J Nat Prod*, **73**, 2003-2008.
612. Kim, S.R., Lee, K.Y., Koo, K.A. *et al.* (2002) Four new neuroprotective iridoid glycosides from Scrophularia buergeriana roots. *J Nat Prod*, **65**, 1696-1699.

613. Kim, S.Y., Choi, Y.H., Huh, H. *et al.* (1997) New antihepatotoxic cerebroside from Lycium chinense fruits. *J Nat Prod*, **60**, 274-276.
614. Kim, Y., Bang, S.C., Lee, J.H. *et al.* (2004) Pulsatilla saponin D: the antitumor principle from Pulsatilla koreana. *Arch Pharm Res*, **27**, 915-918.
615. Kim, Y.A., Kong, C.S., Lee, J.I. *et al.* (2012) Evaluation of novel antioxidant triterpenoid saponins from the halophyte Salicornia herbacea. *Bioorg Med Chem Lett*, **22**, 4318-4322.
616. Kim, Y.B., Thwe, A.A., Li, X. *et al.* (2014) Accumulation of astragalosides and related gene expression in different organs of Astragalus membranaceus Bge. var mongholicus (Bge.). *Molecules*, **19**, 10922-10935.
617. Kingston, D.G., Hawkins, D.R. and Ovington, L. (1982) New taxanes from Taxus brevifolia. *J Nat Prod*, **45**, 466-470.
618. Kishi, A., Morikawa, T., Matsuda, H. *et al.* (2003) Structures of new friedelane- and norfriedelane-type triterpenes and polyacylated eudesmane-type sesquiterpene from Salacia chinensis LINN. (S. prinoides DC., Hippocrateaceae) and radical scavenging activities of principal constituents. *Chem Pharm Bull (Tokyo)*, **51**, 1051-1055.
619. Kitagawa, I., Mahmud, T., Yokota, K. *et al.* (1996) Indonesian medicinal plants. XVII. Characterization of quassinoids from the stems of Quassia indica. *Chem Pharm Bull (Tokyo)*, **44**, 2009-2014.
620. Kitajima, M., Nakamura, T., Kogure, N. *et al.* (2006) Isolation of gelsedine-type indole alkaloids from Gelsemium elegans and evaluation of the cytotoxic activity of gelsemium alkaloids for A431 epidermoid carcinoma cells. *J Nat Prod*, **69**, 715-718.
621. Kittakoop, P., Wanasith, S., Watts, P. *et al.* (2001) Potent antiviral potamogetonyde and potamogetonol, new furanoid labdane diterpenes from Potamogeton malaianus. *J Nat Prod*, **64**, 385-388.
622. Kiuchi, F., Matsuo, K., Ito, M. *et al.* (2004) New norditerpenoids with trypanocidal activity from Vitex trifolia. *Chem Pharm Bull (Tokyo)*, **52**, 1492-1494.
623. Kjer, J., Wray, V., Edrada-Ebel, R. *et al.* (2009) Xanalteric acids I and II and related phenolic compounds from an endophytic Alternaria sp. isolated from the mangrove plant Sonneratia alba. *J Nat Prod*, **72**, 2053-2057.
624. Klausmeyer, P., Zhou, Q., Scudiero, D.A. *et al.* (2009) Cytotoxic and HIF-1alpha inhibitory compounds from Crossosoma bigelovii. *J Nat Prod*, **72**, 805-812.
625. Klinghammer, M. and Tenhaken, R. (2007) Genome-wide analysis of the UDP-glucose dehydrogenase gene family in Arabidopsis, a key enzyme for matrix polysaccharides in cell walls. *J Exp Bot*, **58**, 3609-3621.
626. Ko, H.H., Chang, W.L. and Lu, T.M. (2008) Antityrosinase and antioxidant effects of ent-kaurane diterpenes from leaves of Broussonetia papyrifera. *J Nat Prod*, **71**, 1930-1933.
627. Ko, H.H., Weng, J.R., Tsao, L.T. *et al.* (2004) Anti-inflammatory flavonoids and pterocarpanoid from Crotalaria pallida and C. assamica. *Bioorg Med Chem Lett*, **14**, 1011-1014.
628. Ko, H.H., Yen, M.H., Wu, R.R. *et al.* (1999) Cytotoxic isoprenylated flavans of Broussonetia kazinoki. *J Nat Prod*, **62**, 164-166.
629. Ko, H.H., Yu, S.M., Ko, F.N. *et al.* (1997) Bioactive constituents of Morus australis and Broussonetia papyrifera. *J Nat Prod*, **60**, 1008-1011.
630. Ko, W.G., Kang, T.H., Lee, S.J. *et al.* (2001) Rotundifuran, a labdane type diterpene from Vitex rotundifolia, induces apoptosis in human myeloid leukaemia cells. *Phytother Res*, **15**, 535-537.
631. Komoda, Y., Chujo, H., Ishihara, S. *et al.* (1989) HPLC quantitative analysis of plantaginin in Shazenso (Plantago asiatica L.) extracts and isolation of plantamajoside. *Tokyo Ika Shika Daigaku Iyo Kizai Kenkyusho Hokoku*, **23**, 81-85.
632. Kong, D.Y., Luo, S.Q., Li, H.T. *et al.* (1988) [Studies on the chemical components of Viscum coloratum. III. Structure of viscumneoside III, V and VI]. *Yao Xue Xue Bao*, **23**, 593-600.
633. Kongkiatpaiboon, S., Schinnerl, J., Felsinger, S. *et al.* (2011) Structural relationships of stemona alkaloids: assessment of species-specific accumulation trends for exploiting their biological activities. *J Nat Prod*, **74**, 1931-1938.
634. Konoshima, T. and Lee, K.H. (1986) Antitumor agents, 82. Cytotoxic sapogenols from Aesculus hippocastanum. *J Nat Prod*, **49**, 650-656.
635. Konoshima, T., Okamoto, E., Kozuka, M. *et al.* (1988) Studies on inhibitors of skin tumor promotion, III. Inhibitory effects of isoflavonoids from Wisteria brachybotrys on Epstein-Barr virus activation. *J Nat Prod*, **51**, 1266-1270.
636. Konoshima, T., Takasaki, M., Kozuka, M. *et al.* (1987) Studies on inhibitors of skin-tumor promotion, I. Inhibitory effects of triterpenes from Euptelea polyandra on Epstein-Barr virus activation. *J Nat Prod*, **50**, 1167-1170.
637. Konoshima, T., Terada, H., Kokumai, M. *et al.* (1993) Studies on inhibitors of skin tumor promotion, XII. Rotenoids from Amorpha fruticosa. *J Nat Prod*, **56**, 843-848.

638. Koo, H.J., Park, H.J., Byeon, H.E. *et al.* (2014) Chinese yam extracts containing beta-sitosterol and ethyl linoleate protect against atherosclerosis in apolipoprotein E-deficient mice and inhibit muscular expression of VCAM-1 in vitro. *J Food Sci*, **79**, H719-729.
639. Kouam, S.F., Khan, S.N., Krohn, K. *et al.* (2006) Alpha-glucosidase inhibitory anthranols, kenganthranols A-C, from the stem bark of Harungana madagascariensis. *J Nat Prod*, **69**, 229-233.
640. Kouam, S.F., Yapna, D.B., Krohn, K. *et al.* (2007) Antimicrobial prenylated anthracene derivatives from the leaves of Harungana madagascariensis. *J Nat Prod*, **70**, 600-603.
641. Kougan, G.B., Miyamoto, T., Tanaka, C. *et al.* (2010) Steroidal saponins from two species of Dracaena. *J Nat Prod*, **73**, 1266-1270.
642. Koyama, K., Hirasawa, Y., Zaima, K. *et al.* (2008) Alstilobanines A-E, new indole alkaloids from Alstonia angustiloba. *Bioorg Med Chem*, **16**, 6483-6488.
643. Kraft, C., Jenett-Siems, K., Siems, K. *et al.* (2000) Antiplasmodial activity of isoflavones from Andira inermis. *J Ethnopharmacol*, **73**, 131-135.
644. Kramell, R., Schmidt, J., Herrmann, G. *et al.* (2005) N-(jasmonoyl)tyrosine-derived compounds from flowers of broad beans (Vicia faba). *J Nat Prod*, **68**, 1345-1349.
645. Kretschmer, N., Rinner, B., Deutsch, A.J. *et al.* (2012) Naphthoquinones from Onosma paniculata induce cell-cycle arrest and apoptosis in melanoma Cells. *J Nat Prod*, **75**, 865-869.
646. Kuang, X., Li, W., Kanno, Y. *et al.* (2014) Cycloartane-type triterpenes from Euphorbia fischeriana stimulate human CYP3A4 promoter activity. *Bioorg Med Chem Lett*, **24**, 5423-5427.
647. Kuchta, K., Ortwein, J., Hennig, L. *et al.* (2014) (1)H-qNMR for direct quantification of stachydrine in Leonurus japonicus and L. cardiaca. *Fitoterapia*, **96**, 8-17.
648. Kueh, J.S., Mackenzie, I.A. and Pattenden, G. (1985) Production of chrysanthemic acid and pyrethrins by tissue cultures ofChrysanthemum cinerariaefolium. *Plant Cell Rep*, **4**, 118-119.
649. Kuo, Y.C., Yang, N.S., Chou, C.J. *et al.* (2000) Regulation of cell proliferation, gene expression, production of cytokines, and cell cycle progression in primary human T lymphocytes by piperlactam S isolated from Piper kadsura. *Mol Pharmacol*, **58**, 1057-1066.
650. Kuo, Y.H., Hsu, Y.W., Liaw, C.C. *et al.* (2005) Cytotoxic phenylpropanoid glycosides from the stems of Smilax china. *J Nat Prod*, **68**, 1475-1478.
651. Kuo, Y.H., Huang, H.C., Chiou, W.F. *et al.* (2003) A novel NO-production-inhibiting triterpene and cytotoxicity of known alkaloids from Euonymus laxiflorus. *J Nat Prod*, **66**, 554-557.
652. Kuo, Y.J., Yang, Y.C., Zhang, L.J. *et al.* (2010) Flavanone and diphenylpropane glycosides and glycosidic acyl esters from Viscum articulatum. *J Nat Prod*, **73**, 109-114.
653. Kuroda, M., Mimaki, Y., Honda, S. *et al.* (2010) Phenolics from Glycyrrhiza glabra roots and their PPAR-gamma ligand-binding activity. *Bioorg Med Chem*, **18**, 962-970.
654. Kuroda, M., Mimaki, Y., Sakagami, H. *et al.* (2003) Bulbinelonesides A-E, phenylanthraquinone glycosides from the roots of Bulbinella floribunda. *J Nat Prod*, **66**, 894-897.
655. Kurokawa, M., Basnet, P., Ohsugi, M. *et al.* (1999) Anti-herpes simplex virus activity of moronic acid purified from Rhus javanica in vitro and in vivo. *J Pharmacol Exp Ther*, **289**, 72-78.
656. Kusano, R., Ogawa, S., Matsuo, Y. *et al.* (2011) alpha-Amylase and lipase inhibitory activity and structural characterization of acacia bark proanthocyanidins. *J Nat Prod*, **74**, 119-128.
657. Kusari, S., Zuhlke, S. and Spiteller, M. (2011) Effect of artificial reconstitution of the interaction between the plant Camptotheca acuminata and the fungal endophyte Fusarium solani on camptothecin biosynthesis. *J Nat Prod*, **74**, 764-775.
658. Kwon, H.S., Kim, M.J., Jeong, H.J. *et al.* (2008) Low-density lipoprotein (LDL)-antioxidant lignans from Myristica fragrans seeds. *Bioorg Med Chem Lett*, **18**, 194-198.
659. Kwon, J., Basnet, S., Lee, J.W. *et al.* (2015) Chemical constituents isolated from the Mongolian medicinal plant Sophora alopecuroides L. and their inhibitory effects on LPS-induced nitric oxide production in RAW 264.7 macrophages. *Bioorg Med Chem Lett*, **25**, 3314-3318.
660. Kwon, S.H., Kim, H.C., Lee, S.Y. *et al.* (2009) Loganin improves learning and memory impairments induced by scopolamine in mice. *Eur J Pharmacol*, **619**, 44-49.
661. Lacroix, D., Prado, S., Kamoga, D. *et al.* (2011) Structure and in vitro antiparasitic activity of constituents of Citropsis articulata root bark. *J Nat Prod*, **74**, 2286-2289.
662. Lai, W., Wu, Z., Lin, H. *et al.* (2010) Anti-ischemia steroidal saponins from the seeds of Allium fistulosum. *J Nat Prod*, **73**, 1053-1057.
663. Lai, W.C., Wang, H.C., Chen, G.Y. *et al.* (2011) Using the pER8:GUS reporter system to screen for phytoestrogens from Caesalpinia sappan. *J Nat Prod*, **74**, 1698-1706.
664. Lai, Y., Liu, T., Sa, R. *et al.* (2015) Neolignans with a Rare 2-Oxaspiro[4.5]deca-6,9-dien-8-one Motif from the Stem Bark of Cinnamomum subavenium. *J Nat Prod*, **78**, 1740-1744.
665. Lam, S.H., Ruan, C.T., Hsieh, P.H. *et al.* (2012) Hypoglycemic diterpenoids from Tinospora crispa. *J Nat Prod*, **75**, 153-159.

666. Lan, Y.H., Chang, F.R., Yu, J.H. *et al.* (2003) Cytotoxic styrylpyrones from Goniothalamus amuyon. *J Nat Prod*, **66**, 487-490.
667. Langat, M.K., Crouch, N.R., Smith, P.J. *et al.* (2011) Cembranolides from the leaves of Croton gratissimus. *J Nat Prod*, **74**, 2349-2355.
668. Laphookhieo, S., Karalai, C., Ponglimanont, C. *et al.* (2004) Pentacyclic triterpenoid esters from the fruits of Bruguiera cylindrica. *J Nat Prod*, **67**, 886-888.
669. Lasswell, W.L., Jr. and Hufford, C.D. (1977) Cytotoxic C-benzylated flavonoids from Uvaria chamae. *J Org Chem*, **42**, 1295-1302.
670. Latha, C. (2007) Microwave-assisted extraction of embelin from Embelia ribes. *Biotechnol Lett*, **29**, 319-322.
671. Lee, A.R., Wu, W.L., Chang, W.L. *et al.* (1987) Isolation and bioactivity of new tanshinones. *J Nat Prod*, **50**, 157-160.
672. Lee, B.W., Gal, S.W., Park, K.M. *et al.* (2005) Cytotoxic xanthones from Cudrania tricuspidata. *J Nat Prod*, **68**, 456-458.
673. Lee, B.W., Lee, J.H., Lee, S.T. *et al.* (2005) Antioxidant and cytotoxic activities of xanthones from Cudrania tricuspidata. *Bioorg Med Chem Lett*, **15**, 5548-5552.
674. Lee, C., Kim, H. and Kho, Y. (2002) Agastinol and agastenol, novel lignans from Agastache rugosa and their evaluation in an apoptosis inhibition assay. *J Nat Prod*, **65**, 414-416.
675. Lee, C., Lee, J.W., Jin, Q. *et al.* (2013) Inhibitory constituents of the heartwood of Dalbergia odorifera on nitric oxide production in RAW 264.7 macrophages. *Bioorg Med Chem Lett*, **23**, 4263-4266.
676. Lee, C., Lee, J.W., Jin, Q. *et al.* (2015) Isolation and Characterization of Dammarane-Type Saponins from Gynostemma pentaphyllum and Their Inhibitory Effects on IL-6-Induced STAT3 Activation. *J Nat Prod*, **78**, 971-976.
677. Lee, C., Lee, J.W., Jin, Q. *et al.* (2013) Anti-inflammatory constituents from the fruits of Vitex rotundifolia. *Bioorg Med Chem Lett*, **23**, 6010-6014.
678. Lee, C.L., Chang, F.R., Yen, M.H. *et al.* (2009) Cytotoxic phenanthrenequinones and 9,10-dihydrophenanthrenes from Calanthe arisanensis. *J Nat Prod*, **72**, 210-213.
679. Lee, C.L., Chiang, L.C., Cheng, L.H. *et al.* (2009) Influenza A (H(1)N(1)) Antiviral and Cytotoxic Agents from Ferula assa-foetida. *J Nat Prod*, **72**, 1568-1572.
680. Lee, C.L., Liao, Y.C., Hwang, T.L. *et al.* (2010) Ixorapeptide I and ixorapeptide II, bioactive peptides isolated from Ixora coccinea. *Bioorg Med Chem Lett*, **20**, 7354-7357.
681. Lee, C.L., Nakagawa-Goto, K., Yu, D. *et al.* (2008) Cytotoxic calanquinone A from Calanthe arisanensis and its first total synthesis. *Bioorg Med Chem Lett*, **18**, 4275-4277.
682. Lee, D., Bhat, K.P., Fong, H.H. *et al.* (2001) Aromatase inhibitors from Broussonetia papyrifera. *J Nat Prod*, **64**, 1286-1293.
683. Lee, D.G., Jung, H.J. and Woo, E.R. (2005) Antimicrobial property of (+)-lyoniresinol-3alpha-O-beta-D-glucopyranoside isolated from the root bark of Lycium chinense Miller against human pathogenic microorganisms. *Arch Pharm Res*, **28**, 1031-1036.
684. Lee, D.Y., Kim, D.H., Lee, H.J. *et al.* (2010) New estrogenic compounds isolated from Broussonetia kazinoki. *Bioorg Med Chem Lett*, **20**, 3764-3767.
685. Lee, G.Y., Kim, J.H., Choi, S.K. *et al.* (2015) Constituents of the seeds of Cassia tora with inhibitory activity on soluble expoxide hydrolease. *Bioorg Med Chem Lett*, **25**, 5097-5101.
686. Lee, H., Kim, Y., Choi, I. *et al.* (2010) Two novel neo-clerodane diterpenoids from Scutellaria barbata. *Bioorg Med Chem Lett*, **20**, 288-290.
687. Lee, H.E., Jeon, S.J., Ryu, B. *et al.* (2016) Swertisin, a C-glucosylflavone, ameliorates scopolamine-induced memory impairment in mice with its adenosine A1 receptor antagonistic property. *Behav Brain Res*, **306**, 137-145.
688. Lee, J., Lee, Y.M., Lee, B.W. *et al.* (2012) Chemical constituents from the aerial parts of Aster koraiensis with protein glycation and aldose reductase inhibitory activities. *J Nat Prod*, **75**, 267-270.
689. Lee, J.E., Hitotsuyanagi, Y., Kim, I.H. *et al.* (2008) A novel bicyclic hexapeptide, RA-XVIII, from Rubia cordifolia: structure, semi-synthesis, and cytotoxicity. *Bioorg Med Chem Lett*, **18**, 808-811.
690. Lee, J.S., Kim, H.J., Park, H. *et al.* (2002) New diarylheptanoids from the stems of Carpinus cordata. *J Nat Prod*, **65**, 1367-1370.
691. Lee, J.S., Kim, J., Kim, B.Y. *et al.* (2000) Inhibition of phospholipase cgamma1 and cancer cell proliferation by triterpene esters from Uncaria rhynchophylla. *J Nat Prod*, **63**, 753-756.
692. Lee, J.W., Lee, C., Jin, Q. *et al.* (2014) Pyranocoumarins from Glehnia littoralis inhibit the LPS-induced NO production in macrophage RAW 264.7 cells. *Bioorg Med Chem Lett*, **24**, 2717-2719.
693. Lee, K.K., Bahler, B.D., Hofmann, G.A. *et al.* (1998) Isolation and structure elucidation of new PKCalpha inhibitors from Pinus flexilis. *J Nat Prod*, **61**, 1407-1409.

694. Lee, K.Y., Sung, S.H. and Kim, Y.C. (2006) Neuroprotective bibenzyl glycosides of Stemona tuberosa roots. *J Nat Prod*, **69**, 679-681.
695. Lee, M., Lee, H.H., Lee, J.K. *et al.* (2013) Anti-adipogenic activity of compounds isolated from Idesia polycarpa on 3T3-L1 cells. *Bioorg Med Chem Lett*, **23**, 3170-3174.
696. Lee, M., Lee, M.K., Kim, Y.C. *et al.* (2011) Antifibrotic constituents of Alnus firma on hepatic stellate cells. *Bioorg Med Chem Lett*, **21**, 2906-2910.
697. Lee, M.K., Lim, S.W., Yang, H. *et al.* (2006) Osteoblast differentiation stimulating activity of biflavonoids from Cephalotaxus koreana. *Bioorg Med Chem Lett*, **16**, 2850-2854.
698. Lee, S., Song, I.H., Lee, J.H. *et al.* (2014) Sortase A inhibitory metabolites from the roots of Pulsatilla koreana. *Bioorg Med Chem Lett*, **24**, 44-48.
699. Lee, S.H., Bae, E.A., Park, E.K. *et al.* (2007) Inhibitory effect of eupatilin and jaceosidin isolated from Artemisia princeps in IgE-induced hypersensitivity. *Int Immunopharmacol*, **7**, 1678-1684.
700. Lee, T.H., Qiu, F., Waller, G.R. *et al.* (2000) Three new flavonol galloylglycosides from leaves of Acacia confusa. *J Nat Prod*, **63**, 710-712.
701. Lee, T.H., Wang, M.J., Chen, P.Y. *et al.* (2009) Constituents of Polyalthia longifolia var. pendula. *J Nat Prod*, **72**, 1960-1963.
702. Lee, W.C., Jung, H.A., Choi, J.S. *et al.* (2011) Protective effects of luteolin against apoptotic liver damage induced by D-galactosamine/lipopolysaccharide in mice. *J Nat Prod*, **74**, 1916-1921.
703. Lee, W.S., Baek, Y.I., Kim, J.R. *et al.* (2004) Antioxidant activities of a new lignan and a neolignan from Saururus chinensis. *Bioorg Med Chem Lett*, **14**, 5623-5628.
704. Lee, W.Y., Cheung, C.C., Liu, K.W. *et al.* (2010) Cytotoxic effects of tanshinones from Salvia miltiorrhiza on doxorubicin-resistant human liver cancer cells. *J Nat Prod*, **73**, 854-859.
705. Lee, Y.S., Ju, H.K., Kim, Y.J. *et al.* (2013) Enhancement of anti-inflammatory activity of Aloe vera adventitious root extracts through the alteration of primary and secondary metabolites via salicylic acid elicitation. *PLoS One*, **8**, e82479.
706. Lee, Y.S., Kim, S.H., Kim, J.K. *et al.* (2011) Preparative isolation and purification of seven isoflavones from Belamcanda chinensis. *Phytochem Anal*, **22**, 468-473.
707. Lei, Y., Tan, J., Wink, M. *et al.* (2013) An isoquinoline alkaloid from the Chinese herbal plant Corydalis yanhusuo W.T. Wang inhibits P-glycoprotein and multidrug resistance-associate protein 1. *Food Chem*, **136**, 1117-1121.
708. Lenta, B.N., Vonthron-Senecheau, C., Weniger, B. *et al.* (2007) Leishmanicidal and cholinesterase inhibiting activities of phenolic compounds from Allanblackia monticola and Symphonia globulifera. *Molecules*, **12**, 1548-1557.
709. Lhinhatrakool, T. and Sutthivaiyakit, S. (2006) 19-Nor- and 18,20-epoxy-cardenolides from the leaves of Calotropis gigantea. *J Nat Prod*, **69**, 1249-1251.
710. Li, A.P., Li, Z.Y., Sun, H.F. *et al.* (2015) Comparison of Two Different Astragali Radix by a (1)H NMR-Based Metabolomic Approach. *J Proteome Res*, **14**, 2005-2016.
711. Li, B., Ni, Y., Zhu, L.J. *et al.* (2015) Flavonoids from Matteuccia struthiopteris and Their Anti-influenza Virus (H1N1) Activity. *J Nat Prod*, **78**, 987-995.
712. Li, D.Q., Wu, J., Liu, L.Y. *et al.* (2015) Cytotoxic triterpenoid glycosides (saikosaponins) from the roots of Bupleurum chinense. *Bioorg Med Chem Lett*, **25**, 3887-3892.
713. Li, D.X., Liu, M. and Zhou, X.J. (2015) A new dimeric lignan from Zanthoxylum simulans. *Zhongguo Zhong Yao Za Zhi*, **40**, 2843-2848.
714. Li, F., Sun, Q., Hong, L. *et al.* (2013) Daphnane-type diterpenes with inhibitory activities against human cancer cell lines from Daphne genkwa. *Bioorg Med Chem Lett*, **23**, 2500-2504.
715. Li, H., Wang, L., Miyata, S. *et al.* (2008) Kadsuracoccinic acids A-C, ring-A seco-lanostane triterpenes from Kadsura coccinea and their effects on embryonic cell division of Xenopus laevis. *J Nat Prod*, **71**, 739-741.
716. Li, H., Wang, L., Yang, Z. *et al.* (2007) Kadsuralignans H-K from Kadsura coccinea and their nitric oxide production inhibitory effects. *J Nat Prod*, **70**, 1999-2002.
717. Li, J., Baroja-Fernandez, E., Bahaji, A. *et al.* (2013) Enhancing sucrose synthase activity results in increased levels of starch and ADP-glucose in maize (Zea mays L.) seed endosperms. *Plant Cell Physiol*, **54**, 282-294.
718. Li, J., Ding, Y., Li, X.C. *et al.* (2009) Scuteflorins A and B, dihydropyranocoumarins from Scutellaria lateriflora. *J Nat Prod*, **72**, 983-987.
719. Li, J., Li, M.Y., Feng, G. *et al.* (2012) Moluccensins R-Y, limonoids from the seeds of a mangrove, Xylocarpus moluccensis. *J Nat Prod*, **75**, 1277-1283.
720. Li, J., Liao, C.R., Wei, J.Q. *et al.* (2011) Diarylheptanoids from Curcuma kwangsiensis and their inhibitory activity on nitric oxide production in lipopolysaccharide-activated macrophages. *Bioorg Med Chem Lett*, **21**, 5363-5369.

721. Li, J., Mahdi, F., Du, L. *et al.* (2013) Semisynthetic studies identify mitochondria poisons from botanical dietary supplements--geranyloxycoumarins from Aegle marmelos. *Bioorg Med Chem*, **21**, 1795-1803.
722. Li, J., Meng, A.P., Guan, X.L. *et al.* (2013) Anti-hepatitis B virus lignans from the root of Streblus asper. *Bioorg Med Chem Lett*, **23**, 2238-2244.
723. Li, J.L., Gao, L.X., Meng, F.W. *et al.* (2015) PTP1B inhibitors from stems of Angelica keiskei (Ashitaba). *Bioorg Med Chem Lett*, **25**, 2028-2032.
724. Li, J.Z., Qing, C., Chen, C.X. *et al.* (2009) Cytotoxicity of cardenolides and cardenolide glycosides from Asclepias curassavica. *Bioorg Med Chem Lett*, **19**, 1956-1959.
725. Li, L., Wang, H.K., Chang, J.J. *et al.* (1993) Antitumor agents, 138. Rotenoids and isoflavones as cytotoxic constitutents from Amorpha fruticosa. *J Nat Prod*, **56**, 690-698.
726. Li, L.M., Li, G.Y., Ding, L.S. *et al.* (2008) ent-Kaurane Diterpenoids from Isodon nervosus. *J Nat Prod*, **71**, 684-688.
727. Li, L.M., Weng, Z.Y., Huang, S.X. *et al.* (2007) Cytotoxic ent-kauranoids from the medicinal plant Isodon xerophilus. *J Nat Prod*, **70**, 1295-1301.
728. Li, N., Di, L., Gao, W.C. *et al.* (2012) Cytotoxic iridoids from the roots of Patrinia scabra. *J Nat Prod*, **75**, 1723-1728.
729. Li, N., Wu, C.F., Xu, X.Y. *et al.* (2012) Triterpenes possessing an unprecedented skeleton isolated from hydrolyzate of total saponins from Gynostemma pentaphyllum. *Eur J Med Chem*, **50**, 173-178.
730. Li, Q., Li, W., Hui, L.P. *et al.* (2012) 13,28-Epoxy triterpenoid saponins from Ardisia japonica selectively inhibit proliferation of liver cancer cells without affecting normal liver cells. *Bioorg Med Chem Lett*, **22**, 6120-6125.
731. Li, S.F., Zhang, Y., Li, Y. *et al.* (2012) beta-Carboline alkaloids from the leaves of Trigonostemon lii Y.T. Chang. *Bioorg Med Chem Lett*, **22**, 2296-2299.
732. Li, S.S., Gao, Z., Feng, X. *et al.* (2004) Biscoumarin derivatives from Edgeworthia gardneri that inhibit the lyase activity of DNA polymerase beta. *J Nat Prod*, **67**, 1608-1610.
733. Li, W., Jiang, Z., Shen, L. *et al.* (2015) Antiviral Limonoids Including Khayanolides from the Trang Mangrove Plant Xylocarpus moluccensis. *J Nat Prod*, **78**, 1570-1578.
734. Li, W.X., Cui, C.B., Cai, B. *et al.* (2005) Labdane-type diterpenes as new cell cycle inhibitors and apoptosis inducers from Vitex trifolia L. *J Asian Nat Prod Res*, **7**, 95-105.
735. Li, X., Zhang, S.D., Jin, H.Z. *et al.* (2013) A new flavonol from Oxytropis ochrocephala Bunge. *Nat Prod Res*, **27**, 554-557.
736. Li, X.C., Jacob, M.R., Pasco, D.S. *et al.* (2001) Phenolic compounds from Miconia myriantha inhibiting Candida aspartic proteases. *J Nat Prod*, **64**, 1282-1285.
737. Li, X.C., Joshi, A.S., ElSohly, H.N. *et al.* (2002) Fatty acid synthase inhibitors from plants: isolation, structure elucidation, and SAR studies. *J Nat Prod*, **65**, 1909-1914.
738. Li, X.H. and McLaughlin, J.L. (1989) Bioactive compounds from the root of Myrsine africana. *J Nat Prod*, **52**, 660-662.
739. Li, X.L., Li, Y., Wang, S.F. *et al.* (2009) Ingol and ingenol diterpenes from the aerial parts of Euphorbia royleana and their antiangiogenic activities. *J Nat Prod*, **72**, 1001-1005.
740. Li, X.N., Pu, J.X., Du, X. *et al.* (2010) Structure and cytotoxicity of diterpenoids from Isodon eriocalyx. *J Nat Prod*, **73**, 1803-1809.
741. Li, X.W., Weng, L., Gao, X. *et al.* (2011) Antiproliferative and apoptotic sesquiterpene lactones from Carpesium faberi. *Bioorg Med Chem Lett*, **21**, 366-372.
742. Li, Y., Chen, X., Satake, M. *et al.* (2004) Acetylated flavonoid glycosides potentiating NGF action from Scoparia dulcis. *J Nat Prod*, **67**, 725-727.
743. Li, Y., Cheng, W., Zhu, C. *et al.* (2011) Bioactive neolignans and lignans from the bark of Machilus robusta. *J Nat Prod*, **74**, 1444-1452.
744. Li, Y., Ishibashi, M., Satake, M. *et al.* (2003) Sterol and triterpenoid constituents of Verbena littoralis with NGF-potentiating activity. *J Nat Prod*, **66**, 696-698.
745. Li, Y., Leung, K.T., Yao, F. *et al.* (2006) Antiviral flavans from the leaves of Pithecellobium clypearia. *J Nat Prod*, **69**, 833-835.
746. Li, Y. and Yang, X.W. (2008) [Studies on chemical constituents of root tuber of cultivated Pseudostellaria heterophylla (Zheshen No. 1)]. *Zhongguo Zhong Yao Za Zhi*, **33**, 2353-2355.
747. Li, Y., Yu, S., Liu, D. *et al.* (2012) Inhibitory effects of polyphenols toward HCV from the mangrove plant Excoecaria agallocha L. *Bioorg Med Chem Lett*, **22**, 1099-1102.
748. Li, Y., Zhang, D.M., Li, J.B. *et al.* (2006) Hepatoprotective sesquiterpene glycosides from Sarcandra glabra. *J Nat Prod*, **69**, 616-620.
749. Li, Y.S., Matsunaga, K., Kato, R. *et al.* (2001) Verbenachalcone, a novel dimeric dihydrochalcone with potentiating activity on nerve growth factor-action from Verbena littoralis. *J Nat Prod*, **64**, 806-808.

750. Liang, C., Ding, Y., Nguyen, H.T. *et al.* (2010) Oleanane-type triterpenoids from Panax stipuleanatus and their anticancer activities. *Bioorg Med Chem Lett*, **20**, 7110-7115.
751. Liang, C.Q., Shi, Y.M., Wang, W.G. *et al.* (2015) Kadcoccinic Acids A-J, Triterpene Acids from Kadsura coccinea. *J Nat Prod*, **78**, 2067-2073.
752. Liang, D., Hao, Z.Y., Zhang, G.J. *et al.* (2011) Cytotoxic triterpenoid saponins from Lysimachia clethroides. *J Nat Prod*, **74**, 2128-2136.
753. Liang, S., Shen, Y.H., Feng, Y. *et al.* (2010) Terpenoids from Daphne aurantiaca and their potential anti-inflammatory activity. *J Nat Prod*, **73**, 532-535.
754. Liang, S., Shen, Y.H., Tian, J.M. *et al.* (2008) Phenylpropanoids from Daphne feddei and their inhibitory activities against NO production. *J Nat Prod*, **71**, 1902-1905.
755. Liang, S., Xu, X.W., Zhao, X.F. *et al.* (2016) Two new fatty acids esters were detected in ginseng stems by the application of azoxystrobin and the increasing of antioxidant enzyme activity and ginsenosides content. *Pestic Biochem Physiol*, **134**, 63-72.
756. Liang, S., Ying, S.S., Wu, H.H. *et al.* (2015) A novel sesquiterpene and three new phenolic compounds from the rhizomes of Acorus tatarinowii Schott. *Bioorg Med Chem Lett*, **25**, 4214-4218.
757. Liao, L.M., Vieira, P.C., Rodrigues-Filho, E. *et al.* (2001) Sesquiterpene pyridine alkaloids from Peritassa campestris. *Phytochemistry*, **58**, 1205-1207.
758. Liao, Y.H., Houghton, P.J. and Hoult, J.R. (1999) Novel and known constituents from Buddleja species and their activity against leukocyte eicosanoid generation. *J Nat Prod*, **62**, 1241-1245.
759. Liaw, C.C., Yang, Y.L., Chen, M. *et al.* (2008) Mono-tetrahydrofuran annonaceous acetogenins from Annona squamosa as cytotoxic agents and calcium ion chelators. *J Nat Prod*, **71**, 764-771.
760. Likhitwitayawuid, K., Angerhofer, C.K., Chai, H. *et al.* (1993) Cytotoxic and antimalarial alkaloids from the tubers of Stephania pierrei. *J Nat Prod*, **56**, 1468-1478.
761. Likhitwitayawuid, K., Angerhofer, C.K., Cordell, G.A. *et al.* (1993) Cytotoxic and antimalarial bisbenzylisoquinoline alkaloids from Stephania erecta. *J Nat Prod*, **56**, 30-38.
762. Likhitwitayawuid, K., Kaewamatawong, R., Ruangrungsi, N. *et al.* (1998) Antimalarial naphthoquinones from Nepenthes thorelii. *Planta Med*, **64**, 237-241.
763. Liktor-Busa, E., Simon, A., Toth, G. *et al.* (2007) Ecdysteroids from Serratula wolffii roots. *J Nat Prod*, **70**, 884-886.
764. Lim, H.J., Jin, H.G., Woo, E.R. *et al.* (2013) The root barks of Morus alba and the flavonoid constituents inhibit airway inflammation. *J Ethnopharmacol*, **149**, 169-175.
765. Lim, H.J., Lee, J.H., Choi, J.S. *et al.* (2014) Inhibition of airway inflammation by the roots of Angelica decursiva and its constituent, columbianadin. *J Ethnopharmacol*, **155**, 1353-1361.
766. Lin, B.D., Yuan, T., Zhang, C.R. *et al.* (2009) Structurally diverse limonoids from the fruits of Swietenia mahagoni. *J Nat Prod*, **72**, 2084-2090.
767. Lin, C.N., Kuo, S.H., Chung, M.I. *et al.* (1997) A new flavone C-glycoside and antiplatelet and vasorelaxing flavones from Gentiana arisanensis. *J Nat Prod*, **60**, 851-853.
768. Lin, C.N., Lu, C.M., Lin, H.C. *et al.* (1996) Novel antiplatelet constituents from formosan moraceous plants. *J Nat Prod*, **59**, 834-838.
769. Lin, H.C., Ding, H.Y. and Chang, W.L. (2001) Two new fatty diterpenoids from Salvia miltiorrhiza. *J Nat Prod*, **64**, 648-650.
770. Lin, H.C. and Lee, S.S. (2012) Dibenzocycloheptanoids from the leaves of Cinnamomum subavenium. *J Nat Prod*, **75**, 1735-1743.
771. Lin, L.C., Chiou, C.T. and Cheng, J.J. (2011) 5-deoxyflavones with cytotoxic activity from Mimosa diplotricha. *J Nat Prod*, **74**, 2001-2004.
772. Lin, L.C., Chou, C.J. and Kuo, Y.C. (2001) Cytotoxic principles from Ventilago leiocarpa. *J Nat Prod*, **64**, 674-676.
773. Lin, L.C., Kuo, Y.C. and Chou, C.J. (1999) Immunomodulatory principles of Dichrocephala bicolor. *J Nat Prod*, **62**, 405-408.
774. Lin, L.C., Shen, C.C., Shen, Y.C. *et al.* (2006) Anti-inflammatory neolignans from Piper kadsura. *J Nat Prod*, **69**, 842-844.
775. Lin, L.G., Li, K.M., Tang, C.P. *et al.* (2008) Antitussive stemoninine alkaloids from the roots of Stemona tuberosa. *J Nat Prod*, **71**, 1107-1110.
776. Lin, L.Z., Hu, S.F., Zaw, K. *et al.* (1994) Thalifaberidine, a cytotoxic aporphine-benzylisoquinoline alkaloid from Thalictrum faberi. *J Nat Prod*, **57**, 1430-1436.
777. Lin, L.Z., Shieh, H.L., Angerhofer, C.K. *et al.* (1993) Cytotoxic and antimalarial bisbenzylisoquinoline alkaloids from Cyclea barbata. *J Nat Prod*, **56**, 22-29.
778. Lin, P., Li, S., Wang, S. *et al.* (2006) A nitrogen-containing 3-alkyl-1,4-benzoquinone and a gomphilactone derivative from Embelia ribes. *J Nat Prod*, **69**, 1629-1632.

779. Lin, R.J., Cheng, M.J., Huang, J.C. *et al.* (2009) Cytotoxic compounds from the stems of Cinnamomum tenuifolium. *J Nat Prod*, **72**, 1816-1824.
780. Lin, S., Chen, T., Liu, X.H. *et al.* (2010) Iridoids and lignans from Valeriana jatamansi. *J Nat Prod*, **73**, 632-638.
781. Lin, S., Li, Y.C., Sakurai, N. *et al.* (2001) [Study of sesquiterpene alkaloids from Tripterygium wilfordii Hook.f]. *Yao Xue Xue Bao*, **36**, 116-119.
782. Lin, S., Shen, Y.H., Li, H.L. *et al.* (2009) Acylated iridoids with cytotoxicity from Valeriana jatamansi. *J Nat Prod*, **72**, 650-655.
783. Lin, S., Shen, Y.H., Zhang, Z.X. *et al.* (2010) Revision of the Structures of 1,5-Dihydroxy-3,8-epoxyvalechlorine, Volvaltrate B, and Valeriotetrate C from Valeriana jatamansi and V. officinalis. *J Nat Prod*, **73**, 1723-1726.
784. Lin, T.T., Huang, Y.Y., Tang, G.H. *et al.* (2014) Prenylated coumarins: natural phosphodiesterase-4 inhibitors from Toddalia asiatica. *J Nat Prod*, **77**, 955-962.
785. Lin, W., Wang, Y., Lin, S. *et al.* (2012) Induction of cell cycle arrest by the carbazole alkaloid Clauszoline-I from Clausena vestita D. D. Tao via inhibition of the PKCdelta phosphorylation. *Eur J Med Chem*, **47**, 214-220.
786. Lin, Y.L., Shen, C.C., Huang, Y.J. *et al.* (2005) Homoflavonoids from Ophioglossum petiolatum. *J Nat Prod*, **68**, 381-384.
787. Lin, Y.M., Anderson, H., Flavin, M.T. *et al.* (1997) In vitro anti-HIV activity of biflavonoids isolated from Rhus succedanea and Garcinia multiflora. *J Nat Prod*, **60**, 884-888.
788. Lin, Z., Guo, Y., Gao, Y. *et al.* (2015) ent-Kaurane Diterpenoids from Chinese Liverworts and Their Antitumor Activities through Michael Addition As Detected in Situ by a Fluorescence Probe. *J Med Chem*, **58**, 3944-3956.
789. Lin, Z., Hoult, J.R., Bennett, D.C. *et al.* (1999) Stimulation of mouse melanocyte proliferation by Piper nigrum fruit extract and its main alkaloid, piperine. *Planta Med*, **65**, 600-603.
790. Linn, T.Z., Awale, S., Tezuka, Y. *et al.* (2005) Cassane- and norcassane-type diterpenes from Caesalpinia crista of Indonesia and their antimalarial activity against the growth of Plasmodium falciparum. *J Nat Prod*, **68**, 706-710.
791. Liobikas, J., Majiene, D., Trumbeckaite, S. *et al.* (2011) Uncoupling and antioxidant effects of ursolic acid in isolated rat heart mitochondria. *J Nat Prod*, **74**, 1640-1644.
792. Liu, A.H., Liu, D.Q., Liang, T.J. *et al.* (2013) Caulerprenylols A and B, two rare antifungal prenylated para-xylenes from the green alga Caulerpa racemosa. *Bioorg Med Chem Lett*, **23**, 2491-2494.
793. Liu, B., Kongstad, K.T., Qinglei, S. *et al.* (2015) Dual high-resolution alpha-glucosidase and radical scavenging profiling combined with HPLC-HRMS-SPE-NMR for identification of minor and major constituents directly from the crude extract of Pueraria lobata. *J Nat Prod*, **78**, 294-300.
794. Liu, B., Zhang, T., Zhang, X. *et al.* (2010) [Chemical constituents of Laggera pterodonta]. *Zhongguo Zhong Yao Za Zhi*, **35**, 602-606.
795. Liu, H., Chao, Z., Wu, X. *et al.* (2012) [Chemical constituents contained in Populus tomentosa]. *Zhongguo Zhong Yao Za Zhi*, **37**, 1422-1425.
796. Liu, H., Li, C.J., Yang, J.Z. *et al.* (2012) Carbazole alkaloids from the stems of Clausena lansium. *J Nat Prod*, **75**, 677-682.
797. Liu, J., Dumontet, V., Simonin, A.L. *et al.* (2011) Benzofurans from Styrax agrestis as acetylcholinesterase inhibitors: structure-activity relationships and molecular modeling studies. *J Nat Prod*, **74**, 2081-2088.
798. Liu, J., Li, Q., Yin, Y. *et al.* (2014) Ultra-fast LC-ESI-MS/MS method for the simultaneous determination of six highly toxic Aconitum alkaloids from Aconiti kusnezoffii radix in rat plasma and its application to a pharmacokinetic study. *J Sep Sci*, **37**, 171-178.
799. Liu, L.G. and Tan, R.X. (2001) New jatrophane diterpenoid esters from Euphorbia turczaninowii. *J Nat Prod*, **64**, 1064-1068.
800. Liu, L.P., Han, K., Chen, W. *et al.* (2014) Topoisomerase II inhibitors from the roots of Stellera chamaejasme L. *Bioorg Med Chem*, **22**, 4198-4203.
801. Liu, Q., Wang, Y.F., Chen, R.J. *et al.* (2009) Anti-coxsackie virus B3 norsesquiterpenoids from the roots of Phyllanthus emblica. *J Nat Prod*, **72**, 969-972.
802. Liu, Q., Yang, Q.M., Hu, H.J. *et al.* (2014) Bioactive diterpenoids and flavonoids from the aerial parts of Scoparia dulcis. *J Nat Prod*, **77**, 1594-1600.
803. Liu, Q.H., Jeong, J.E., Choi, E.J. *et al.* (2006) A new furofuran lignan from Geranium thunbergii Sieb. et Zucc. *Arch Pharm Res*, **29**, 1109-1113.
804. Liu, S., Zhu, H., Zhang, S. *et al.* (2008) Abietane diterpenoids from Clerodendrum bungei. *J Nat Prod*, **71**, 755-759.

805. Liu, X., Yang, J., Wang, W.G. *et al.* (2015) Diterpene alkaloids with an aza-ent-kaurane skeleton from Isodon rubescens. *J Nat Prod*, **78**, 196-201.
806. Liu, X., Yu, T., Gao, X.M. *et al.* (2010) Apoptotic effects of polyprenylated benzoylphloroglucinol derivatives from the twigs of Garcinia multiflora. *J Nat Prod*, **73**, 1355-1359.
807. Liu, X.T., Pan, Q., Shi, Y. *et al.* (2006) ent-rosane and labdane diterpenoids from Sagittaria sagittifolia and their antibacterial activity against three oral pathogens. *J Nat Prod*, **69**, 255-260.
808. Liu, X.X., Alali, F.Q., Pilarinou, E. *et al.* (1998) Glacins A and B: two novel bioactive mono-tetrahydrofuran acetogenins from Annona glabra. *J Nat Prod*, **61**, 620-624.
809. Liu, X.X., Pilarinou, E. and McLaughlin, J.L. (1999) Two novel acetogenins, annoglaxin and 27-hydroxybullatacin, from Annona glabra. *J Nat Prod*, **62**, 848-852.
810. Liu, Y., Harinantenaina, L., Brodie, P.J. *et al.* (2013) Bioactive compounds from Stuhlmannia moavi from the Madagascar dry forest. *Bioorg Med Chem*, **21**, 7591-7594.
811. Liu, Y., Ma, J., Zhao, Q. *et al.* (2013) Guaiane-type sesquiterpenes from Curcuma phaeocaulis and their inhibitory effects on nitric oxide production. *J Nat Prod*, **76**, 1150-1156.
812. Liu, Y.F., Liang, D., Luo, H. *et al.* (2012) Hepatoprotective iridoid glycosides from the roots of Rehmannia glutinosa. *J Nat Prod*, **75**, 1625-1631.
813. Liu, Y.L., Ho, D.K., Cassady, J.M. *et al.* (1992) Isolation of potential cancer chemopreventive agents from Eriodictyon californicum. *J Nat Prod*, **55**, 357-363.
814. Liu, Y.P., Cai, X.H., Feng, T. *et al.* (2011) Triterpene and sterol derivatives from the roots of Breynia fruticosa. *J Nat Prod*, **74**, 1161-1168.
815. Liu, Z., Zhao, R. and Zou, Z. (2011) [Chemical constituents from root bark of Tripterygium hypoglaucum]. *Zhongguo Zhong Yao Za Zhi*, **36**, 2503-2506.
816. Liu, Z.G., Li, Z.L., Bai, J. *et al.* (2014) Anti-inflammatory diterpenoids from the roots of Euphorbia ebracteolata. *J Nat Prod*, **77**, 792-799.
817. Liu, Z.L., Cao, J., Zhang, H.M. *et al.* (2011) Feeding deterrents from Aconitum episcopale roots against the red flour beetle, Tribolium castaneum. *J Agric Food Chem*, **59**, 3701-3706.
818. Long, C., Marcourt, L., Raux, R. *et al.* (2009) Meroterpenes from Dichrostachys cinerea inhibit protein farnesyl transferase activity. *J Nat Prod*, **72**, 1804-1815.
819. Lopez, J.A., Lin, F.T., Duah, F.K. *et al.* (1988) Mollinedine, a New Alkaloid from Mollinedia costaricensis. *J Nat Prod*, **51**, 754-759.
820. Loukaci, A., Kayser, O., Bindseil, K. *et al.* (2000) New trichothecenes isolated from Holarrhena floribunda. *J Nat Prod*, **63**, 52-56.
821. Lu, C.H., Li, Y.Y., Li, L.J. *et al.* (2012) Anti-inflammatory activities of fractions from Geranium nepalense and related polyphenols. *Drug Discov Ther*, **6**, 194-197.
822. Lu, W., Wang, X., Chen, J. *et al.* (1998) [Studies on the chemical constituents of chloroform extract of Dracaena cochinchinensis]. *Yao Xue Xue Bao*, **33**, 755-758.
823. Lu, Y., Xue, Y., Liu, J. *et al.* (2015) (+/-)-Acortatarinowins A-F, Norlignan, Neolignan, and Lignan Enantiomers from Acorus tatarinowii. *J Nat Prod*, **78**, 2205-2214.
824. Lu, Z., Sun, W., Duan, X. *et al.* (2012) [Chemical constituents from Corydalis yanhusuo]. *Zhongguo Zhong Yao Za Zhi*, **37**, 235-237.
825. Lu, Z.Q., Guan, S.H., Li, X.N. *et al.* (2008) Cytotoxic diterpenoids from Euphorbia helioscopia. *J Nat Prod*, **71**, 873-876.
826. Ludwig, R.A. (1993) Arabidopsis chloroplasts dissimilate L-arginine and L-citrulline for use as N source. *Plant Physiol*, **101**, 429-434.
827. Luecha, P., Umehara, K., Miyase, T. *et al.* (2009) Antiestrogenic constituents of the Thai medicinal plants Capparis flavicans and Vitex glabrata. *J Nat Prod*, **72**, 1954-1959.
828. Luo, S.Y., Chen, J.F., Zhong, Z.G. *et al.* (2016) Salvianolic acid B stimulates osteogenesis in dexamethasone-treated zebrafish larvae. *Acta Pharmacol Sin*, **37**, 1370-1380.
829. Luo, Y., Pu, X., Luo, G. *et al.* (2014) Nitrogen-containing dihydro-beta-agarofuran derivatives from Tripterygium wilfordii. *J Nat Prod*, **77**, 1650-1657.
830. Luo, Y., Zhou, M., Ye, Q. *et al.* (2012) Dihydroagarofuran derivatives from the dried roots of Tripterygium wilfordii. *J Nat Prod*, **75**, 98-102.
831. Luyen, B.T., Tai, B.H., Thao, N.P. *et al.* (2014) A new phenylpropanoid and an alkylglycoside from Piper retrofractum leaves with their antioxidant and alpha-glucosidase inhibitory activity. *Bioorg Med Chem Lett*, **24**, 4120-4124.
832. Lv, H.N., Wang, S., Zeng, K.W. *et al.* (2015) Anti-inflammatory coumarin and benzocoumarin derivatives from Murraya alata. *J Nat Prod*, **78**, 279-285.
833. Lv, J.J., Xu, M., Wang, D. *et al.* (2013) Cytotoxic bisbenzylisoquinoline alkaloids from Stephania epigaea. *J Nat Prod*, **76**, 926-932.

834. Ma, C., Nakamura, N., Hattori, M. *et al.* (2000) Inhibitory effects on HIV-1 protease of constituents from the wood of Xanthoceras sorbifolia. *J Nat Prod*, **63**, 238-242.
835. Ma, C., Wang, W., Chen, Y.Y. *et al.* (2005) Neuroprotective and antioxidant activity of compounds from the aerial parts of Dioscorea opposita. *J Nat Prod*, **68**, 1259-1261.
836. Ma, C.J., Kim, Y.C. and Sung, S.H. (2009) Compounds with neuroprotective activity from the medicinal plant Machilus thunbergii. *J Enzyme Inhib Med Chem*, **24**, 1117-1121.
837. Ma, C.Y., Liu, W.K. and Che, C.T. (2002) Lignanamides and nonalkaloidal components of Hyoscyamus niger seeds. *J Nat Prod*, **65**, 206-209.
838. Ma, G., Wu, H., Chen, D. *et al.* (2015) Antimalarial and Antiproliferative Cassane Diterpenes of Caesalpinia sappan. *J Nat Prod*, **78**, 2364-2371.
839. Ma, L., Gu, R., Tang, L. *et al.* (2015) Important poisonous plants in tibetan ethnomedicine. *Toxins (Basel)*, **7**, 138-155.
840. Ma, L., Yang, X.W., Xu, W. *et al.* (2009) Intestinal permeability of antitumor alkaloids from the processed seeds of Strychnos nux-vomica in a Caco-2 cell model. *Planta Med*, **75**, 631-634.
841. Ma, L.Y., Zhou, Q.L. and Yang, X.W. (2015) New SIRT1 activator from alkaline hydrolysate of total saponins in the stems-leaves of Panax ginseng. *Bioorg Med Chem Lett*, **25**, 5321-5325.
842. Ma, Y. and Han, G. (1995) [Biologically active lignins from Magnolia biondii Pamp]. *Zhongguo Zhong Yao Za Zhi*, **20**, 102-104, 127.
843. Ma, Y., Han, G.Q. and Liu, Z.J. (1993) [Studies on PAF antagonistic bicyclo(3,2,1) octanoid neolignans from Piper kadsura]. *Yao Xue Xue Bao*, **28**, 207-211.
844. Ma, Y., Han, G.Q. and Wang, Y.Y. (1993) [PAF antagonistic benzofuran neolignans from Piper kadsura]. *Yao Xue Xue Bao*, **28**, 370-373.
845. MacKinnon, S., Durst, T., Arnason, J.T. *et al.* (1997) Antimalarial activity of tropical Meliaceae extracts and gedunin derivatives. *J Nat Prod*, **60**, 336-341.
846. MacLeod, J.K., Moeller, P.D. and Franke, F.P. (1990) Two toxic kaurene glycosides from the burrs of Xanthium pungens. *J Nat Prod*, **53**, 451-455.
847. Maggi, F., Quassinti, L., Bramucci, M. *et al.* (2014) Composition and biological activities of hogweed [Heracleum sphondylium L. subsp. ternatum (Velen.) Brummitt] essential oil and its main components octyl acetate and octyl butyrate. *Nat Prod Res*, **28**, 1354-1363.
848. Mahabusarakam, W., Nuangnaowarat, W. and Taylor, W.C. (2006) Xanthone derivatives from Cratoxylum cochinchinense roots. *Phytochemistry*, **67**, 470-474.
849. Mai, L.H., Chabot, G.G., Grellier, P. *et al.* (2015) Antivascular and anti-parasite activities of natural and hemisynthetic flavonoids from New Caledonian Gardenia species (Rubiaceae). *Eur J Med Chem*, **93**, 93-100.
850. Maldonado, E., Diaz-Arumir, H., Toscano, R.A. *et al.* (2010) Lupane triterpenes with a delta-lactone at ring E, from Lippia mexicana. *J Nat Prod*, **73**, 1969-1972.
851. Manayi, A., Saeidnia, S., Ostad, S.N. *et al.* (2013) Chemical constituents and cytotoxic effect of the main compounds of Lythrum salicaria L. *Z Naturforsch C*, **68**, 367-375.
852. Mancini, S.D. and Edwards, J.M. (1979) Cytotoxic principles from the sap of Kalmia latifolia. *J Nat Prod*, **42**, 483-488.
853. Manfredi, K.P., Vallurupalli, V., Demidova, M. *et al.* (2001) Isolation of an anti-HIV diprenylated bibenzyl from Glycyrrhiza lepidota. *Phytochemistry*, **58**, 153-157.
854. Manir, M.M., Kim, J.K., Lee, B.G. *et al.* (2012) Tea catechins and flavonoids from the leaves of Camellia sinensis inhibit yeast alcohol dehydrogenase. *Bioorg Med Chem*, **20**, 2376-2381.
855. Marin, C., Ramirez-Macias, I., Lopez-Cespedes, A. *et al.* (2011) In vitro and in vivo trypanocidal activity of flavonoids from Delphinium staphisagria against Chagas disease. *J Nat Prod*, **74**, 744-750.
856. Marrassini, C., Davicino, R., Acevedo, C. *et al.* (2011) Vicenin-2, a potential anti-inflammatory constituent of Urtica circularis. *J Nat Prod*, **74**, 1503-1507.
857. Martin, F., Hay, A.E., Quinteros Condoretty, V.R. *et al.* (2009) Antioxidant phenylethanoid glycosides and a neolignan from Jacaranda caucana. *J Nat Prod*, **72**, 852-856.
858. Martin, O.R., Compain, P., Kizu, H. *et al.* (1999) Revised structure of a homonojirimycin isomer from Aglaonema treubii: first example of a naturally occurring alpha-homoallonojirimycin. *Bioorg Med Chem Lett*, **9**, 3171-3174.
859. Mativandlela, S.P., Muthivhi, T., Kikuchi, H. *et al.* (2009) Antimycobacterial flavonoids from the leaf extract of Galenia africana. *J Nat Prod*, **72**, 2169-2171.
860. Matochko, W.L., James, A., Lam, C.W. *et al.* (2010) Triterpenoidal alkaloids from Buxus natalensis and their acetylcholinesterase inhibitory activity. *J Nat Prod*, **73**, 1858-1862.
861. Matsuda, H., Kageura, T., Morikawa, T. *et al.* (2000) Effects of stilbene constituents from rhubarb on nitric oxide production in lipopolysaccharide-activated macrophages. *Bioorg Med Chem Lett*, **10**, 323-327.

862. Matsuda, H., Kawaguchi, Y., Yamazaki, M. *et al.* (2004) Melanogenesis stimulation in murine B16 melanoma cells by Piper nigrum leaf extract and its lignan constituents. *Biol Pharm Bull*, **27**, 1611-1616.
863. Matsuda, H., Morikawa, T., Managi, H. *et al.* (2003) Antiallergic principles from Alpinia galanga: structural requirements of phenylpropanoids for inhibition of degranulation and release of TNF-alpha and IL-4 in RBL-2H3 cells. *Bioorg Med Chem Lett*, **13**, 3197-3202.
864. Matsuda, H., Murakami, T., Kageura, T. *et al.* (1998) Hepatoprotective and nitric oxide production inhibitory activities of coumarin and polyacetylene constituents from the roots of Angelica furcijuga. *Bioorg Med Chem Lett*, **8**, 2191-2196.
865. Matsuda, H., Nakashima, S., Oda, Y. *et al.* (2009) Melanogenesis inhibitors from the rhizomes of Alpinia officinarum in B16 melanoma cells. *Bioorg Med Chem*, **17**, 6048-6053.
866. Matsuda, H., Shimoda, H., Morikawa, T. *et al.* (2001) Phytoestrogens from the roots of Polygonum cuspidatum (Polygonaceae): structure-requirement of hydroxyanthraquinones for estrogenic activity. *Bioorg Med Chem Lett*, **11**, 1839-1842.
867. Matsuda, H., Shimoda, H., Ninomiya, K. *et al.* (2002) Inhibitory mechanism of costunolide, a sesquiterpene lactone isolated from Laurus nobilis, on blood-ethanol elevation in rats: involvement of inhibition of gastric emptying and increase in gastric juice secretion. *Alcohol Alcohol*, **37**, 121-127.
868. Matsuda, H., Yoshida, K., Miyagawa, K. *et al.* (2007) Rotenoids and flavonoids with anti-invasion of HT1080, anti-proliferation of U937, and differentiation-inducing activity in HL-60 from Erycibe expansa. *Bioorg Med Chem*, **15**, 1539-1546.
869. Matsunaga, H., Katano, M., Yamamoto, H. *et al.* (1990) Cytotoxic activity of polyacetylene compounds in Panax ginseng C. A. Meyer. *Chem Pharm Bull (Tokyo)*, **38**, 3480-3482.
870. Matsunaga, K., Ikeda, M., Shibuya, M. *et al.* (1994) Cylindol A, a novel biphenyl ether with 5-lipoxygenase inhibitory activity, and a related compound from Imperata Cylindrica. *J Nat Prod*, **57**, 1290-1293.
871. Matsunaga, K., Shibuya, M. and Ohizumi, Y. (1994) Graminone B, a novel lignan with vasodilative activity from Imperata cylindrica. *J Nat Prod*, **57**, 1734-1736.
872. Matsuno, Y., Deguchi, J., Hirasawa, Y. *et al.* (2008) Sucutiniranes A and B, new cassane-type diterpenes from Bowdichia nitida. *Bioorg Med Chem Lett*, **18**, 3774-3777.
873. Matsuno, Y., Deguchi, J., Hosoya, T. *et al.* (2009) Sucutiniranes C-F, cassane-type diterpenes from Bowdichia nitida. *J Nat Prod*, **72**, 976-979.
874. Matsuura, S. (1957) The structure of cryptostrobin and strobopinin; the flavanones from the heartwood of Pinus strobus. *Pharm Bull*, **5**, 195-198.
875. Maximo, P., Lourenco, A., Feio, S.S. *et al.* (2002) Flavonoids from Ulex airensis and Ulex europaeus ssp. europaeus. *J Nat Prod*, **65**, 175-178.
876. Mbwambo, Z.H., Kapingu, M.C., Moshi, M.J. *et al.* (2006) Antiparasitic activity of some xanthones and biflavonoids from the root bark of Garcinia livingstonei. *J Nat Prod*, **69**, 369-372.
877. McKee, T.C., Fuller, R.W., Covington, C.D. *et al.* (1996) New pyranocoumarins isolated from Calophyllum lanigerum and Calophyllum teysmannii. *J Nat Prod*, **59**, 754-758.
878. McLaughlin, J.L., Miller, R.W., Powell, R.G. *et al.* (1981) 19-Hydroxybaccatin III, 10-deacetylcephalomannine, and 10-deacetyltaxol: new antitumor taxanes from Taxus wallichiana. *J Nat Prod*, **44**, 312-319.
879. Mei, R.Q., Wang, Y.H., Du, G.H. *et al.* (2009) Antioxidant Lignans from the Fruits of Broussonetia papyrifera. *J Nat Prod*, **72**, 621-625.
880. Mena-Rejon, G.J., Perez-Espadas, A.R., Moo-Puc, R.E. *et al.* (2007) Antigiardial activity of triterpenoids from root bark of Hippocratea excelsa. *J Nat Prod*, **70**, 863-865.
881. Mencherini, T., Picerno, P., Festa, M. *et al.* (2011) Triterpenoid constituents from the roots of Paeonia rockii ssp. rockii. *J Nat Prod*, **74**, 2116-2121.
882. Mencherini, T., Picerno, P., Russo, P. *et al.* (2009) Composition of the fresh leaves and stems of Melissa officinalis and evaluation of skin irritation in a reconstituted human epidermis model. *J Nat Prod*, **72**, 1512-1515.
883. Mencherini, T., Picerno, P., Scesa, C. *et al.* (2007) Triterpene, antioxidant, and antimicrobial compounds from Melissa officinalis. *J Nat Prod*, **70**, 1889-1894.
884. Mendoza-Espinoza, J.A., Lopez-Vallejo, F., Fragoso-Serrano, M. *et al.* (2009) Structural reassignment, absolute configuration, and conformation of hypurticin, a highly flexible polyacyloxy-6-heptenyl-5,6-dihydro-2H-pyran-2-one. *J Nat Prod*, **72**, 700-708.
885. Menkovic, N., Savikin-Fodulovic, K., Bulatovic, V. *et al.* (2002) Xanthones from Swertia punctata. *Phytochemistry*, **61**, 415-420.
886. Menkovic, N., Savikin-Fodulovic, K., Momcilovic, I. *et al.* (2000) Quantitative determination of secoiridoid and gamma-pyrone compounds in Gentiana lutea cultured in vitro. *Planta Med*, **66**, 96-98.

887. Meragelman, K.M., McKee, T.C. and Boyd, M.R. (2000) Siamenol, a new carbazole alkaloid from Murraya siamensis. *J Nat Prod*, **63**, 427-428.
888. Mesa-Siverio, D., Machin, R.P., Estevez-Braun, A. *et al.* (2008) Structure and estrogenic activity of new lignans from Iryanthera lancifolia. *Bioorg Med Chem*, **16**, 3387-3394.
889. Metzler-Zebeli, B.U., Deckardt, K., Schollenberger, M. *et al.* (2014) Lactic acid and thermal treatments trigger the hydrolysis of myo-inositol hexakisphosphate and modify the abundance of lower myo-inositol phosphates in barley (Hordeum vulgare L.). *PLoS One*, **9**, e101166.
890. Meyer, A. and Imming, P. (2011) Benzylisoquinoline alkaloids from the papaveraceae: the heritage of Johannes Gadamer (1867-1928). *J Nat Prod*, **74**, 2482-2487.
891. Miliauskas, G., van Beek, T.A., de Waard, P. *et al.* (2005) Identification of radical scavenging compounds in Rhaponticum carthamoides by means of LC-DAD-SPE-NMR. *J Nat Prod*, **68**, 168-172.
892. Mimaki, Y., Kuroda, M., Asano, T. *et al.* (1999) Triterpene saponins and lignans from the roots of Pulsatilla chinensis and their cytotoxic activity against HL-60 cells. *J Nat Prod*, **62**, 1279-1283.
893. Mimaki, Y., Yokosuka, A., Hamanaka, M. *et al.* (2004) Triterpene saponins from the roots of Clematis chinensis. *J Nat Prod*, **67**, 1511-1516.
894. Mimaki, Y., Yokosuka, A., Kuroda, M. *et al.* (2001) New bisdesmosidic triterpene saponins from the roots of Pulsatilla chinensis. *J Nat Prod*, **64**, 1226-1229.
895. Min, B.S., Cuong, T.D., Hung, T.M. *et al.* (2012) Compounds from the heartwood of Caesalpinia sappan and their anti-inflammatory activity. *Bioorg Med Chem Lett*, **22**, 7436-7439.
896. Minakawa, T., Toume, K., Arai, M.A. *et al.* (2012) Eudesmane-type sesquiterpenoid and guaianolides from Kandelia candel in a screening program for compounds to overcome TRAIL resistance. *J Nat Prod*, **75**, 1431-1435.
897. Misico, R.I., Song, L.L., Veleiro, A.S. *et al.* (2002) Induction of quinone reductase by withanolides. *J Nat Prod*, **65**, 677-680.
898. Mitscher, L.A., Park, Y.H., Clark, D. *et al.* (1980) Antimicrobial agents from higher plants. Antimicrobial isoflavanoids and related substances from Glycyrrhiza glabra L. var. typica. *J Nat Prod*, **43**, 259-269.
899. Miyazawa, M., Okuno, Y., Fukuyama, M. *et al.* (1999) Antimutagenic activity of polymethoxyflavonoids from Citrus aurantium. *J Agric Food Chem*, **47**, 5239-5244.
900. Mohamad, H., Lajis, N.H., Abas, F. *et al.* (2005) Antioxidative constituents of Etlingera elatior. *J Nat Prod*, **68**, 285-288.
901. Mohamad, K., Hirasawa, Y., Litaudon, M. *et al.* (2009) Ceramicines B-D, new antiplasmodial limonoids from Chisocheton ceramicus. *Bioorg Med Chem*, **17**, 727-730.
902. Molina, P., Tarraga, A., Gonzalez-Tejero, A. *et al.* (2001) Inhibition of leukocyte functions by the alkaloid isaindigotone from Isatis indigotica and some new synthetic derivatives. *J Nat Prod*, **64**, 1297-1300.
903. Molyneux, R.J., Pan, Y.T., Tropea, J.E. *et al.* (1993) 2-Hydroxymethyl-3,4-dihydroxy-6-methyl-pyrrolidine (6-deoxy-DMDP), an alkaloid beta-mannosidase inhibitor from seeds of Angylocalyx pynaertii. *J Nat Prod*, **56**, 1356-1364.
904. Montilla, M.P., Agil, A., Navarro, M.C. *et al.* (2003) Antioxidant activity of maslinic acid, a triterpene derivative obtained from Olea europaea. *Planta Med*, **69**, 472-474.
905. Moon, H.I., Chung, J.H., Lee, J.K. *et al.* (2004) Triterpenoid saponin from Viola hondoensis W. Becker et H Boss. and their effect on MMP-1 and type I procollagen expression. *Arch Pharm Res*, **27**, 730-733.
906. Moon, S.S., Rahman, A.A., Kim, J.Y. *et al.* (2008) Hanultarin, a cytotoxic lignan as an inhibitor of actin cytoskeleton polymerization from the seeds of Trichosanthes kirilowii. *Bioorg Med Chem*, **16**, 7264-7269.
907. Morad, S.A., Schmidt, C., Buchele, B. *et al.* (2011) (8R)-3beta,8-dihydroxypolypoda-13E,17E,21-triene induces cell cycle arrest and apoptosis in treatment-resistant prostate cancer cells. *J Nat Prod*, **74**, 1731-1736.
908. Morgan, A.M., Lee, H.W., Lee, S.H. *et al.* (2014) Anti-osteoporotic and antioxidant activities of chemical constituents of the aerial parts of Ducrosia ismaelis. *Bioorg Med Chem Lett*, **24**, 3434-3439.
909. Mori-Hongo, M., Takimoto, H., Katagiri, T. *et al.* (2009) Melanin synthesis inhibitors from Lespedeza floribunda. *J Nat Prod*, **72**, 194-203.
910. Morikawa, T., Ando, S., Matsuda, H. *et al.* (2005) Inhibitors of nitric oxide production from the rhizomes of Alpinia galanga: structures of new 8-9' linked neolignans and sesquineolignan. *Chem Pharm Bull (Tokyo)*, **53**, 625-630.
911. Morikawa, T., Kishi, A., Pongpiriyadacha, Y. *et al.* (2003) Structures of new friedelane-type triterpenes and eudesmane-type sesquiterpene and aldose reductase inhibitors from Salacia chinensis. *J Nat Prod*, **66**, 1191-1196.
912. Morikawa, T., Li, N., Nagatomo, A. *et al.* (2006) Triterpene saponins with gastroprotective effects from tea seed (the seeds of Camellia sinensis). *J Nat Prod*, **69**, 185-190.

913. Morikawa, T., Matsuda, H., Toguchida, I. *et al.* (2002) Absolute stereostructures of three new sesquiterpenes from the fruit of Alpinia oxyphylla with inhibitory effects on nitric oxide production and degranulation in RBL-2H3 cells. *J Nat Prod*, **65**, 1468-1474.
914. Morikawa, T., Pan, Y., Ninomiya, K. *et al.* (2010) Acylated phenylethanoid oligoglycosides with hepatoprotective activity from the desert plant Cistanche tubulosa. *Bioorg Med Chem*, **18**, 1882-1890.
915. Morikawa, T., Sueyoshi, M., Chaipech, S. *et al.* (2012) Suppressive effects of coumarins from Mammea siamensis on inducible nitric oxide synthase expression in RAW264.7 cells. *Bioorg Med Chem*, **20**, 4968-4977.
916. Morikawa, T., Tao, J., Ando, S. *et al.* (2003) Absolute stereostructures of new arborinane-type triterpenoids and inhibitors of nitric oxide production from Rubia yunnanensis. *J Nat Prod*, **66**, 638-645.
917. Morikawa, T., Tao, J., Toguchida, I. *et al.* (2003) Structures of new cyclic diarylheptanoids and inhibitors of nitric oxide production from Japanese folk medicine Acer nikoense. *J Nat Prod*, **66**, 86-91.
918. Morikawa, T., Xu, F., Matsuda, H. *et al.* (2006) Structures of new flavonoids, erycibenins D, E, and F, and NO production inhibitors from Erycibe expansa originating in Thailand. *Chem Pharm Bull (Tokyo)*, **54**, 1530-1534.
919. Morimoto, M., Kumeda, S. and Komai, K. (2000) Insect antifeedant flavonoids from Gnaphalium affine D. Don. *J Agric Food Chem*, **48**, 1888-1891.
920. Morita, H., Enomoto, M., Hirasawa, Y. *et al.* (2007) Cyclonatsudamine A, a new vasodilator cyclic peptide from Citrus natsudaidai. *Bioorg Med Chem Lett*, **17**, 5410-5413.
921. Morita, H., Koyama, K., Sugimoto, Y. *et al.* (2005) Antimitotic activity and reversal of breast cancer resistance protein-mediated drug resistance by stilbenoids from Bletilla striata. *Bioorg Med Chem Lett*, **15**, 1051-1054.
922. Morita, H., Machida, I., Hirasawa, Y. *et al.* (2005) Taxezopidines M and N, taxoids from the Japanese yew, Taxus cuspidata. *J Nat Prod*, **68**, 935-937.
923. Morita, H., Oshimi, S., Hirasawa, Y. *et al.* (2007) Cassiarins A and B, novel antiplasmodial alkaloids from Cassia siamea. *Org Lett*, **9**, 3691-3693.
924. Morita, H., Sato, Y., Chan, K.L. *et al.* (2000) Samoquasine A, a benzoquinazoline alkaloid from the seeds of Annona squamosa. *J Nat Prod*, **63**, 1707-1708.
925. Moritoki, H., Hisayama, T., Kida, K. *et al.* (1996) Inhibition by triptoquinone-A of LPS- and IL-1 beta-primed induction of NO synthase in rat thoracic aorta. *Life Sci*, **59**, Pl49-54.
926. Motai, T., Daikonya, A. and Kitanaka, S. (2004) Sesquiterpene coumarins from Ferula fukanensis and nitric oxide production inhibitory effects. *J Nat Prod*, **67**, 432-436.
927. Motai, T. and Kitanaka, S. (2005) Sesquiterpene chromones from Ferula fukanensis and their nitric oxide production inhibitory effects. *J Nat Prod*, **68**, 1732-1735.
928. Motai, T. and Kitanaka, S. (2005) Sesquiterpene phenylpropanoids from Ferula fukanensis and their nitric oxide production inhibitory effects. *J Nat Prod*, **68**, 365-368.
929. Mueller, D., Davis, R.A., Duffy, S. *et al.* (2009) Antimalarial activity of azafluorenone alkaloids from the Australian tree Mitrephora diversifolia. *J Nat Prod*, **72**, 1538-1540.
930. Muhammad, A., Guerrero-Analco, J.A., Martineau, L.C. *et al.* (2012) Antidiabetic compounds from Sarracenia purpurea used traditionally by the Eeyou Istchee Cree First Nation. *J Nat Prod*, **75**, 1284-1288.
931. Muhammad, I., Dunbar, D.C., Khan, S.I. *et al.* (2003) Antiparasitic alkaloids from Psychotria klugii. *J Nat Prod*, **66**, 962-967.
932. Mukhopadhyay, S. and Cordell, G.A. (1981) Catharanthus alkaloids. XXXVI. Isolation of vincaleukoblastine (VLB) and periformyline from Catharanthus trichophyllus and pericyclivine from Catharanthus roseus. *J Nat Prod*, **44**, 335-339.
933. Mukhopadhyay, S., El-Sayed, A., Handy, G.A. *et al.* (1983) Catharanthus alkaloids XXXVII. 16-Epi-Z-isositsirikine, a monomeric indole alkaloid with antineoplastic activity from Catharanthus roseus and Rhazya stricta. *J Nat Prod*, **46**, 409-413.
934. Mukker, J.K., Michel, D., Muir, A.D. *et al.* (2014) Permeability and conjugative metabolism of flaxseed lignans by Caco-2 human intestinal cells. *J Nat Prod*, **77**, 29-34.
935. Munoz, J., Mudge, S.M. and Sandoval, A. (2004) Effects of ionic strength on the production of short chain volatile hydrocarbons by Dunaliella salina (Teodoresco). *Chemosphere*, **54**, 1267-1271.
936. Muraoka, O., Fujimoto, M., Tanabe, G. *et al.* (2001) Absolute stereostructures of novel norcadinane- and trinoreudesmane-type sesquiterpenes with nitric oxide production inhibitory activity from Alpinia oxyphylla. *Bioorg Med Chem Lett*, **11**, 2217-2220.
937. Murphy, B.T., Cao, S., Norris, A. *et al.* (2005) Cytotoxic flavanones of Schizolaena hystrix from the Madagascar rainforest. *J Nat Prod*, **68**, 417-419.
938. Musah, R.A., Lesiak, A.D., Maron, M.J. *et al.* (2016) Mechanosensitivity below Ground: Touch-Sensitive Smell-Producing Roots in the Shy Plant Mimosa pudica. *Plant Physiol*, **170**, 1075-1089.

939. Musthafa, K.S., Sianglum, W., Saising, J. *et al.* (2017) Evaluation of phytochemicals from medicinal plants of Myrtaceae family on virulence factor production by Pseudomonas aeruginosa. *Apmis*, **125**, 482-490.
940. Na, M., Cui, L., Min, B.S. *et al.* (2006) Protein tyrosine phosphatase 1B inhibitory activity of triterpenes isolated from Astilbe koreana. *Bioorg Med Chem Lett*, **16**, 3273-3276.
941. Na, M., Jang, J., Min, B.S. *et al.* (2006) Fatty acid synthase inhibitory activity of acylphloroglucinols isolated from Dryopteris crassirhizoma. *Bioorg Med Chem Lett*, **16**, 4738-4742.
942. Nair, J.J., Rarova, L., Strnad, M. *et al.* (2012) Apoptosis-inducing effects of distichamine and narciprimine, rare alkaloids of the plant family Amaryllidaceae. *Bioorg Med Chem Lett*, **22**, 6195-6199.
943. Najmuldeen, I.A., Hadi, A.H., Awang, K. *et al.* (2011) Chisomicines A-C, limonoids from Chisocheton ceramicus. *J Nat Prod*, **74**, 1313-1317.
944. Nakagawa, H., Takaishi, Y., Fujimoto, Y. *et al.* (2004) Chemical constituents from the Colombian medicinal plant Maytenus laevis. *J Nat Prod*, **67**, 1919-1924.
945. Nakahashi, A., Yaguchi, Y., Miura, N. *et al.* (2011) A vibrational circular dichroism approach to the determination of the absolute configurations of flavorous 5-substituted-2(5H)-furanones. *J Nat Prod*, **74**, 707-711.
946. Nakamura, M., Suzuki, T., Takagi, M. *et al.* (2014) Stimulation of phosphorylation of ERK and CREB by phellopterin and auraptene isolated from Citrusjunos. *Nat Prod Commun*, **9**, 1491-1494.
947. Nakamura, S., Nakashima, S., Oda, Y. *et al.* (2013) Alkaloids from Sri Lankan curry-leaf (Murraya koenigii) display melanogenesis inhibitory activity: structures of karapinchamines A and B. *Bioorg Med Chem*, **21**, 1043-1049.
948. Nakamura, S., Nakashima, S., Tanabe, G. *et al.* (2013) Alkaloid constituents from flower buds and leaves of sacred lotus (Nelumbo nucifera, Nymphaeaceae) with melanogenesis inhibitory activity in B16 melanoma cells. *Bioorg Med Chem*, **21**, 779-787.
949. Nakane, R. and Iwashina, T. (2015) Flavonol Glycosides from the Leaves of Allium macrostemon. *Nat Prod Commun*, **10**, 1381-1382.
950. Nakashima, S., Matsuda, H., Oda, Y. *et al.* (2010) Melanogenesis inhibitors from the desert plant Anastatica hierochuntica in B16 melanoma cells. *Bioorg Med Chem*, **18**, 2337-2345.
951. Nakashima, S., Oda, Y., Nakamura, S. *et al.* (2015) Inhibitors of melanogenesis in B16 melanoma 4A5 cells from flower buds of Lawsonia inermis (Henna). *Bioorg Med Chem Lett*, **25**, 2702-2706.
952. Nakatani, M., Abdelgaleil, S.A., Kassem, S.M. *et al.* (2002) Three new modified limonoids from Khaya senegalensis. *J Nat Prod*, **65**, 1219-1221.
953. Nam, N.H., Kim, Y., You, Y.J. *et al.* (2004) New constituents from Crinum latifolium with inhibitory effects against tube-like formation of human umbilical venous endothelial cells. *Nat Prod Res*, **18**, 485-491.
954. Naman, C.B., Gupta, G., Varikuti, S. *et al.* (2015) Northalrugosidine is a bisbenzyltetrahydroisoquinoline alkaloid from Thalictrum alpinum with in vivo antileishmanial activity. *J Nat Prod*, **78**, 552-556.
955. Neve, J., Leone, P.A., Carroll, A.R. *et al.* (1999) Sideroxylonal C, a new inhibitor of human plasminogen activator inhibitor type-1, from the flowers of Eucalyptus albens. *J Nat Prod*, **62**, 324-326.
956. Ngassapa, O., Soejarto, D.D., Pezzuto, J.M. *et al.* (1994) Quinone-methide triterpenes and salaspermic acid from Kokoona ochracea. *J Nat Prod*, **57**, 1-8.
957. Ngoc, T.M., Khoi, N.M., Ha do, T. *et al.* (2012) Xanthine oxidase inhibitory activity of constituents of Cinnamomum cassia twigs. *Bioorg Med Chem Lett*, **22**, 4625-4628.
958. Ngoc, T.M., Lee, I., Ha do, T. *et al.* (2009) Tyrosinase-inhibitory constituents from the twigs of Cinnamomum cassia. *J Nat Prod*, **72**, 1205-1208.
959. Nguyen, H.T., Lallemand, M.C., Boutefnouchet, S. *et al.* (2009) Antitumor psoropermum xanthones and sarcomelicope acridones: privileged structures implied in DNA alkylation. *J Nat Prod*, **72**, 527-539.
960. Nguyen, H.T., Song, G.Y., Kim, J.A. *et al.* (2010) Dammarane-type saponins from the flower buds of Panax ginseng and their effects on human leukemia cells. *Bioorg Med Chem Lett*, **20**, 309-314.
961. Nguyen, M.T., Awale, S., Tezuka, Y. *et al.* (2004) Staminane- and isopimarane-type diterpenes from Orthosiphon stamineus of Taiwan and their nitric oxide inhibitory activity. *J Nat Prod*, **67**, 654-658.
962. Nguyen, P.H., Le, T.V., Thuong, P.T. *et al.* (2009) Cytotoxic and PTP1B inhibitory activities from Erythrina abyssinica. *Bioorg Med Chem Lett*, **19**, 6745-6749.
963. Nguyen, P.H., Nguyen, T.N., Dao, T.T. *et al.* (2010) AMP-activated protein kinase (AMPK) activation by benzofurans and coumestans isolated from Erythrina abyssinica. *J Nat Prod*, **73**, 598-602.
964. Nguyen, P.H., Yang, J.L., Uddin, M.N. *et al.* (2013) Protein tyrosine phosphatase 1B (PTP1B) inhibitors from Morinda citrifolia (Noni) and their insulin mimetic activity. *J Nat Prod*, **76**, 2080-2087.
965. Nguyen, P.H., Zhao, B.T., Ali, M.Y. *et al.* (2015) Insulin-mimetic selaginellins from Selaginella tamariscina with protein tyrosine phosphatase 1B (PTP1B) inhibitory activity. *J Nat Prod*, **78**, 34-42.

966. Nguyen, P.H., Zhao, B.T., Lee, J.H. *et al.* (2015) Isolation of benzoic and cinnamic acid derivatives from the grains of Sorghum bicolor and their inhibition of lipopolysaccharide-induced nitric oxide production in RAW 264.7 cells. *Food Chem*, **168**, 512-519.
967. Nguyen, Q.C., Nguyen, V.H., Santarsiero, B.D. *et al.* (2004) New 3-O-acyl betulinic acids from Strychnos vanprukii Craib. *J Nat Prod*, **67**, 994-998.
968. Nguyen, T.P., Tran, C.L., Vuong, C.H. *et al.* (2017) Flavonoids with hepatoprotective activity from the leaves of Cleome viscosa L. *Nat Prod Res*, **31**, 2587-2592.
969. Nhiem, N.X., Hien, N.T., Tai, B.H. *et al.* (2015) New ent-kauranes from the fruits of Annona glabra and their inhibitory nitric oxide production in LPS-stimulated RAW264.7 macrophages. *Bioorg Med Chem Lett*, **25**, 254-258.
970. Ni, G. and Yu, D.Q. (2013) [Chemical constituents from rhizomes of Acorus tatarinowii]. *Zhongguo Zhong Yao Za Zhi*, **38**, 569-573.
971. Ni, G., Zhang, Q.J., Zheng, Z.F. *et al.* (2009) 2-Arylbenzofuran Derivatives from Morus cathayana. *J Nat Prod*, **72**, 966-968.
972. Nick, A., Wright, A.D., Sticher, O. *et al.* (1994) Antibacterial triterpenoid acids from Dillenia papuana. *J Nat Prod*, **57**, 1245-1250.
973. Nikiforova, V.J., Kopka, J., Tolstikov, V. *et al.* (2005) Systems rebalancing of metabolism in response to sulfur deprivation, as revealed by metabolome analysis of Arabidopsis plants. *Plant Physiol*, **138**, 304-318.
974. Nissanka, A.P., Karunaratne, V., Bandara, B.M. *et al.* (2001) Antimicrobial alkaloids from Zanthoxylum tetraspermum and caudatum. *Phytochemistry*, **56**, 857-861.
975. Nkengfack, A.E., Mkounga, P., Fomum, Z.T. *et al.* (2002) Globulixanthones A and B, two new cytotoxic xanthones with isoprenoid groups from the root bark of Symphonia globulifera. *J Nat Prod*, **65**, 734-736.
976. Nojima, H., Kimura, I., Chen, F.J. *et al.* (1998) Antihyperglycemic effects of N-containing sugars from Xanthocercis zambesiaca, Morus bombycis, Aglaonema treubii, and Castanospermum australe in streptozotocin-diabetic mice. *J Nat Prod*, **61**, 397-400.
977. Nomura, T., Fukai, T., Matsumoto, J. *et al.* (1982) Constituents of the cultivated mulberry tree. *Planta Med*, **46**, 28-32.
978. Nomura, T., Fukai, T., Shimada, T. *et al.* (1983) [Components of Root Bark of Morus australis.]. *Planta Med*, **49**, 90-94.
979. Nonhebel, H.M. (1986) Measurement of the rates of oxindole-3-acetic acid turnover, and indole-3-acetic acid oxidation in Zea mays seedlings. *J Exp Bot*, **37**, 1691-1697.
980. Novelo, M., Cruz, J.G., Hernandez, L. *et al.* (1993) Cytotoxic constituents from Hyptis verticillata. *J Nat Prod*, **56**, 1728-1736.
981. Nuanyai, T., Chokpaiboon, S., Vilaivan, T. *et al.* (2010) Cytotoxic 3,4-seco-cycloartane triterpenes from the exudate of Gardenia tubifera. *J Nat Prod*, **73**, 51-54.
982. Nugroho, A.E., Hirasawa, Y., Kawahara, N. *et al.* (2009) Bisnicalaterine A, a vobasine-vobasine bisindole alkaloid from Hunteria zeylanica. *J Nat Prod*, **72**, 1502-1506.
983. Nugroho, A.E., Sugai, M., Hirasawa, Y. *et al.* (2011) New antiplasmodial indole alkaloids from Hunteria zeylanica. *Bioorg Med Chem Lett*, **21**, 3417-3419.
984. Nunez, M.J., Guadano, A., Jimenez, I.A. *et al.* (2004) Insecticidal sesquiterpene pyridine alkaloids from Maytenus chiapensis. *J Nat Prod*, **67**, 14-18.
985. Nunez, M.J., Reyes, C.P., Jimenez, I.A. *et al.* (2005) Lupane triterpenoids from Maytenus species. *J Nat Prod*, **68**, 1018-1021.
986. O'Donnell, G., Poeschl, R., Zimhony, O. *et al.* (2009) Bioactive pyridine-N-oxide disulfides from Allium stipitatum. *J Nat Prod*, **72**, 360-365.
987. O'Neill, M.J., Lewis, J.A., Noble, H.M. *et al.* (1998) Isolation of translactone-containing triterpenes with thrombin inhibitory activities from the leaves of Lantana camara. *J Nat Prod*, **61**, 1328-1331.
988. Ochi, T., Shibata, H., Higuti, T. *et al.* (2005) Anti-Helicobacter pylori compounds from Santalum album. *J Nat Prod*, **68**, 819-824.
989. Ochi, T., Takaishi, Y., Kogure, K. *et al.* (2003) Antioxidant activity of a new capsaicin derivative from Capsicum annuum. *J Nat Prod*, **66**, 1094-1096.
990. Ochieng, C.O., Manguro, L.A., Owuor, P.O. *et al.* (2013) Voulkensin C-E, new 11-oxocassane-type diterpenoids and a steroid glycoside from Caesalpinia volkensii stem bark and their antiplasmodial activities. *Bioorg Med Chem Lett*, **23**, 3088-3095.
991. Ohishi, K., Toume, K., Arai, M.A. *et al.* (2015) Coronaridine, an iboga type alkaloid from Tabernaemontana divaricata, inhibits the Wnt signaling pathway by decreasing beta-catenin mRNA expression. *Bioorg Med Chem Lett*, **25**, 3937-3940.

992. Ohsaki, A., Takashima, J., Chiba, N. *et al.* (1999) Microanalysis of a selective potent anti-Helicobacter pylori compound in a Brazilian medicinal plant, Myroxylon peruiferum and the activity of analogues. *Bioorg Med Chem Lett*, **9**, 1109-1112.
993. Ohse, T., Ohba, S., Yamamoto, T. *et al.* (1996) Cyclopentabenzofuran lignan protein synthesis inhibitors from Aglaia odorata. *J Nat Prod*, **59**, 650-652.
994. Oketch-Rabah, H.A., Dossaji, S.F., Christensen, S.B. *et al.* (1997) Antiprotozoal compounds from Asparagus africanus. *J Nat Prod*, **60**, 1017-1022.
995. Oliva, A., Meepagala, K.M., Wedge, D.E. *et al.* (2003) Natural fungicides from Ruta graveolens L. leaves, including a new quinolone alkaloid. *J Agric Food Chem*, **51**, 890-896.
996. Ono, M., Nishida, Y., Masuoka, C. *et al.* (2004) Lignan derivatives and a norditerpene from the seeds of Vitex negundo. *J Nat Prod*, **67**, 2073-2075.
997. Ono, M., Yanaka, T., Yamamoto, M. *et al.* (2002) New diterpenes and norditerpenes from the fruits of Vitex rotundifolia. *J Nat Prod*, **65**, 537-541.
998. Orabi, K.Y., Mossa, J.S. and el-Feraly, F.S. (1991) Isolation and characterization of two antimicrobial agents from mace (Myristica fragrans). *J Nat Prod*, **54**, 856-859.
999. Orabi, M.A., Taniguchi, S., Sakagami, H. *et al.* (2013) Hydrolyzable tannins of tamaricaceous plants. V. Structures of monomeric-trimeric tannins and cytotoxicity of macrocyclic-type tannins isolated from Tamarix nilotica (1). *J Nat Prod*, **76**, 947-956.
1000. Orabi, M.A., Taniguchi, S., Yoshimura, M. *et al.* (2010) Hydrolyzable tannins of tamaricaceous plants. III. Hellinoyl- and macrocyclic-type ellagitannins from Tamarix nilotica. *J Nat Prod*, **73**, 870-879.
1001. Oramas-Royo, S.M., Chavez, H., Martin-Rodiguez, P. *et al.* (2010) Cytotoxic triterpenoids from Maytenus retusa. *J Nat Prod*, **73**, 2029-2034.
1002. Oshimi, S., Deguchi, J., Hirasawa, Y. *et al.* (2009) Cassiarins C-E, antiplasmodial alkaloids from the flowers of Cassia siamea. *J Nat Prod*, **72**, 1899-1901.
1003. Oshimi, S., Tomizawa, Y., Hirasawa, Y. *et al.* (2008) Chrobisiamone A, a new bischromone from Cassia siamea and a biomimetic transformation of 5-acetonyl-7-hydroxy-2-methylchromone into cassiarin A. *Bioorg Med Chem Lett*, **18**, 3761-3763.
1004. Pan, E., Harinantenaina, L., Brodie, P.J. *et al.* (2010) Four diphenylpropanes and a cycloheptadibenzofuran from Bussea sakalava from the Madagascar dry forest. *J Nat Prod*, **73**, 1792-1795.
1005. Pan, H., Fang, C., Zhou, T. *et al.* (2007) Accumulation of calycosin and its 7-O-beta-D-glucoside and related gene expression in seedlings of Astragalus membranaceus Bge. var. mongholicus (Bge.) Hsiao induced by low temperature stress. *Plant Cell Rep*, **26**, 1111-1120.
1006. Pan, L., Acuna, U.M., Li, J. *et al.* (2013) Bioactive flavaglines and other constituents isolated from Aglaia perviridis. *J Nat Prod*, **76**, 394-404.
1007. Pan, L., Chin, Y.W., Chai, H.B. *et al.* (2009) Bioactivity-guided isolation of cytotoxic constituents of Brucea javanica collected in Vietnam. *Bioorg Med Chem*, **17**, 2219-2224.
1008. Pan, L., Kardono, L.B., Riswan, S. *et al.* (2010) Isolation and characterization of minor analogues of silvestrol and other constituents from a large-scale re-collection of Aglaia foveolata. *J Nat Prod*, **73**, 1873-1878.
1009. Pan, L., Matthew, S., Lantvit, D.D. *et al.* (2011) Bioassay-guided isolation of constituents of Piper sarmentosum using a mitochondrial transmembrane potential assay. *J Nat Prod*, **74**, 2193-2199.
1010. Pan, L.L., Fang, P.L., Zhang, X.J. *et al.* (2011) Tigliane-type diterpenoid glycosides from Euphorbia fischeriana. *J Nat Prod*, **74**, 1508-1512.
1011. Pan, Q.M., Li, Y.H., Hua, J. *et al.* (2015) Antiviral Matrine-Type Alkaloids from the Rhizomes of Sophora tonkinensis. *J Nat Prod*, **78**, 1683-1688.
1012. Pan, Y., Wang, X. and Hu, X. (2007) Cytotoxic withanolides from the flowers of Datura metel. *J Nat Prod*, **70**, 1127-1132.
1013. Paniego, N.B., Zuurbier, K.W., Fung, S.Y. *et al.* (1999) Phlorisovalerophenone synthase, a novel polyketide synthase from hop (Humulus lupulus L.) cones. *Eur J Biochem*, **262**, 612-616.
1014. Panza, E., Tersigni, M., Iorizzi, M. *et al.* (2011) Lauroside B, a megastigmane glycoside from Laurus nobilis (bay laurel) leaves, induces apoptosis in human melanoma cell lines by inhibiting NF-kappaB activation. *J Nat Prod*, **74**, 228-233.
1015. Pari, K., Rao, P.J., Devakumar, C. *et al.* (1998) A Novel Insect Antifeedant Nonprotein Amino Acid from Calotropis gigantea. *J Nat Prod*, **61**, 102-104.
1016. Paris, A., Strukelj, B., Renko, M. *et al.* (1993) Inhibitory effect of carnosic acid on HIV-1 protease in cell-free assays [corrected]. *J Nat Prod*, **56**, 1426-1430.
1017. Park, B.H., Lee, H.J. and Lee, Y.R. (2011) Total synthesis of chiricanine A, arahypin-1, trans-arachidin-2, trans-arachidin-3, and arahypin-5 from peanut seeds. *J Nat Prod*, **74**, 644-649.

1018. Park, B.S., Kim, D.Y., Rosenthal, P.J. *et al.* (2002) Synthesis and evaluation of new antimalarial analogues of quinoline alkaloids derived from Cinchona ledgeriana Moens ex Trimen. *Bioorg Med Chem Lett*, **12**, 1351-1355.
1019. Park, H.J., Jung, W.T., Basnet, P. *et al.* (1996) Syringin 4-O-beta-glucoside, a new phenylpropanoid glycoside, and costunolide, a nitric oxide synthase inhibitor, from the stem bark of Magnolia sieboldii. *J Nat Prod*, **59**, 1128-1130.
1020. Park, H.J., Kwon, S.H., Han, Y.N. *et al.* (2001) Apoptosis-Inducing costunolide and a novel acyclic monoterpene from the stem bark of Magnolia sieboldii. *Arch Pharm Res*, **24**, 342-348.
1021. Park, J.J., Wang, H., Gargouri, M. *et al.* (2015) The response of Chlamydomonas reinhardtii to nitrogen deprivation: a systems biology analysis. *Plant J*, **81**, 611-624.
1022. Park, J.Y., Jeong, H.J., Kim, Y.M. *et al.* (2011) Characteristic of alkylated chalcones from Angelica keiskei on influenza virus neuraminidase inhibition. *Bioorg Med Chem Lett*, **21**, 5602-5604.
1023. Park, K.H., Park, Y.D., Han, J.M. *et al.* (2006) Anti-atherosclerotic and anti-inflammatory activities of catecholic xanthones and flavonoids isolated from Cudrania tricuspidata. *Bioorg Med Chem Lett*, **16**, 5580-5583.
1024. Park, S., Nhiem, N.X., Kiem, P.V. *et al.* (2014) Five new quassinoids and cytotoxic constituents from the roots of Eurycoma longifolia. *Bioorg Med Chem Lett*, **24**, 3835-3840.
1025. Park, S.Y. and Kim, D.S. (2002) Discovery of natural products from Curcuma longa that protect cells from beta-amyloid insult: a drug discovery effort against Alzheimer's disease. *J Nat Prod*, **65**, 1227-1231.
1026. Park, S.Y., Seetharaman, R., Ko, M.J. *et al.* (2014) Ethyl linoleate from garlic attenuates lipopolysaccharide-induced pro-inflammatory cytokine production by inducing heme oxygenase-1 in RAW264.7 cells. *Int Immunopharmacol*, **19**, 253-261.
1027. Parry, A.D., Tiller, S.A. and Edwards, R. (1994) The Effects of Heavy Metals and Root Immersion on Isoflavonoid Metabolism in Alfalfa (Medicago sativa L.). *Plant Physiol*, **106**, 195-202.
1028. Patel, N.K. and Bhutani, K.K. (2014) Pinostrobin and Cajanus lactone isolated from Cajanus cajan (L.) leaves inhibits TNF-alpha and IL-1beta production: in vitro and in vivo experimentation. *Phytomedicine*, **21**, 946-953.
1029. Patil, A.D., Freyer, A.J., Eggleston, D.S. *et al.* (1993) The inophyllums, novel inhibitors of HIV-1 reverse transcriptase isolated from the Malaysian tree, Calophyllum inophyllum Linn. *J Med Chem*, **36**, 4131-4138.
1030. Patil, A.D., Freyer, A.J., Killmer, L. *et al.* (2002) A new dimeric dihydrochalcone and a new prenylated flavone from the bud covers of Artocarpus altilis: potent inhibitors of cathepsin K. *J Nat Prod*, **65**, 624-627.
1031. Pawlus, A.D., Su, B.N., Keller, W.J. *et al.* (2005) An anthraquinone with potent quinone reductase-inducing activity and other constituents of the fruits of Morinda citrifolia (noni). *J Nat Prod*, **68**, 1720-1722.
1032. Pencik, A., Simonovik, B., Petersson, S.V. *et al.* (2013) Regulation of auxin homeostasis and gradients in Arabidopsis roots through the formation of the indole-3-acetic acid catabolite 2-oxindole-3-acetic acid. *Plant Cell*, **25**, 3858-3870.
1033. Peng, A., Li, R., Hu, J. *et al.* (2008) Flow rate gradient high-speed counter-current chromatography separation of five diterpenoids from Triperygium wilfordii and scale-up. *J Chromatogr A*, **1200**, 129-135.
1034. Peng, C.Y., Liu, J.Q., Zhang, R. *et al.* (2014) A new alkaloid from the fruit of Nandina domestica Thunb. *Nat Prod Res*, **28**, 1159-1164.
1035. Peng, K., Yang, L., Zhao, S. *et al.* (2013) Chemical constituents from the fruit of Gardenia jasminoides and their inhibitory effects on nitric oxide production. *Bioorg Med Chem Lett*, **23**, 1127-1131.
1036. Peng, W.W., Li, W., Li, J.S. *et al.* (2013) The effects of Rhizoma Zingiberis on pharmacokinetics of six Aconitum alkaloids in herb couple of Radix Aconiti Lateralis-Rhizoma Zingiberis. *J Ethnopharmacol*, **148**, 579-586.
1037. Pengsuparp, T., Cai, L., Constant, H. *et al.* (1995) Mechanistic evaluation of new plant-derived compounds that inhibit HIV-1 reverse transcriptase. *J Nat Prod*, **58**, 1024-1031.
1038. Perez-Castorena, A.L., Martinez, M. and Maldonado, E. (2010) Labdanes and sucrose esters from Physalis sordida. *J Nat Prod*, **73**, 1271-1276.
1039. Permana, D., Lajis, N.H., Mackeen, M.M. *et al.* (2001) Isolation and bioactivities of constitutents of the roots of Garcinia atroviridis. *J Nat Prod*, **64**, 976-979.
1040. Pettit, G.R., Cragg, G.M. and Singh, S.B. (1987) Antineoplastic agents, 122. Constituents of Combretum caffrum. *J Nat Prod*, **50**, 386-391.
1041. Pettit, G.R., Hogan, F., Xu, J.P. *et al.* (2008) Antineoplastic agents. 536. New sources of naturally occurring cancer cell growth inhibitors from marine organisms, terrestrial plants, and microorganisms(1a,). *J Nat Prod*, **71**, 438-444.

1042. Pettit, G.R., Singh, S.B., Niven, M.L. *et al.* (1987) Isolation, structure, and synthesis of combretastatins A-1 and B-1, potent new inhibitors of microtubule assembly, derived from Combretum caffrum. *J Nat Prod*, **50**, 119-131.
1043. Pettit, G.R., Singh, S.B., Schmidt, J.M. *et al.* (1988) Isolation, structure, synthesis, and antimitotic properties of combretastatins B-3 and B-4 from Combretum caffrum. *J Nat Prod*, **51**, 517-527.
1044. Pettit, G.R., Thornhill, A., Melody, N. *et al.* (2009) Antineoplastic agents. 578. Synthesis of stilstatins 1 and 2 and their water-soluble prodrugs. *J Nat Prod*, **72**, 380-388.
1045. Pettit, G.R., Zhang, Q., Pinilla, V. *et al.* (2005) Antineoplastic agents. 534. isolation and structure of sansevistatins 1 and 2 from the African Sansevieria ehrenbergii. *J Nat Prod*, **68**, 729-733.
1046. Pham, V.C., Ma, J., Thomas, S.J. *et al.* (2005) Alkaloids from Alangium javanicum and Alangium grisolleoides that mediate Cu2+-dependent DNA strand scission. *J Nat Prod*, **68**, 1147-1152.
1047. Phan, V.K., Nguyen, X.C., Nguyen, X.N. *et al.* (2011) Antioxidant activity of a new C-glycosylflavone from the leaves of Ficus microcarpa. *Bioorg Med Chem Lett*, **21**, 633-637.
1048. Phifer, S.S., Lee, D., Seo, E.K. *et al.* (2007) Alvaradoins E-N, antitumor and cytotoxic anthracenone C-glycosides from the leaves of Alvaradoa haitiensis. *J Nat Prod*, **70**, 954-961.
1049. Phommart, S., Sutthivaiyakit, P., Chimnoi, N. *et al.* (2005) Constituents of the leaves of Macaranga tanarius. *J Nat Prod*, **68**, 927-930.
1050. Phuwapraisirisan, P., Puksasook, T., Jong-Aramruang, J. *et al.* (2008) Phenylethyl cinnamides: a new series of alpha-glucosidase inhibitors from the leaves of Aegle marmelos. *Bioorg Med Chem Lett*, **18**, 4956-4958.
1051. Pistelli, L., Noccioli, C., Appendino, G. *et al.* (2003) Pterocarpans from Bituminaria morisiana and Bituminaria bituminosa. *Phytochemistry*, **64**, 595-598.
1052. Polavarapu, P.L., Scalmani, G., Hawkins, E.K. *et al.* (2011) Importance of solvation in understanding the chiroptical spectra of natural products in solution phase: garcinia acid dimethyl ester. *J Nat Prod*, **74**, 321-328.
1053. Pollastro, F., Taglialatela-Scafati, O., Allara, M. *et al.* (2011) Bioactive prenylogous cannabinoid from fiber hemp (Cannabis sativa). *J Nat Prod*, **74**, 2019-2022.
1054. Ponnapalli, M.G., Annam, S., Ravirala, S. *et al.* (2012) Unusual isomeric corniculatolides from mangrove, Aegiceras corniculatum. *J Nat Prod*, **75**, 275-279.
1055. Pozharski, E., Wilson, M.A., Hewagama, A. *et al.* (2004) Anchoring a cationic ligand: the structure of the Fab fragment of the anti-morphine antibody 9B1 and its complex with morphine. *J Mol Biol*, **337**, 691-697.
1056. Prabhakar Reddy, P., Tiwari, A.K., Ranga Rao, R. *et al.* (2009) New Labdane diterpenes as intestinal alpha-glucosidase inhibitor from antihyperglycemic extract of Hedychium spicatum (Ham. Ex Smith) rhizomes. *Bioorg Med Chem Lett*, **19**, 2562-2565.
1057. Prakash Chaturvedula, V.S., Schilling, J.K., Johnson, R.K. *et al.* (2003) New cytotoxic lupane triterpenoids from the twigs of Coussarea paniculata. *J Nat Prod*, **66**, 419-422.
1058. Prakash Chaturvedula, V.S., Schilling, J.K., Miller, J.S. *et al.* (2003) New cytotoxic alkaloids from the wood of Vepris punctata from the Madagascar rainforest. *J Nat Prod*, **66**, 532-534.
1059. Prakash Chaturvedula, V.S., Sprague, S., Schilling, J.K. *et al.* (2003) New cytotoxic indole alkaloids from Tabernaemontana calcarea from the Madagascar rainforest. *J Nat Prod*, **66**, 528-531.
1060. Procopio, A., Celia, C., Nardi, M. *et al.* (2011) Lipophilic hydroxytyrosol esters: fatty acid conjugates for potential topical administration. *J Nat Prod*, **74**, 2377-2381.
1061. Pudhom, K., Sommit, D., Nuclear, P. *et al.* (2010) Moluccensins H-J, 30-ketophragmalin limonoids from Xylocarpus moluccensis. *J Nat Prod*, **73**, 263-266.
1062. Pudhom, K., Sommit, D., Nuclear, P. *et al.* (2009) Protoxylocarpins F-H, protolimonoids from seed kernels of Xylocarpus granatum. *J Nat Prod*, **72**, 2188-2191.
1063. Pungitore, C.R., Ayub, M.J., Garcia, M. *et al.* (2004) Iridoids as allelochemicals and DNA polymerase inhibitors. *J Nat Prod*, **67**, 357-361.
1064. Puttarak, P. and Panichayupakaranant, P. (2012) Factors affecting the content of pentacyclic triterpenes in Centella asiatica raw materials. *Pharm Biol*, **50**, 1508-1512.
1065. Pyee, Y., Chung, H.J., Choi, T.J. *et al.* (2014) Suppression of inflammatory responses by handelin, a guaianolide dimer from Chrysanthemum boreale, via downregulation of NF-kappaB signaling and pro-inflammatory cytokine production. *J Nat Prod*, **77**, 917-924.
1066. Qian, C.D., Jiang, F.S., Yu, H.S. *et al.* (2015) Antibacterial Biphenanthrenes from the Fibrous Roots of Bletilla striata. *J Nat Prod*, **78**, 939-943.
1067. Qiao, L., Yang, L., Zhang, D. *et al.* (2011) [Studies on chemical constitutes from callus cultures of Stellera chamaejasme]. *Zhongguo Zhong Yao Za Zhi*, **36**, 3457-3462.
1068. Qin, J.J., Zhu, J.X., Zeng, Q. *et al.* (2011) Pseudoguaianolides and guaianolides from Inula hupehensis as potential anti-inflammatory agents. *J Nat Prod*, **74**, 1881-1887.

1069. Quang, T.H., Ngan, N.T., Minh, C.V. *et al.* (2012) Anti-inflammatory and PPAR transactivational effects of secondary metabolites from the roots of Asarum sieboldii. *Bioorg Med Chem Lett*, **22**, 2527-2533.
1070. Quang, T.H., Ngan, N.T., Minh, C.V. *et al.* (2012) Diarylheptanoid glycosides from Tacca plantaginea and their effects on NF-kappaB activation and PPAR transcriptional activity. *Bioorg Med Chem Lett*, **22**, 6681-6687.
1071. Queiroz, E.F., Atindehou, K.K., Terreaux, C. *et al.* (2002) Prenylated isoflavonoids from the root bark of Erythrina vogelii. *J Nat Prod*, **65**, 403-406.
1072. Queiroz, S.C., Cantrell, C.L., Duke, S.O. *et al.* (2012) Bioassay-directed isolation and identification of phytotoxic and fungitoxic acetylenes from Conyza canadensis. *J Agric Food Chem*, **60**, 5893-5898.
1073. Quinhone Junior, A. and Ida, E.I. (2014) Isoflavones of the soybean components and the effect of germination time in the cotyledons and embryonic axis. *J Agric Food Chem*, **62**, 8452-8459.
1074. Quinhone Junior, A. and Ida, E.I. (2015) Profile of the contents of different forms of soybean isoflavones and the effect of germination time on these compounds and the physical parameters in soybean sprouts. *Food Chem*, **166**, 173-178.
1075. Radulovic, N., Denic, M. and Stojanovic-Radic, Z. (2010) Antimicrobial phenolic abietane diterpene from Lycopus europaeus L. (Lamiaceae). *Bioorg Med Chem Lett*, **20**, 4988-4991.
1076. Radwan, M.M., ElSohly, M.A., El-Alfy, A.T. *et al.* (2015) Isolation and Pharmacological Evaluation of Minor Cannabinoids from High-Potency Cannabis sativa. *J Nat Prod*, **78**, 1271-1276.
1077. Radwan, M.M., Elsohly, M.A., Slade, D. *et al.* (2009) Biologically active cannabinoids from high-potency Cannabis sativa. *J Nat Prod*, **72**, 906-911.
1078. Raffa, R.B., Beckett, J.R., Brahmbhatt, V.N. *et al.* (2013) Orally active opioid compounds from a non-poppy source. *J Med Chem*, **56**, 4840-4848.
1079. Rahman, A., Anjum, S., Farooq, A. *et al.* (1998) Antibacterial steroidal alkaloids from Sarcococca saligna. *J Nat Prod*, **61**, 202-206.
1080. Rai, A., Umashankar, S., Rai, M. *et al.* (2016) Coordinate Regulation of Metabolite Glycosylation and Stress Hormone Biosynthesis by TT8 in Arabidopsis. *Plant Physiol*, **171**, 2499-2515.
1081. Ramesh, N., Viswanathan, M.B., Saraswathy, A. *et al.* (2002) Antimicrobial and phytochemical studies of Swertia corymbosa. *Fitoterapia*, **73**, 160-164.
1082. Ranga Rao, R., Tiwari, A.K., Prabhakar Reddy, P. *et al.* (2009) New furanoflavanoids, intestinal alpha-glucosidase inhibitory and free-radical (DPPH) scavenging, activity from antihyperglycemic root extract of Derris indica (Lam.). *Bioorg Med Chem*, **17**, 5170-5175.
1083. Rastrelli, L., Capasso, A., Pizza, C. *et al.* (1997) New protopine and benzyltetrahydroprotoberberine alkaloids from Aristolochia constricta and their activity on isolated guinea-pig ileum. *J Nat Prod*, **60**, 1065-1069.
1084. Ratnayake, S., Fang, X.P., Anderson, J.E. *et al.* (1992) Bioactive constituents from the twigs of Asimina parviflora. *J Nat Prod*, **55**, 1462-1467.
1085. Rauws, A.G., Olling, M. and Timmerman, A. (1982) The pharmacokinetics of amygdalin. *Arch Toxicol*, **49**, 311-319.
1086. Ravangpai, W., Sommit, D., Teerawatananond, T. *et al.* (2011) Limonoids from seeds of Thai Xylocarpus moluccensis. *Bioorg Med Chem Lett*, **21**, 4485-4489.
1087. Reddy, P.P., Rao, R.R., Rekha, K. *et al.* (2009) Two new cytotoxic diterpenes from the rhizomes of Hedychium spicatum. *Bioorg Med Chem Lett*, **19**, 192-195.
1088. Reddy, P.P., Rao, R.R., Shashidhar, J. *et al.* (2009) Phytochemical investigation of labdane diterpenes from the rhizomes of Hedychium spicatum and their cytotoxic activity. *Bioorg Med Chem Lett*, **19**, 6078-6081.
1089. Ren, J., Wang, Y.G., Wang, A.G. *et al.* (2015) Cembranoids from the Gum Resin of Boswellia carterii as Potential Antiulcerative Colitis Agents. *J Nat Prod*, **78**, 2322-2331.
1090. Ren, Y., Matthew, S., Lantvit, D.D. *et al.* (2011) Cytotoxic and NF-kappaB inhibitory constituents of the stems of Cratoxylum cochinchinense and their semisynthetic analogues. *J Nat Prod*, **74**, 1117-1125.
1091. Resch, M., Steigel, A., Chen, Z.L. *et al.* (1998) 5-Lipoxygenase and cyclooxygenase-1 inhibitory active compounds from Atractylodes lancea. *J Nat Prod*, **61**, 347-350.
1092. Rho, M.C., Kwon, O.E., Kim, K. *et al.* (2003) Inhibitory effects of manassantin A and B isolated from the roots of Saururus chinensis on PMA-induced ICAM-1 expression. *Planta Med*, **69**, 1147-1149.
1093. Rieser, M.J., Gu, Z.M., Fang, X.P. *et al.* (1996) Five novel mono-tetrahydrofuran ring acetogenins from the seeds of Annona muricata. *J Nat Prod*, **59**, 100-108.
1094. Rifai, Y., Arai, M.A., Sadhu, S.K. *et al.* (2011) New Hedgehog/GLI signaling inhibitors from Excoecaria agallocha. *Bioorg Med Chem Lett*, **21**, 718-722.
1095. Rimando, A.M., Dayan, F.E., Czarnota, M.A. *et al.* (1998) A new photosystem II electron transfer inhibitor from Sorghum bicolor. *J Nat Prod*, **61**, 927-930.

1096. Rimando, A.M., Dayan, F.E. and Streibig, J.C. (2003) PSII inhibitory activity of resorcinolic lipids from Sorghum bicolor. *J Nat Prod*, **66**, 42-45.
1097. Ringbom, T., Huss, U., Stenholm, A. *et al.* (2001) Cox-2 inhibitory effects of naturally occurring and modified fatty acids. *J Nat Prod*, **64**, 745-749.
1098. Rios, M.Y., Salina, D. and Villarreal, M.L. (2001) Cytotoxic activity of moronic acid and identification of the new triterpene 3,4-seco-olean-18-ene-3,28-dioic acid from Phoradendron reichenbachianum. *Planta Med*, **67**, 443-446.
1099. Rivero-Cruz, J.F., Chai, H.B., Kardono, L.B. *et al.* (2004) Cytotoxic constituents of the twigs and leaves of Aglaia rubiginosa. *J Nat Prod*, **67**, 343-347.
1100. Robles, A.J., Peng, J., Hartley, R.M. *et al.* (2015) Melampodium leucanthum, a source of cytotoxic sesquiterpenes with antimitotic activities. *J Nat Prod*, **78**, 388-395.
1101. Rochfort, S.J., Towerzey, L., Carroll, A. *et al.* (2005) Latifolians A and B, novel JNK3 kinase inhibitors from the Papua New Guinean plant Gnetum latifolium. *J Nat Prod*, **68**, 1080-1082.
1102. Rodriguez, N., Vasquez, Y., Hussein, A.A. *et al.* (2003) Cytotoxic cucurbitacin constituents from Sloanea zuliaensis. *J Nat Prod*, **66**, 1515-1516.
1103. Rojas-Flores, C., Rios, M.Y., Lopez-Marure, R. *et al.* (2014) Karwinaphthopyranones from the fruits of Karwinskia parvifolia and their cytotoxic activities. *J Nat Prod*, **77**, 2404-2409.
1104. Rolfsen, W.N., Olaniyi, A.A. and Hylands, P.J. (1980) New tertiary alkaloids of Strychnos decussata. *J Nat Prod*, **43**, 97-102.
1105. Rollinger, J.M., Kratschmar, D.V., Schuster, D. *et al.* (2010) 11beta-Hydroxysteroid dehydrogenase 1 inhibiting constituents from Eriobotrya japonica revealed by bioactivity-guided isolation and computational approaches. *Bioorg Med Chem*, **18**, 1507-1515.
1106. Roman, M., Dobrowolski, J.C., Baranska, M. *et al.* (2011) Spectroscopic studies on bioactive polyacetylenes and other plant components in wild carrot root. *J Nat Prod*, **74**, 1757-1763.
1107. Romero, L.C., Aroca, M.A., Laureano-Marin, A.M. *et al.* (2014) Cysteine and cysteine-related signaling pathways in Arabidopsis thaliana. *Mol Plant*, **7**, 264-276.
1108. Ross, S.A., Al-Azeib, M.A., Krishnaveni, K.S. *et al.* (2005) Alkamides from the leaves of Zanthoxylum syncarpum. *J Nat Prod*, **68**, 1297-1299.
1109. Ross, S.A., Sultana, G.N., Burandt, C.L. *et al.* (2004) Syncarpamide, a new antiplasmodial (+)-norepinephrine derivative from Zanthoxylum syncarpum. *J Nat Prod*, **67**, 88-90.
1110. Roth, G.N., Chandra, A. and Nair, M.G. (1998) Novel bioactivities of Curcuma longa constituents. *J Nat Prod*, **61**, 542-545.
1111. Roumy, V., Biabiany, M., Hennebelle, T. *et al.* (2010) Antifungal and cytotoxic activity of withanolides from Acnistus arborescens. *J Nat Prod*, **73**, 1313-1317.
1112. Roux, D., Hadi, H.A., Thoret, S. *et al.* (2000) Structure-activity relationship of polyisoprenyl benzophenones from Garcinia pyrifera on the tubulin/microtubule system. *J Nat Prod*, **63**, 1070-1076.
1113. Row, L.C., Ho, J.C. and Chen, C.M. (2007) Cerebrosides and tocopherol trimers from the seeds of Euryale ferox. *J Nat Prod*, **70**, 1214-1217.
1114. Roy, M.C., Chang, F.R., Huang, H.C. *et al.* (2005) Cytotoxic principles from the formosan milkweed, Asclepias curassavica. *J Nat Prod*, **68**, 1494-1499.
1115. Ruangrungsi, N., Likhitwitayawuid, K., Kasiwong, S. *et al.* (1988) Constituents of Michelia Rajaniana. Two new germacranolide amides. *J Nat Prod*, **51**, 1220-1225.
1116. Rubnov, S., Kashman, Y., Rabinowitz, R. *et al.* (2001) Suppressors of cancer cell proliferation from fig (Ficus carica) resin: isolation and structure elucidation. *J Nat Prod*, **64**, 993-996.
1117. Rudrapaul, P., Sarma, I.S., Das, N. *et al.* (2014) New flavonol methyl ether from the leaves of Vitex peduncularis exhibits potential inhibitory activity against Leishmania donovani through activation of iNOS expression. *Eur J Med Chem*, **87**, 328-335.
1118. Rukachaisirikul, V., Saelim, S., Karnsomchoke, P. *et al.* (2005) Friedolanostanes and lanostanes from the leaves of Garcinia hombroniana. *J Nat Prod*, **68**, 1222-1225.
1119. Ryu, J.H., Jeong, Y.S. and Sohn, D.H. (1999) A new bisabolene epoxide from Tussilago farfara, and inhibition of nitric oxide synthesis in LPS-activated macrophages. *J Nat Prod*, **62**, 1437-1438.
1120. Ryu, Y.B., Curtis-Long, M.J., Lee, J.W. *et al.* (2009) Characteristic of neuraminidase inhibitory xanthones from Cudrania tricuspidata. *Bioorg Med Chem*, **17**, 2744-2750.
1121. Ryu, Y.B., Curtis-Long, M.J., Lee, J.W. *et al.* (2009) Structural characteristics of flavanones and flavones from Cudrania tricuspidata for neuraminidase inhibition. *Bioorg Med Chem Lett*, **19**, 4912-4915.
1122. Ryu, Y.B., Park, S.J., Kim, Y.M. *et al.* (2010) SARS-CoV 3CLpro inhibitory effects of quinone-methide triterpenes from Tripterygium regelii. *Bioorg Med Chem Lett*, **20**, 1873-1876.
1123. Ryu, Y.B., Westwood, I.M., Kang, N.S. *et al.* (2008) Kurarinol, tyrosinase inhibitor isolated from the root of Sophora flavescens. *Phytomedicine*, **15**, 612-618.

1124. Sa, M.S., de Menezes, M.N., Krettli, A.U. *et al.* (2011) Antimalarial activity of physalins B, D, F, and G. *J Nat Prod*, **74**, 2269-2272.
1125. Sadhu, S.K., Okuyama, E., Fujimoto, H. *et al.* (2006) Diterpenes from Leucas aspera inhibiting prostaglandin-induced contractions. *J Nat Prod*, **69**, 988-994.
1126. Safaralie, A., Fatemi, S. and Sefidkon, F. (2008) Essential oil composition of Valeriana officinalis L. roots cultivated in Iran. Comparative analysis between supercritical CO2 extraction and hydrodistillation. *J Chromatogr A*, **1180**, 159-164.
1127. Sagawa, T., Takaishi, Y., Fujimoto, Y. *et al.* (2005) Cyclobutane dimers from the Colombian medicinal plant Achyrocline bogotensis. *J Nat Prod*, **68**, 502-505.
1128. Saifudin, A., Tanaka, K., Kadota, S. *et al.* (2013) Sesquiterpenes from the rhizomes of Curcuma heyneana. *J Nat Prod*, **76**, 223-229.
1129. Sairafianpour, M., Christensen, J., Staerk, D. *et al.* (2001) Leishmanicidal, antiplasmodial, and cytotoxic activity of novel diterpenoid 1,2-quinones from Perovskia abrotanoides: new source of tanshinones. *J Nat Prod*, **64**, 1398-1403.
1130. Sairafianpour, M., Kayser, O., Christensen, J. *et al.* (2002) Leishmanicidal and antiplasmodial activity of constituents of Smirnowia iranica. *J Nat Prod*, **65**, 1754-1758.
1131. Sakakibara, N., Suzuki, S., Umezawa, T. *et al.* (2003) Biosynthesis of yatein in Anthriscus sylvestris. *Org Biomol Chem*, **1**, 2474-2485.
1132. Sakurai, N., Wu, J.H., Sashida, Y. *et al.* (2004) Anti-AIDS agents. Part 57: Actein, an anti-HIV principle from the rhizome of Cimicifuga racemosa (black cohosh), and the anti-HIV activity of related saponins. *Bioorg Med Chem Lett*, **14**, 1329-1332.
1133. Sakurai, Y., Sakurai, N., Taniguchi, M. *et al.* (2006) Rautandiols A and B, pterocarpans and cytotoxic constituents from Neorautanenia mitis. *J Nat Prod*, **69**, 397-399.
1134. Sala, A., Recio, M.C., Giner, R.M. *et al.* (2001) New acetophenone glucosides isolated from extracts of Helichrysum italicum with antiinflammatory activity. *J Nat Prod*, **64**, 1360-1362.
1135. Salem, M.M. and Werbovetz, K.A. (2005) Antiprotozoal compounds from Psorothamnus polydenius. *J Nat Prod*, **68**, 108-111.
1136. Salem, M.M. and Werbovetz, K.A. (2006) Isoflavonoids and other compounds from Psorothamnus arborescens with antiprotozoal activities. *J Nat Prod*, **69**, 43-49.
1137. Sang, S., Lao, A., Wang, Y. *et al.* (2002) Antifungal constituents from the seeds of Allium fistulosum L. *J Agric Food Chem*, **50**, 6318-6321.
1138. Sansone-Land, A., Takeoka, G.R. and Shoemaker, C.F. (2014) Volatile constituents of commercial imported and domestic black-ripe table olives (Olea europaea). *Food Chem*, **149**, 285-295.
1139. Sanyar, G., Gulgeze, H.B. and Gozler, B. (1992) Salutaridine N-Oxide from the Capsules of Papaver bracteatum. *Planta Med*, **58**, 368-369.
1140. Saroglou, V., Karioti, A., Demetzos, C. *et al.* (2005) Sesquiterpene lactones from Centaurea spinosa and their antibacterial and cytotoxic activities. *J Nat Prod*, **68**, 1404-1407.
1141. Sashidhara, K.V., Singh, S.P., Misra, S. *et al.* (2012) Galactolipids from Bauhinia racemosa as a new class of antifilarial agents against human lymphatic filarial parasite, Brugia malayi. *Eur J Med Chem*, **50**, 230-235.
1142. Sashidhara, K.V., Singh, S.P., Singh, S.V. *et al.* (2013) Isolation and identification of beta-hematin inhibitors from Flacourtia indica as promising antiplasmodial agents. *Eur J Med Chem*, **60**, 497-502.
1143. Sathiamoorthy, B., Gupta, P., Kumar, M. *et al.* (2007) New antifungal flavonoid glycoside from Vitex negundo. *Bioorg Med Chem Lett*, **17**, 239-242.
1144. Schenk, B., Junior, P. and Wichtl, M. (1980) [Cannogenol-3-O-alpha-L-rhamnoside and cannogenol-3-O-beta-D-allomethyloside, two new cardiac glycosides from Convallaria majalis (author's transl)]. *Planta Med*, **40**, 1-11.
1145. Schinella, G., Aquila, S., Dade, M. *et al.* (2008) Anti-inflammatory and apoptotic activities of pomolic acid isolated from Cecropia pachystachya. *Planta Med*, **74**, 215-220.
1146. Schmidt, C.A., Murillo, R., Heinzmann, B. *et al.* (2011) Structural and conformational analysis of proanthocyanidins from Parapiptadenia rigida and their wound-healing properties. *J Nat Prod*, **74**, 1427-1436.
1147. Schmidt, T.J., Stausberg, S., Raison, J.V. *et al.* (2006) Lignans from Arnica species. *Nat Prod Res*, **20**, 443-453.
1148. Schomburg, C., Schuehly, W., Da Costa, F.B. *et al.* (2013) Natural sesquiterpene lactones as inhibitors of Myb-dependent gene expression: structure-activity relationships. *Eur J Med Chem*, **63**, 313-320.
1149. Schuhly, W., Skarbina, J., Kunert, O. *et al.* (2009) Chemical characterization of Magnolia biondii (Flos Magnoliae, Xin Yi). *Nat Prod Commun*, **4**, 231-234.

1150. Schumacher, B., Scholle, S., Holzl, J. *et al.* (2002) Lignans isolated from valerian: identification and characterization of a new olivil derivative with partial agonistic activity at A(1) adenosine receptors. *J Nat Prod*, **65**, 1479-1485.
1151. Seephonkai, P., Popescu, R., Zehl, M. *et al.* (2011) Ferruginenes A-C from Rhododendron ferrugineum and their cytotoxic evaluation. *J Nat Prod*, **74**, 712-717.
1152. Sekhon, J.K., Maness, N.O. and Jones, C.L. (2015) Effect of preprocessing and compressed propane extraction on quality of cilantro (Coriandrum sativum L.). *Food Chem*, **175**, 322-328.
1153. Selenge, E., Murata, T., Kobayashi, K. *et al.* (2013) Flavone tetraglycosides and benzyl alcohol glycosides from the Mongolian medicinal plant Dracocephalum ruyschiana. *J Nat Prod*, **76**, 186-193.
1154. Semenov, V.V., Kiselyov, A.S., Titov, I.Y. *et al.* (2010) Synthesis of antimitotic polyalkoxyphenyl derivatives of combretastatin using plant allylpolyalkoxybenzenes. *J Nat Prod*, **73**, 1796-1802.
1155. Sendl, A., Chen, J.L., Jolad, S.D. *et al.* (1996) Two new naphthoquinones with antiviral activity from Rhinacanthus nasutus. *J Nat Prod*, **59**, 808-811.
1156. Seo, C., Choi, Y.H., Sohn, J.H. *et al.* (2008) Ohioensins F and G: protein tyrosine phosphatase 1B inhibitory benzonaphthoxanthenones from the Antarctic moss Polytrichastrum alpinum. *Bioorg Med Chem Lett*, **18**, 772-775.
1157. Seo, C.S., Zheng, M.S., Woo, M.H. *et al.* (2008) Lignans from the roots of Saururus chinensis. *J Nat Prod*, **71**, 1771-1774.
1158. Seo, E.J., Curtis-Long, M.J., Lee, B.W. *et al.* (2007) Xanthones from Cudrania tricuspidata displaying potent alpha-glucosidase inhibition. *Bioorg Med Chem Lett*, **17**, 6421-6424.
1159. Sevimli-Gur, C., Akgun, I.H., Deliloglu-Gurhan, I. *et al.* (2010) Cytotoxic naphthoquinones from Alkanna cappadocica (perpendicular). *J Nat Prod*, **73**, 860-864.
1160. Shen, C.C., Lin, T.W., Huang, Y.L. *et al.* (2006) Phenolic constituents of the roots of Sophora flavescens. *J Nat Prod*, **69**, 1237-1240.
1161. Shen, C.C., Syu, W.J., Li, S.Y. *et al.* (2002) Antimicrobial activities of naphthazarins from Arnebia euchroma. *J Nat Prod*, **65**, 1857-1862.
1162. Shen, C.C., Wang, S.T., Tsai, S.Y. *et al.* (2005) Cinnamylphenols from Phyllodium pulchellum. *J Nat Prod*, **68**, 791-793.
1163. Shen, Y.C. and Chen, C.H. (1994) Alkaloids from Lycopodium casuarinoides. *J Nat Prod*, **57**, 824-826.
1164. Shen, Y.C., Cheng, Y.B., Ahmed, A.F. *et al.* (2005) Cytotoxic clerodane diterpenoids from Casearia membranacea. *J Nat Prod*, **68**, 1665-1668.
1165. Shen, Y.H., Li, R.T., Xiao, W.L. *et al.* (2006) ent-Labdane diterpenoids from Andrographis paniculata. *J Nat Prod*, **69**, 319-322.
1166. Shi, Q., Chen, K., Fujioka, T. *et al.* (1992) Antitumor agents, 135. Structure and stereochemistry of polacandrin, a new cytotoxic triterpene from Polanisia dodecandra. *J Nat Prod*, **55**, 1488-1497.
1167. Shi, T.X., Wang, S., Zeng, K.W. *et al.* (2013) Inhibitory constituents from the aerial parts of Polygala tenuifolia on LPS-induced NO production in BV2 microglia cells. *Bioorg Med Chem Lett*, **23**, 5904-5908.
1168. Shi, Y.Q., Fukai, T., Sakagami, H. *et al.* (2001) Cytotoxic flavonoids with isoprenoid groups from Morus mongolica. *J Nat Prod*, **64**, 181-188.
1169. Shi, Y.S., Liu, Y.B., Ma, S.G. *et al.* (2015) Bioactive Sesquiterpenes and Lignans from the Fruits of Xanthium sibiricum. *J Nat Prod*, **78**, 1526-1535.
1170. Shigemori, H. and Kobayashi, J. (2004) Biological activity and chemistry of taxoids from the Japanese yew, Taxus cuspidata. *J Nat Prod*, **67**, 245-256.
1171. Shim, J.S., Kim, J.H., Lee, J. *et al.* (2004) Anti-angiogenic activity of a homoisoflavanone from Cremastra appendiculata. *Planta Med*, **70**, 171-173.
1172. Shim, S.H., Kim, J.S. and Kang, S.S. (2003) Norditerpenoid alkaloids from the processed tubers of Aconitum carmichaeli. *Chem Pharm Bull (Tokyo)*, **51**, 999-1002.
1173. Shinozaki, Y., Fukamiya, N., Uchiyama, C. *et al.* (2002) Multidrug resistant cancer cells susceptibility to cytotoxic taxane diterpenes from Taxus yunnanensis and Taxus chinensis. *Bioorg Med Chem Lett*, **12**, 2785-2788.
1174. Shou, Q.Y., Tan, Q. and Shen, Z.W. (2009) Hirtellanines A and B, a pair of isomeric isoflavonoid derivatives from Campylotropis hirtella and their immunosuppressive activities. *Bioorg Med Chem Lett*, **19**, 3389-3391.
1175. Shrestha, S.P., Amano, Y., Narukawa, Y. *et al.* (2008) Nitric oxide production inhibitory activity of flavonoids contained in trunk exudates of Dalbergia sissoo. *J Nat Prod*, **71**, 98-101.
1176. Shu, X.K., Li, J., Liu, F. *et al.* (2013) Accelerated solvent extraction and pH-zone-refining counter-current chromatographic purification of yunaconitine and 8-deacetylyunaconitine from Aconitum vilmorinianum Kom. *J Sep Sci*, **36**, 2680-2685.
1177. Siddiqui, B.S., Afshan, F., Faizi, S. *et al.* (2002) Two new triterpenoids from Azadirachta indica and their insecticidal activity. *J Nat Prod*, **65**, 1216-1218.

1178. Siddiqui, S., Faizi, S., Siddiqui, B.S. *et al.* (1992) Constituents of Azadirachta indica: isolation and structure elucidation of a new antibacterial tetranortriterpenoid, mahmoodin, and a new protolimonoid, naheedin. *J Nat Prod*, **55**, 303-310.
1179. Silva, D.H., Davino, S.C., Barros, S.B. *et al.* (1999) Dihydrochalcones and flavonolignans from Iryanthera lancifolia. *J Nat Prod*, **62**, 1475-1478.
1180. Simmler, C., Antheaume, C., Andre, P. *et al.* (2011) Glucosyloxybenzyl eucomate derivatives from Vanda teres stimulate HaCaT cytochrome c oxidase. *J Nat Prod*, **74**, 949-955.
1181. Sinha, A., Taylor, W.H., Khan, I.H. *et al.* (1999) Glycoside primers of Psittacanthus cucullaris. *J Nat Prod*, **62**, 1036-1038.
1182. Sivasothy, Y., Hadi, A.H., Mohamad, K. *et al.* (2012) Spectaflavoside A, a new potent iron chelating dimeric flavonol glycoside from the rhizomes of Zingiber spectabile Griff. *Bioorg Med Chem Lett*, **22**, 3831-3836.
1183. Smejkal, K., Chudik, S., Kloucek, P. *et al.* (2008) Antibacterial C-geranylflavonoids from Paulownia tomentosa Fruits. *J Nat Prod*, **71**, 706-709.
1184. Smejkal, K., Grycova, L., Marek, R. *et al.* (2007) C-geranyl compounds from Paulownia tomentosa fruits. *J Nat Prod*, **70**, 1244-1248.
1185. Smejkal, K., Svacinova, J., Slapetova, T. *et al.* (2010) Cytotoxic activities of several geranyl-substituted flavanones. *J Nat Prod*, **73**, 568-572.
1186. Sneden, A.T. (1981) Isoiguesterin, a new antileukemic bisnortriterpene from Salacia madagascariensis. *J Nat Prod*, **44**, 503-507.
1187. Sohn, J.H., Han, K.L., Lee, S.H. *et al.* (2005) Protective effects of panduratin A against oxidative damage of tert-butylhydroperoxide in human HepG2 cells. *Biol Pharm Bull*, **28**, 1083-1086.
1188. Son, K.H., Kwon, S.J., Chang, H.W. *et al.* (2001) Papyriflavonol A, a new prenylated flavonol from Broussonetia papyrifera. *Fitoterapia*, **72**, 456-458.
1189. Song, C., Ring, L., Hoffmann, T. *et al.* (2015) Acylphloroglucinol Biosynthesis in Strawberry Fruit. *Plant Physiol*, **169**, 1656-1670.
1190. Song, S., Li, Y., Feng, Z. *et al.* (2010) Hepatoprotective constituents from the roots and stems of Erycibe hainanesis. *J Nat Prod*, **73**, 177-184.
1191. Song, W., Si, L., Ji, S. *et al.* (2014) Uralsaponins M-Y, antiviral triterpenoid saponins from the roots of Glycyrrhiza uralensis. *J Nat Prod*, **77**, 1632-1643.
1192. Song, W.H., Cheng, Z.H. and Chen, D.F. (2014) Anticomplement monoterpenoid glucosides from the root bark of Paeonia suffruticosa. *J Nat Prod*, **77**, 42-48.
1193. Songsiang, U., Thongthoom, T., Boonyarat, C. *et al.* (2011) Claurailas A-D, cytotoxic carbazole alkaloids from the roots of Clausena harmandiana. *J Nat Prod*, **74**, 208-212.
1194. Soriano-Agaton, F., Lagoutte, D., Poupon, E. *et al.* (2005) Extraction, hemisynthesis, and synthesis of canthin-6-one analogues. Evaluation of their antifungal activities. *J Nat Prod*, **68**, 1581-1587.
1195. Sreelatha, T., Hymavathi, A., Rama Subba Rao, V. *et al.* (2010) A new benzil derivative from Derris scandens: Structure-insecticidal activity study. *Bioorg Med Chem Lett*, **20**, 549-553.
1196. Sriyatep, T., Siridechakorn, I., Maneerat, W. *et al.* (2015) Bioactive prenylated xanthones from the young fruits and flowers of Garcinia cowa. *J Nat Prod*, **78**, 265-271.
1197. Staerk, D., Lykkeberg, A.K., Christensen, J. *et al.* (2002) In vitro cytotoxic activity of phenanthroindolizidine alkaloids from Cynanchum vincetoxicum and Tylophora tanakae against drug-sensitive and multidrug-resistant cancer cells. *J Nat Prod*, **65**, 1299-1302.
1198. Stavri, M., Paton, A., Skelton, B.W. *et al.* (2009) Antibacterial diterpenes from Plectranthus ernstii. *J Nat Prod*, **72**, 1191-1194.
1199. Steenkamp, P.A., Harding, N.M., van Heerden, F.R. *et al.* (2004) Fatal Datura poisoning: identification of atropine and scopolamine by high performance liquid chromatography/photodiode array/mass spectrometry. *Forensic Sci Int*, **145**, 31-39.
1200. Still, P.C., Yi, B., Gonzalez-Cestari, T.F. *et al.* (2013) Alkaloids from Microcos paniculata with cytotoxic and nicotinic receptor antagonistic activities. *J Nat Prod*, **76**, 243-249.
1201. Strehmel, N., Bottcher, C., Schmidt, S. *et al.* (2014) Profiling of secondary metabolites in root exudates of Arabidopsis thaliana. *Phytochemistry*, **108**, 35-46.
1202. Su, B.N., Cuendet, M., Hawthorne, M.E. *et al.* (2002) Constituents of the bark and twigs of Artocarpus dadah with cyclooxygenase inhibitory activity. *J Nat Prod*, **65**, 163-169.
1203. Su, B.N., Pawlus, A.D., Jung, H.A. *et al.* (2005) Chemical constituents of the fruits of Morinda citrifolia (Noni) and their antioxidant activity. *J Nat Prod*, **68**, 592-595.
1204. Su, Y.C., Hsu, K.P., Wang, E.I. *et al.* (2013) The composition, anti-mildew and anti-wood-decay fungal activities of the leaf and fruit oils of Juniperus formosana from Taiwan. *Nat Prod Commun*, **8**, 1329-1332.

1205. Suarez-Ortiz, G.A., Cerda-Garcia-Rojas, C.M., Hernandez-Rojas, A. *et al.* (2013) Absolute configuration and conformational analysis of brevipolides, bioactive 5,6-dihydro-alpha-pyrones from Hyptis brevipes. *J Nat Prod*, **76**, 72-78.
1206. Subeki, Matsuura, H., Takahashi, K. *et al.* (2007) Screening of Indonesian medicinal plant extracts for antibabesial activity and isolation of new quassinoids from Brucea javanica. *J Nat Prod*, **70**, 1654-1657.
1207. Sukpondma, Y., Rukachaisirikul, V. and Phongpaichit, S. (2005) Xanthone and sesquiterpene derivatives from the fruits of Garcinia scortechinii. *J Nat Prod*, **68**, 1010-1017.
1208. Sumarah, M.W., Puniani, E., Blackwell, B.A. *et al.* (2008) Characterization of polyketide metabolites from foliar endophytes of Picea glauca. *J Nat Prod*, **71**, 1393-1398.
1209. Sun, B., Morikawa, T., Matsuda, H. *et al.* (2004) Structures of new beta-carboline-type alkaloids with antiallergic effects from Stellaria dichotoma(1,2). *J Nat Prod*, **67**, 1464-1469.
1210. Sun, D.A., Starck, S.R., Locke, E.P. *et al.* (1999) DNA polymerase beta inhibitors from Sandoricum koetjape. *J Nat Prod*, **62**, 1110-1113.
1211. Sun, H.D., Qiu, S.X., Lin, L.Z. *et al.* (1996) Nigranoic acid, a triterpenoid from Schisandra sphaerandra that inhibits HIV-1 reverse transcriptase. *J Nat Prod*, **59**, 525-527.
1212. Sun, X.Q., Zhang, M.X., Yu, J.Y. *et al.* (2013) Glutathione S-transferase of brown planthoppers (Nilaparvata lugens) is essential for their adaptation to gramine-containing host plants. *PLoS One*, **8**, e64026.
1213. Sun, Y., Tabata, K., Matsubara, H. *et al.* (2008) New cytotoxic diarylheptanoids from the rhizomes of Alpinia officinarum. *Planta Med*, **74**, 427-431.
1214. Sun, Y., Yu, Z., Duan, W. *et al.* (2011) Isolation and purification of seven lignans from Magnolia sprengeri by high-speed counter-current chromatography. *J Chromatogr B Analyt Technol Biomed Life Sci*, **879**, 3775-3779.
1215. Sun, Y.J., Li, Z.L., Chen, H. *et al.* (2011) Three new cytotoxic aryltetralin lignans from Sinopodophyllum emodi. *Bioorg Med Chem Lett*, **21**, 3794-3797.
1216. Sun, Z.H., Chen, Y., Guo, Y.Q. *et al.* (2015) Isolation and cytotoxicity evaluation of taxanes from the barks of Taxus wallichiana var. mairei. *Bioorg Med Chem Lett*, **25**, 1240-1243.
1217. Sung, S.H. and Lee, M. (2015) Anti-adipogenic activity of a new cyclic diarylheptanoid isolated from Alnus japonica on 3T3-L1 cells via modulation of PPARgamma, C/EBPalpha and SREBP1c signaling. *Bioorg Med Chem Lett*, **25**, 4648-4651.
1218. Sureram, S., Senadeera, S.P., Hongmanee, P. *et al.* (2012) Antimycobacterial activity of bisbenzylisoquinoline alkaloids from Tiliacora triandra against multidrug-resistant isolates of Mycobacterium tuberculosis. *Bioorg Med Chem Lett*, **22**, 2902-2905.
1219. Suresh, G., Poornima, B., Babu, K.S. *et al.* (2013) Cytotoxic sesquiterpenes from Hedychium spicatum: isolation, structure elucidation and structure-activity relationship studies. *Fitoterapia*, **86**, 100-107.
1220. Suresh, G., Reddy, P.P., Babu, K.S. *et al.* (2010) Two new cytotoxic labdane diterpenes from the rhizomes of Hedychium coronarium. *Bioorg Med Chem Lett*, **20**, 7544-7548.
1221. Sutthivaiyakit, S., Thongnak, O., Lhinhatrakool, T. *et al.* (2009) Cytotoxic and antimycobacterial prenylated flavonoids from the roots of Eriosema chinense. *J Nat Prod*, **72**, 1092-1096.
1222. Suzuki, H., Tanabe, H., Mizukami, H. *et al.* (2011) Differential gene expression in rat vascular smooth muscle cells following treatment with coptisine exerts a selective antiproliferative effect. *J Nat Prod*, **74**, 634-638.
1223. Szokol-Borsodi, L., Solyomvary, A., Molnar-Perl, I. *et al.* (2012) Optimum yields of dibenzylbutyrolactone-type lignans from Cynareae fruits, during their ripening, germination and enzymatic hydrolysis processes, determined by on-line chromatographic methods. *Phytochem Anal*, **23**, 598-603.
1224. Tai, B.H., Nhiem, N.X., Quang, T.H. *et al.* (2011) A new iridoid and effect on the rat aortic vascular smooth muscle cell proliferation of isolated compounds from Buddleja officinalis. *Bioorg Med Chem Lett*, **21**, 3462-3466.
1225. Taira, J., Ohmine, W., Ogi, T. *et al.* (2012) Suppression of nitric oxide production on LPS/IFN-gamma-stimulated RAW264.7 macrophages by a novel catechin, pilosanol N, from Agrimonia pilosa Ledeb. *Bioorg Med Chem Lett*, **22**, 1766-1769.
1226. Takahashi, K., Kawaguchi, S., Nishimura, K. *et al.* (1974) Studies on constituents of medicinal plants. XIII. Constituents of the pericarps of the capsules of Euscaphis japonica Pax. *Chem Pharm Bull (Tokyo)*, **22**, 650-653.
1227. Takasaki, M., Tokuda, H., Nishino, H. *et al.* (1999) Cancer chemopreventive agents (antitumor-promoters) from Ajuga decumbens. *J Nat Prod*, **62**, 972-975.
1228. Takasaki, M., Yamauchi, I.I., Haruna, M. *et al.* (1998) New glycosides from ajuga decumbens. *J Nat Prod*, **61**, 1105-1109.

1229. Takashima, J., Chiba, N., Yoneda, K. *et al.* (2002) Derrisin, a new rotenoid from Derris malaccensis plain and anti-Helicobacter pylori activity of its related constituents. *J Nat Prod*, **65**, 611-613.
1230. Takemura, M., Endo, S., Matsunaga, T. *et al.* (2011) Selective inhibition of the tumor marker aldo-keto reductase family member 1B10 by oleanolic acid. *J Nat Prod*, **74**, 1201-1206.
1231. Tamura, S., Kubata, B.K., Syamsurizal *et al.* (2010) New anti-malarial phenylpropanoid conjugated iridoids from Morinda morindoides. *Bioorg Med Chem Lett*, **20**, 1520-1523.
1232. Tan, S.J., Low, Y.Y., Choo, Y.M. *et al.* (2010) Strychnan and secoangustilobine A type alkaloids from Alstonia spatulata. Revision of the C-20 configuration of scholaricine. *J Nat Prod*, **73**, 1891-1897.
1233. Tanaka, A., Arai, Y., Kim, S.N. *et al.* (2011) Synthesis and biological evaluation of bilobol and adipostatin A. *J Asian Nat Prod Res*, **13**, 290-296.
1234. Tanaka, N., Takaishi, Y., Shikishima, Y. *et al.* (2004) Prenylated benzophenones and xanthones from Hypericum scabrum. *J Nat Prod*, **67**, 1870-1875.
1235. Tang, C.P., Chen, T., Velten, R. *et al.* (2008) Alkaloids from stems and leaves of Stemona japonica and their insecticidal activities. *J Nat Prod*, **71**, 112-116.
1236. Tang, M., Shen, D., Hu, Y. *et al.* (2004) Cytotoxic triterpenoid saponins from Symplocos chinensis. *J Nat Prod*, **67**, 1969-1974.
1237. Tang, Q., Shao, M., Wang, Y. *et al.* (2015) Simultaneous Determination of 10 Bioactive Components of Lophatherum gracile Brongn by HPLC-DAD. *J Chromatogr Sci*, **53**, 963-967.
1238. Tang, S.Y., Whiteman, M., Peng, Z.F. *et al.* (2004) Characterization of antioxidant and antiglycation properties and isolation of active ingredients from traditional chinese medicines. *Free Radic Biol Med*, **36**, 1575-1587.
1239. Tang, W., Hioki, H., Harada, K. *et al.* (2007) Antioxidant phenylpropanoid-substituted epicatechins from Trichilia catigua. *J Nat Prod*, **70**, 2010-2013.
1240. Tao, Q.F., Xu, Y., Lam, R.Y. *et al.* (2008) Diarylheptanoids and a monoterpenoid from the rhizomes of Zingiber officinale: antioxidant and cytoprotective properties. *J Nat Prod*, **71**, 12-17.
1241. Tao, S.J., Guan, S.H., Wang, W. *et al.* (2009) Cytotoxic polyprenylated xanthones from the resin of Garcinia hanburyi. *J Nat Prod*, **72**, 117-124.
1242. Tapondjou, L.A., Lontsi, D., Sondengam, B.L. *et al.* (2003) Saponins from Cussonia bancoensis and their inhibitory effects on nitric oxide production. *J Nat Prod*, **66**, 1266-1269.
1243. Tapondjou, L.A., Ponou, K.B., Teponno, R.B. *et al.* (2008) In vivo anti-inflammatory effect of a new steroidal saponin, mannioside A, and its derivatives isolated from Dracaena mannii. *Arch Pharm Res*, **31**, 653-658.
1244. Tezuka, Y., Morikawa, K., Li, F. *et al.* (2011) Cytochrome P450 3A4 inhibitory constituents of the wood of Taxus yunnanensis. *J Nat Prod*, **74**, 102-105.
1245. Thanh, V.T., Pham, V.C., Mai, H.D. *et al.* (2012) Cytotoxic lignans from fruits of Cleistanthus indochinensis: synthesis of cleistantoxin derivatives. *J Nat Prod*, **75**, 1578-1583.
1246. Thao, N.P., Luyen, B.T., Tai, B.H. *et al.* (2015) Chemical constituents of Miliusa balansae leaves and inhibition of nitric oxide production in lipopolysaccharide-induced RAW 264.7 cells. *Bioorg Med Chem Lett*, **25**, 3859-3863.
1247. Thao, N.P., Luyen, B.T., Tai, B.H. *et al.* (2014) Rat intestinal sucrase inhibition of constituents from the roots of Rosa rugosa Thunb. *Bioorg Med Chem Lett*, **24**, 1192-1196.
1248. Theodori, R., Karioti, A., Rancic, A. *et al.* (2006) Linear sesquiterpene lactones from Anthemis auriculata and their antibacterial activity. *J Nat Prod*, **69**, 662-664.
1249. Thiem, D.A., Sneden, A.T., Khan, S.I. *et al.* (2005) Bisnortriterpenes from Salacia madagascariensis. *J Nat Prod*, **68**, 251-254.
1250. Thomas, S., Senthilkumar, G.P., Sivaraman, K. *et al.* (2015) Effect of s-methyl-L-cysteine on oxidative stress, inflammation and insulin resistance in male wistar rats fed with high fructose diet. *Iran J Med Sci*, **40**, 45-50.
1251. Thongnest, S., Mahidol, C., Sutthivaiyakit, S. *et al.* (2005) Oxygenated pimarane diterpenes from Kaempferia marginata. *J Nat Prod*, **68**, 1632-1636.
1252. Thuong, P.T., Kang, K.W., Kim, J.K. *et al.* (2009) Lithospermic acid derivatives from Lithospermum erythrorhizon increased expression of serine palmitoyltransferase in human HaCaT cells. *Bioorg Med Chem Lett*, **19**, 1815-1817.
1253. Tian, F., Chang, C.J., Grutzner, J.B. *et al.* (2001) Robinlin: a novel bioactive homo-monoterpene from Robinia pseudoacacia L. (Fabaceae). *Bioorg Med Chem Lett*, **11**, 2603-2606.
1254. Tian, F. and McLaughlin, J.L. (2000) Bioactive flavonoids from the black locust tree, robinia pseudoacacia. *Pharm Biol*, **38**, 229-234.
1255. Tian, J., Shen, Y., Yang, X. *et al.* (2010) Antifungal cyclic peptides from Psammosilene tunicoides. *J Nat Prod*, **73**, 1987-1992.

1256. Tian, L.W., Pei, Y., Zhang, Y.J. *et al.* (2009) 7-O-methylkaempferol and -quercetin glycosides from the whole plant of Nervilia fordii. *J Nat Prod*, **72**, 1057-1060.
1257. Tian, L.W., Zhang, Y.J., Qu, C. *et al.* (2010) Phloroglucinol glycosides from the fresh fruits of Eucalyptus maideni. *J Nat Prod*, **73**, 160-163.
1258. Tian, L.W., Zhang, Y.J., Wang, Y.F. *et al.* (2009) Eucalmaidins A-E, (+)-oleuropeic acid derivatives from the fresh leaves of Eucalyptus maideni. *J Nat Prod*, **72**, 1608-1611.
1259. Tian, Q., Li, J., Xie, X. *et al.* (2005) Stereospecific induction of nuclear factor-kappaB activation by isochamaejasmin. *Mol Pharmacol*, **68**, 1534-1542.
1260. Tian, Y., Xu, W., Zhu, C. *et al.* (2013) Diterpenoids with diverse skeletons from the roots of Euphorbia micractina. *J Nat Prod*, **76**, 1039-1046.
1261. Tian, Y., Xu, W., Zhu, C. *et al.* (2011) Lathyrane diterpenoids from the roots of Euphorbia micractina and their biological activities. *J Nat Prod*, **74**, 1221-1229.
1262. Tokar, M. and Klimek, B. (2004) Isolation and identification of biologically active compounds from Forsythia viridissima flowers. *Acta Pol Pharm*, **61**, 191-197.
1263. Tong, X.G., Wu, G.S., Huang, C.G. *et al.* (2010) Compounds from Acorus tatarinowii: determination of absolute configuration by quantum computations and cAMP regulation activity. *J Nat Prod*, **73**, 1160-1163.
1264. Tong, Y., Zhou, X.M., Wang, S.J. *et al.* (2009) Analgesic activity of myricetin isolated from Myrica rubra Sieb. et Zucc. leaves. *Arch Pharm Res*, **32**, 527-533.
1265. Toriizuka, Y., Kinoshita, E., Kogure, N. *et al.* (2008) New lycorine-type alkaloid from Lycoris traubii and evaluation of antitrypanosomal and antimalarial activities of lycorine derivatives. *Bioorg Med Chem*, **16**, 10182-10189.
1266. Torres-Romero, D., Jimenez, I.A., Rojas, R. *et al.* (2011) Dihydro-beta-agarofuran sesquiterpenes isolated from Celastrus vulcanicola as potential anti-Mycobacterium tuberculosis multidrug-resistant agents. *Bioorg Med Chem*, **19**, 2182-2189.
1267. Torres-Romero, D., King-Diaz, B., Jimenez, I.A. *et al.* (2008) Sesquiterpenes from Celastrus vulcanicola as photosynthetic inhibitors. *J Nat Prod*, **71**, 1331-1335.
1268. Torres-Romero, D., King-Diaz, B., Strasser, R.J. *et al.* (2010) Friedelane triterpenes from Celastrus vulcanicola as photosynthetic inhibitors. *J Agric Food Chem*, **58**, 10847-10854.
1269. Toume, K., Habu, T., Arai, M.A. *et al.* (2015) Prenylated flavonoids and resveratrol derivatives isolated from Artocarpus communis with the ability to overcome TRAIL resistance. *J Nat Prod*, **78**, 103-110.
1270. Toume, K., Nakazawa, T., Ohtsuki, T. *et al.* (2011) Cycloartane triterpenes isolated from Combretum quadrangulare in a screening program for death-receptor expression enhancing activity. *J Nat Prod*, **74**, 249-255.
1271. Tran, T.D., Pham, N.B., Fechner, G. *et al.* (2010) Chemical investigation of drug-like compounds from the Australian tree, Neolitseadealbata. *Bioorg Med Chem Lett*, **20**, 5859-5863.
1272. Tran, T.L., Kim, Y.R., Yang, J.L. *et al.* (2014) Dammarane triterpenes from the leaves of Panax ginseng enhance cellular immunity. *Bioorg Med Chem*, **22**, 499-504.
1273. Truong, N.B., Pham, C.V., Doan, H.T. *et al.* (2011) Antituberculosis cycloartane triterpenoids from Radermachera boniana. *J Nat Prod*, **74**, 1318-1322.
1274. Tsai, S.F. and Lee, S.S. (2010) Characterization of Acetylcholinesterase Inhibitory Constituents from Annona glabra Assisted by HPLC Microfractionation. *J Nat Prod*, **73**, 1632-1635.
1275. Tsai, W.J., Shen, C.C., Tsai, T.H. *et al.* (2014) Lignans from the aerial parts of Saururus chinensis: isolation, structural characterization, and their effects on platelet aggregation. *J Nat Prod*, **77**, 125-131.
1276. Tselepi, M., Papachristou, E., Emmanouilidi, A. *et al.* (2011) Catalytic inhibition of eukaryotic topoisomerases I and II by flavonol glycosides extracted from Vicia faba and Lotus edulis. *J Nat Prod*, **74**, 2362-2370.
1277. Tseng, H.C., Wu, W.T., Huang, H.S. *et al.* (2014) Antimicrobial activities of various fractions of longan (Dimocarpus longan Lour. Fen Ke) seed extract. *Int J Food Sci Nutr*, **65**, 589-593.
1278. Tseng, M.H., Chou, C.H., Chen, Y.M. *et al.* (2001) Allelopathic prenylflavanones from the fallen leaves of Macaranga tanarius. *J Nat Prod*, **64**, 827-828.
1279. Tsevegsuren, N., Edrada, R., Lin, W. *et al.* (2007) Biologically active natural products from Mongolian medicinal plants Scorzonera divaricata and Scorzonera pseudodivaricata. *J Nat Prod*, **70**, 962-967.
1280. Tsuchiya, H. and Iinuma, M. (2000) Reduction of membrane fluidity by antibacterial sophoraflavanone G isolated from Sophora exigua. *Phytomedicine*, **7**, 161-165.
1281. Tsurumaru, Y., Sasaki, K., Miyawaki, T. *et al.* (2012) HlPT-1, a membrane-bound prenyltransferase responsible for the biosynthesis of bitter acids in hops. *Biochem Biophys Res Commun*, **417**, 393-398.
1282. Tumaney, A.W., Shekar, S. and Rajasekharan, R. (2001) Identification, purification, and characterization of monoacylglycerol acyltransferase from developing peanut cotyledons. *J Biol Chem*, **276**, 10847-10852.

1283. Tung, N.H., Kwon, H.J., Kim, J.H. *et al.* (2010) Anti-influenza diarylheptanoids from the bark of Alnus japonica. *Bioorg Med Chem Lett*, **20**, 1000-1003.
1284. Tung, N.H., Uto, T., Sakamoto, A. *et al.* (2013) Antiproliferative and apoptotic effects of compounds from the flower of Mammea siamensis (Miq.) T. Anders. on human cancer cell lines. *Bioorg Med Chem Lett*, **23**, 158-162.
1285. Tung, Y.T. and Chang, S.T. (2010) Inhibition of xanthine oxidase by Acacia confusa extracts and their phytochemicals. *J Agric Food Chem*, **58**, 781-786.
1286. Uchiyama, N., Matsunaga, K., Kiuchi, F. *et al.* (2002) Trypanocidal terpenoids from Laurus nobilis L. *Chem Pharm Bull (Tokyo)*, **50**, 1514-1516.
1287. Uddin, S.J., Jason, T.L., Beattie, K.D. *et al.* (2011) (2S,3S)-sulfated pterosin C, a cytotoxic sesquiterpene from the Bangladeshi mangrove fern Acrostichum aureum. *J Nat Prod*, **74**, 2010-2013.
1288. Uddin, S.J., Nahar, L., Shilpi, J.A. *et al.* (2007) Gedunin, a limonoid from Xylocarpus granatum, inhibits the growth of CaCo-2 colon cancer cell line in vitro. *Phytother Res*, **21**, 757-761.
1289. Ueda, J.Y., Takagi, M. and Shin-ya, K. (2009) Aminocaprophenone- and pyrrolidine-type alkaloids from the leaves of Ficus septica. *J Nat Prod*, **72**, 2181-2183.
1290. Ueda, J.Y., Tezuka, Y., Banskota, A.H. *et al.* (2003) Constituents of the Vietnamese medicinal plant Streptocaulon juventas and their antiproliferative activity against the human HT-1080 fibrosarcoma cell line. *J Nat Prod*, **66**, 1427-1433.
1291. Uehara, A., Kitajima, J., Kokubugata, G. *et al.* (2014) Further characterization of foliar flavonoids in Crossostephium chinense and their geographic variation. *Nat Prod Commun*, **9**, 163-164.
1292. Ukiya, M., Akihisa, T., Yasukawa, K. *et al.* (2007) Triterpene glycosides from the flower petals of sunflower (Helianthus annuus) and their anti-inflammatory activity. *J Nat Prod*, **70**, 813-816.
1293. Ukiya, M., Akihisa, T., Yasukawa, K. *et al.* (2006) Anti-inflammatory, anti-tumor-promoting, and cytotoxic activities of constituents of marigold (Calendula officinalis) flowers. *J Nat Prod*, **69**, 1692-1696.
1294. Umehara, K., Nemoto, K., Matsushita, A. *et al.* (2009) Flavonoids from the heartwood of the Thai medicinal plant Dalbergia parviflora and their effects on estrogenic-responsive human breast cancer cells. *J Nat Prod*, **72**, 2163-2168.
1295. Usia, T., Iwata, H., Hiratsuka, A. *et al.* (2004) Sesquiterpenes and flavonol glycosides from Zingiber aromaticum and their CYP3A4 and CYP2D6 inhibitory activities. *J Nat Prod*, **67**, 1079-1083.
1296. Usia, T., Watabe, T., Kadota, S. *et al.* (2005) Potent CYP3A4 inhibitory constituents of Piper cubeba. *J Nat Prod*, **68**, 64-68.
1297. Uvarani, C., Sankaran, M., Jaivel, N. *et al.* (2013) Bioactive dimeric carbazole alkaloids from Murraya koenigii. *J Nat Prod*, **76**, 993-1000.
1298. Uwai, K., Ohashi, K., Takaya, Y. *et al.* (2000) Exploring the structural basis of neurotoxicity in C(17)-polyacetylenes isolated from water hemlock. *J Med Chem*, **43**, 4508-4515.
1299. Valencia-Islas, N., Abbas, H., Bye, R. *et al.* (2002) Phytotoxic compounds from Prionosciadium watsoni. *J Nat Prod*, **65**, 828-834.
1300. Van Quaquebeke, E., Simon, G., Andre, A. *et al.* (2005) Identification of a novel cardenolide (2''-oxovoruscharin) from Calotropis procera and the hemisynthesis of novel derivatives displaying potent in vitro antitumor activities and high in vivo tolerance: structure-activity relationship analyses. *J Med Chem*, **48**, 849-856.
1301. Veras, M.L., Bezerra, M.Z., Lemos, T.L. *et al.* (2004) Cytotoxic withaphysalins from the leaves of Acnistus arborescens. *J Nat Prod*, **67**, 710-713.
1302. Verhoff, M., Seitz, S., Paul, M. *et al.* (2014) Tetra- and pentacyclic triterpene acids from the ancient anti-inflammatory remedy frankincense as inhibitors of microsomal prostaglandin E(2) synthase-1. *J Nat Prod*, **77**, 1445-1451.
1303. Vermeersch, K.A., Wang, L., McDonald, J.F. *et al.* (2014) Distinct metabolic responses of an ovarian cancer stem cell line. *BMC Syst Biol*, **8**, 134.
1304. Vermillion, K., Holguin, F.O., Berhow, M.A. *et al.* (2011) Dinoxin B, a withanolide from Datura inoxia leaves with specific cytotoxic activities. *J Nat Prod*, **74**, 267-271.
1305. Versiani, M.A., Diyabalanage, T., Ratnayake, R. *et al.* (2011) Flavonoids from eight tropical plant species that inhibit the multidrug resistance transporter ABCG2. *J Nat Prod*, **74**, 262-266.
1306. Vianna, D.R., Hamerski, L., Figueiro, F. *et al.* (2012) Selective cytotoxicity and apoptosis induction in glioma cell lines by 5-oxygenated-6,7-methylenedioxycoumarins from Pterocaulon species. *Eur J Med Chem*, **57**, 268-274.
1307. Villarini, M., Pagiotti, R., Dominici, L. *et al.* (2014) Investigation of the cytotoxic, genotoxic, and apoptosis-inducing effects of estragole isolated from fennel (Foeniculum vulgare). *J Nat Prod*, **77**, 773-778.
1308. Vogel, S. and Heilmann, J. (2008) Synthesis, cytotoxicity, and antioxidative activity of minor prenylated chalcones from Humulus lupulus. *J Nat Prod*, **71**, 1237-1241.

1309. Wada, K., Hazawa, M., Takahashi, K. *et al.* (2007) Inhibitory effects of diterpenoid alkaloids on the growth of A172 human malignant cells. *J Nat Prod*, **70**, 1854-1858.
1310. Wada, S., Yasui, Y., Hitomi, T. *et al.* (2007) Structures and radical-scavenging activities of phenolic constituents from the bark of Picea jezoensis var. jezoensis. *J Nat Prod*, **70**, 1605-1610.
1311. Wada, S., Yasui, Y., Tokuda, H. *et al.* (2009) Anti-tumor-initiating effects of phenolic compounds isolated from the bark of Picea jezoensis var. jezoensis. *Bioorg Med Chem*, **17**, 6414-6421.
1312. Wall, M.E., Wani, M.C., Natschke, S.M. *et al.* (1986) Plant antitumor agents. 22. Isolation of 11-hydroxycamptothecin from Camptotheca acuminata Decne: total synthesis and biological activity. *J Med Chem*, **29**, 1553-1555.
1313. Wan, C., Yuan, T., Li, L. *et al.* (2012) Maplexins, new alpha-glucosidase inhibitors from red maple (Acer rubrum) stems. *Bioorg Med Chem Lett*, **22**, 597-600.
1314. Wang, B.G., Ebel, R., Nugroho, B.W. *et al.* (2001) Aglacins A-D, first representatives of a new class of aryltetralin cyclic ether lignans from Aglaia cordata. *J Nat Prod*, **64**, 1521-1526.
1315. Wang, C., Li, C.J., Yang, J.Z. *et al.* (2013) Anti-inflammatory sesquiterpene derivatives from the leaves of Tripterygium wilfordii. *J Nat Prod*, **76**, 85-90.
1316. Wang, D., Girard, T.J., Kasten, T.P. *et al.* (1998) Inhibitory activity of unsaturated fatty acids and anacardic acids toward soluble tissue factor-factor VIIa complex. *J Nat Prod*, **61**, 1352-1355.
1317. Wang, F., Hua, H., Pei, Y. *et al.* (2006) Triterpenoids from the resin of Styrax tonkinensis and their antiproliferative and differentiation effects in human leukemia HL-60 cells. *J Nat Prod*, **69**, 807-810.
1318. Wang, H.M., Chen, C.Y., Chen, C.Y. *et al.* (2010) (-)-N-Formylanonaine from Michelia alba as a human tyrosinase inhibitor and antioxidant. *Bioorg Med Chem*, **18**, 5241-5247.
1319. Wang, J., Dong, S., Wang, Y. *et al.* (2008) Cyclic diarylheptanoids from Myrica nana inhibiting nitric oxide release. *Bioorg Med Chem*, **16**, 8510-8515.
1320. Wang, J., Zhao, Y.M., Zhang, M.L. *et al.* (2015) Simultaneous determination of chlorogenic acid, caffeic acid, alantolactone and isoalantolactone in Inula helenium by HPLC. *J Chromatogr Sci*, **53**, 526-530.
1321. Wang, J.F., He, W.J., Zhang, X.X. *et al.* (2015) Dicarabrol, a new dimeric sesquiterpene from Carpesium abrotanoides L. *Bioorg Med Chem Lett*, **25**, 4082-4084.
1322. Wang, J.F., Yang, S.H., Liu, Y.Q. *et al.* (2015) Five new phorbol esters with cytotoxic and selective anti-inflammatory activities from Croton tiglium. *Bioorg Med Chem Lett*, **25**, 1986-1989.
1323. Wang, J.N., Hou, C.Y., Liu, Y.L. *et al.* (1994) Swertifrancheside, an HIV-reverse transcriptase inhibitor and the first flavone-xanthone dimer, from Swertia franchetiana. *J Nat Prod*, **57**, 211-217.
1324. Wang, J.Z., Chen, Q.H. and Wang, F.P. (2010) Cytotoxic bisbenzylisoquinoline alkaloids from the roots of Cyclea racemosa. *J Nat Prod*, **73**, 1288-1293.
1325. Wang, K., Zhou, X.Y., Wang, Y.Y. *et al.* (2011) Macrophyllionium and macrophyllines A and B, oxindole alkaloids from Uncaria macrophylla. *J Nat Prod*, **74**, 12-15.
1326. Wang, L., Bai, L., Nagasawa, T. *et al.* (2008) Bioactive triterpene saponins from the roots of Phytolacca americana. *J Nat Prod*, **71**, 35-40.
1327. Wang, L.Y., Wang, N.L., Yao, X.S. *et al.* (2002) Diterpenes from the roots of Euphorbia kansui and their in vitro effects on the cell division of Xenopus. *J Nat Prod*, **65**, 1246-1251.
1328. Wang, M., Shao, Y., Li, J. *et al.* (1999) Antioxidative phenolic glycosides from sage (Salvia officinalis). *J Nat Prod*, **62**, 454-456.
1329. Wang, N., Li, Z., Song, D. *et al.* (2008) Lanostane-type triterpenoids from the roots of Kadsura coccinea. *J Nat Prod*, **71**, 990-994.
1330. Wang, Q., Chen, T.H., Bastow, K.F. *et al.* (2010) Altaicalarins A-D, cytotoxic bisabolane sesquiterpenes from Ligularia altaica. *J Nat Prod*, **73**, 139-142.
1331. Wang, Q., Chen, T.H., Bastow, K.F. *et al.* (2013) Songaricalarins A-E, cytotoxic oplopane sesquiterpenes from Ligularia songarica. *J Nat Prod*, **76**, 305-310.
1332. Wang, Q., Mu, Q., Shibano, M. *et al.* (2007) Eremophilane sesquiterpenes from Ligularia macrophylla. *J Nat Prod*, **70**, 1259-1262.
1333. Wang, Q.H., Kuang, H.X., Yang, B.Y. *et al.* (2011) Sesquiterpenes from Chloranthus japonicus. *J Nat Prod*, **74**, 16-20.
1334. Wang, S.Q., Han, X.Z., Li, X. *et al.* (2010) Flavonoids from Dracocephalum tanguticum and their cardioprotective effects against doxorubicin-induced toxicity in H9c2 cells. *Bioorg Med Chem Lett*, **20**, 6411-6415.
1335. Wang, S.S., Zhang, X.J., Que, S. *et al.* (2012) 3-Hydroxy-3-methylglutaryl flavonol glycosides from Oxytropis falcata. *J Nat Prod*, **75**, 1359-1364.
1336. Wang, T.M., Hojo, T., Ran, F.X. *et al.* (2007) Cardenolides from Saussurea stella with cytotoxicity toward cancer cells. *J Nat Prod*, **70**, 1429-1433.

1337. Wang, W., Chen, W., Yang, Y. *et al.* (2015) New phenolic compounds from Coreopsis tinctoria Nutt. and their antioxidant and angiotensin i-converting enzyme inhibitory activities. *J Agric Food Chem*, **63**, 200-207.
1338. Wang, W., Liu, J., Han, J. *et al.* (2006) New triterpenoids from Kadsura heteroclita and their cytotoxic activity. *Planta Med*, **72**, 450-457.
1339. Wang, W.G., Li, X.N., Du, X. *et al.* (2012) Laxiflorolides A and B, epimeric bishomoditerpene lactones from Isodon eriocalyx. *J Nat Prod*, **75**, 1102-1107.
1340. Wang, X., Zhen, L., Zhang, G. *et al.* (2011) Osteogenic effects of flavonoid aglycones from an osteoprotective fraction of Drynaria fortunei--an in vitro efficacy study. *Phytomedicine*, **18**, 868-872.
1341. Wang, Y., Curtis-Long, M.J., Lee, B.W. *et al.* (2014) Inhibition of tyrosinase activity by polyphenol compounds from Flemingia philippinensis roots. *Bioorg Med Chem*, **22**, 1115-1120.
1342. Wang, Y., Curtis-Long, M.J., Yuk, H.J. *et al.* (2013) Bacterial neuraminidase inhibitory effects of prenylated isoflavones from roots of Flemingia philippinensis. *Bioorg Med Chem*, **21**, 6398-6404.
1343. Wang, Y., Wang, W.J., Su, C. *et al.* (2013) Cytotoxic quassinoids from Ailanthus altissima. *Bioorg Med Chem Lett*, **23**, 654-657.
1344. Wang, Y.B., Huang, R., Wang, H.B. *et al.* (2006) Diterpenoids from the roots of Euphorbia fischeriana. *J Nat Prod*, **69**, 967-970.
1345. Wang, Y.G., Ren, J., Wang, A.G. *et al.* (2013) Hepatoprotective prenylaromadendrane-type diterpenes from the gum resin of Boswellia carterii. *J Nat Prod*, **76**, 2074-2079.
1346. Wang, Y.R., Xu, Y., Jiang, Z.Z. *et al.* (2015) Deoxypodophyllotoxin induces G2/M cell cycle arrest and apoptosis in SGC-7901 cells and inhibits tumor growth in vivo. *Molecules*, **20**, 1661-1675.
1347. Wang, Z., Wen, J., Xing, J. *et al.* (2006) Quantitative determination of diterpenoid alkaloids in four species of Aconitum by HPLC. *J Pharm Biomed Anal*, **40**, 1031-1034.
1348. Wansi, J.D., Mesaik, M.A., Chiozem, D.D. *et al.* (2008) Oxidative burst inhibitory and cytotoxic indoloquinazoline and furoquinoline alkaloids from Oricia suaveolens. *J Nat Prod*, **71**, 1942-1945.
1349. Wei, F., Ma, S.C., Ma, L.Y. *et al.* (2004) Antiviral flavonoids from the seeds of Aesculus chinensis. *J Nat Prod*, **67**, 650-653.
1350. Wei, X.N., Lin, B.B., Xie, G.Y. *et al.* (2013) [Chemical constituents of seeds of Oroxylum indicum]. *Zhongguo Zhong Yao Za Zhi*, **38**, 204-207.
1351. Wei, X.Y., Leung, C.Y., Wong, C.K. *et al.* (2005) Bisindigotin, a TCDD antagonist from the Chinese medicinal herb Isatis indigotica. *J Nat Prod*, **68**, 427-429.
1352. Weigenand, O., Hussein, A.A., Lall, N. *et al.* (2004) Antibacterial activity of naphthoquinones and triterpenoids from Euclea natalensis root bark. *J Nat Prod*, **67**, 1936-1938.
1353. Wen, C.W., Lin, X.D., Dong, M.J. *et al.* (2016) An Evaluation of 1-Deoxynojirimycin Oral Administration in Eri Silkworm through Fat Body Metabolomics Based on (1) H Nuclear Magnetic Resonance. *Biomed Res Int*, **2016**, 4676505.
1354. Wen, J., Shi, H., Xu, Z. *et al.* (2010) Dimeric guaianolides and sesquiterpenoids from Artemisia anomala. *J Nat Prod*, **73**, 67-70.
1355. Weng, J.R., Tsao, L.T., Yen, M.H. *et al.* (2003) Anti-inflammatory constituents and new pterocarpanoid of Crotalaria pallida. *J Nat Prod*, **66**, 404-407.
1356. Westenburg, H.E., Lee, K.J., Lee, S.K. *et al.* (2000) Activity-guided isolation of antioxidative constituents of Cotinus coggygria. *J Nat Prod*, **63**, 1696-1698.
1357. Whitson, E.L., Sun, H., Thomas, C.L. *et al.* (2012) Synergistic TRAIL sensitizers from Barleria alluaudii and Diospyros maritima. *J Nat Prod*, **75**, 394-399.
1358. Williams, R.B., Norris, A., Slebodnick, C. *et al.* (2005) Cytotoxic sesquiterpene lactones from Vernonia pachyclada from the Madagascar rainforest. *J Nat Prod*, **68**, 1371-1374.
1359. Win, N.N., Awale, S., Esumi, H. *et al.* (2007) Bioactive secondary metabolites from Boesenbergia pandurata of Myanmar and their preferential cytotoxicity against human pancreatic cancer PANC-1 cell line in nutrient-deprived medium. *J Nat Prod*, **70**, 1582-1587.
1360. Winkelmann, K., Heilmann, J., Zerbe, O. *et al.* (2000) New phloroglucinol derivatives from Hypericum papuanum. *J Nat Prod*, **63**, 104-108.
1361. Woldemichael, G.M., Gutierrez-Lugo, M.T., Franzblau, S.G. *et al.* (2004) Mycobacterium tuberculosis growth inhibition by constituents of Sapium haematospermum. *J Nat Prod*, **67**, 598-603.
1362. Wong, C.P., Deguchi, J., Nugroho, A.E. *et al.* (2013) Ceramicines from Chisocheton ceramicus as lipid-droplets accumulation inhibitors. *Bioorg Med Chem Lett*, **23**, 1786-1788.
1363. Wong, S.L., Chang, H.S., Wang, G.J. *et al.* (2011) Secondary metabolites from the roots of Neolitsea daibuensis and their anti-inflammatory activity. *J Nat Prod*, **74**, 2489-2496.
1364. Woo, H.S., Kim, D.W., Curtis-Long, M.J. *et al.* (2011) Potent inhibition of bacterial neuraminidase activity by pterocarpans isolated from the roots of Lespedeza bicolor. *Bioorg Med Chem Lett*, **21**, 6100-6103.

1365. Woo, K.W., Moon, E., Kwon, O.W. *et al.* (2013) Anti-neuroinflammatory diarylheptanoids from the rhizomes of Dioscorea nipponica. *Bioorg Med Chem Lett*, **23**, 3806-3809.
1366. Wu, B., Chen, J., Qu, H. *et al.* (2008) Complex sesquiterpenoids with tyrosinase inhibitory activity from the leaves of Chloranthus tianmushanensis. *J Nat Prod*, **71**, 877-880.
1367. Wu, C., Gunatilaka, A.A., McCabe, F.L. *et al.* (1997) Bioactive and other sesquiterpenes from Chiloscyphus rivularis. *J Nat Prod*, **60**, 1281-1286.
1368. Wu, C.C., Lu, Y.H., Wei, B.L. *et al.* (2008) Phloroglucinols with prooxidant activity from Garcinia subelliptica. *J Nat Prod*, **71**, 246-250.
1369. Wu, C.C., Weng, J.R., Won, S.J. *et al.* (2005) Constituents of the pericarp of Garcinia subelliptica. *J Nat Prod*, **68**, 1125-1127.
1370. Wu, H.Y., Zhan, R., Wang, W.G. *et al.* (2014) Cytotoxic ent-kaurane diterpenoids from Isodon wikstroemioides. *J Nat Prod*, **77**, 931-941.
1371. Wu, J., Yang, S.X., Li, M.Y. *et al.* (2010) Limonoids and tirucallane derivatives from the seeds of a krishna mangrove, Xylocarpus moluccensis. *J Nat Prod*, **73**, 644-649.
1372. Wu, J.M., Wang, Z.R., Hsieh, T.C. *et al.* (2001) Mechanism of cardioprotection by resveratrol, a phenolic antioxidant present in red wine (Review). *Int J Mol Med*, **8**, 3-17.
1373. Wu, M.C., Peng, C.F., Chen, I.S. *et al.* (2011) Antitubercular chromones and flavonoids from Pisonia aculeata. *J Nat Prod*, **74**, 976-982.
1374. Wu, P., Ma, G., Li, N. *et al.* (2015) Investigation of in vitro and in vivo antioxidant activities of flavonoids rich extract from the berries of Rhodomyrtus tomentosa(Ait.) Hassk. *Food Chem*, **173**, 194-202.
1375. Wu, Q.L., Wang, S.P., Du, L.J. *et al.* (1998) Xanthones from Hypericum japonicum and H. henryi. *Phytochemistry*, **49**, 1395-1402.
1376. Wu, S.F., Chang, F.R., Wang, S.Y. *et al.* (2011) Anti-inflammatory and cytotoxic neoflavonoids and benzofurans from Pterocarpus santalinus. *J Nat Prod*, **74**, 989-996.
1377. Wu, S.F., Hwang, T.L., Chen, S.L. *et al.* (2011) Bioactive components from the heartwood of Pterocarpus santalinus. *Bioorg Med Chem Lett*, **21**, 5630-5632.
1378. Wu, T., Wang, Q., Jiang, C. *et al.* (2015) Neo-clerodane diterpenoids from Scutellaria barbata with activity against Epstein-Barr virus lytic replication. *J Nat Prod*, **78**, 500-509.
1379. Wu, T.S., Chan, Y.Y., Leu, Y.L. *et al.* (1999) Sesquiterpene esters of aristolochic acid from the root and stem of Aristolochia heterophylla. *J Nat Prod*, **62**, 415-418.
1380. Wu, T.S., Lin, Y.M., Haruna, M. *et al.* (1991) Antitumor agents, 119. Kansuiphorins A and B, two novel antileukemic diterpene esters from Euphorbia kansui. *J Nat Prod*, **54**, 823-829.
1381. Wu, Y., Wu, Z.R., Chen, P. *et al.* (2015) Effect of the tyrosinase inhibitor (S)-N-trans-feruloyloctopamine from garlic skin on tyrosinase gene expression and melanine accumulation in melanoma cells. *Bioorg Med Chem Lett*, **25**, 1476-1478.
1382. Wu, Y.C., Hung, Y.C., Chang, F.R. *et al.* (1996) Identification of ent-16 beta, 17-dihydroxykauran-19-oic acid as an anti-HIV principle and isolation of the new diterpenoids annosquamosins A and B from Annona squamosa. *J Nat Prod*, **59**, 635-637.
1383. Xiao, G., Li, G., Chen, L. *et al.* (2010) Isolation of antioxidants from Psoralea corylifolia fruits using high-speed counter-current chromatography guided by thin layer chromatography-antioxidant autographic assay. *J Chromatogr A*, **1217**, 5470-5476.
1384. Xiao, W., Wang, Y., Zhang, P. *et al.* (2013) Bioactive barrigenol type triterpenoids from the leaves of Xanthoceras sorbifolia Bunge. *Eur J Med Chem*, **60**, 263-270.
1385. Xiao, W.L., Li, X.L., Wang, R.R. *et al.* (2007) Triterpenoids from Schisandra rubriflora. *J Nat Prod*, **70**, 1056-1059.
1386. Xiao, W.L., Yang, S.Y., Yang, L.M. *et al.* (2010) Chemical constituents from the leaves and stems of Schisandra rubriflora. *J Nat Prod*, **73**, 221-225.
1387. Xie, B.B., Hou, L., Guo, B.L. *et al.* (2014) [The compounds from n-butanol fraction of Alpinia oxyphylla]. *Yao Xue Xue Bao*, **49**, 1569-1573.
1388. Xie, F.G., Li, C.J., Yang, J.Z. *et al.* (2012) [Study on chemical constituents from the root bark of Tripterygium hypoglaucum]. *Zhong Yao Cai*, **35**, 1083-1087.
1389. Xin-Jia, Y., Wei, L., Ying, Z. *et al.* (2016) A New Biphenyl Neolignan from Leaves of Patrinia villosa (Thunb.) Juss. *Pharmacogn Mag*, **12**, 1-3.
1390. Xu, F.Q., Xu, F.C., Hou, B. *et al.* (2014) Cytotoxic bibenzyl dimers from the stems of Dendrobium fimbriatum Hook. *Bioorg Med Chem Lett*, **24**, 5268-5273.
1391. Xu, G., Kan, W.L., Zhou, Y. *et al.* (2010) Cytotoxic acylphloroglucinol derivatives from the twigs of Garcinia cowa. *J Nat Prod*, **73**, 104-108.
1392. Xu, H.X., Zeng, F.Q., Wan, M. *et al.* (1996) Anti-HIV triterpene acids from Geum japonicum. *J Nat Prod*, **59**, 643-645.

1393. Xu, J., Ji, C., Zhang, Y. *et al.* (2012) Inhibitory activity of eudesmane sesquiterpenes from Alpinia oxyphylla on production of nitric oxide. *Bioorg Med Chem Lett*, **22**, 1660-1663.
1394. Xu, J., Sun, Y., Wang, M. *et al.* (2015) Bioactive Diterpenoids from the Leaves of Callicarpa macrophylla. *J Nat Prod*, **78**, 1563-1569.
1395. Xu, M., Cui, J., Fu, H. *et al.* (2005) Embelin derivatives and their anticancer activity through microtubule disassembly. *Planta Med*, **71**, 944-948.
1396. Xu, M., Deng, Z., Li, M. *et al.* (2004) Chemical constituents from the mangrove plant, Aegiceras corniculatum. *J Nat Prod*, **67**, 762-766.
1397. Xu, M., Wang, D., Zhang, Y.J. *et al.* (2007) Dammarane triterpenoids from the roots of Gentiana rigescens. *J Nat Prod*, **70**, 880-883.
1398. Xu, M.Y. and Kim, Y.S. (2014) Antitumor activity of glycyrol via induction of cell cycle arrest, apoptosis and defective autophagy. *Food Chem Toxicol*, **74**, 311-319.
1399. Xu, S., Li, N., Ning, M.M. *et al.* (2006) Bioactive compounds from Peperomia pellucida. *J Nat Prod*, **69**, 247-250.
1400. Xu, W., Zhu, C., Cheng, W. *et al.* (2009) Chemical Constituents of the Roots of Euphorbia micractina. *J Nat Prod*, **72**, 1620-1626.
1401. Xu, Y.J., Wu, X.H., Tan, B.K. *et al.* (2000) Flavonol-cinnamate cycloadducts and diamide derivatives from Aglaia laxiflora. *J Nat Prod*, **63**, 473-476.
1402. Xu, Y.K., Yang, L., Liao, S.G. *et al.* (2015) Koumine, Humantenine, and Yohimbane Alkaloids from Gelsemium elegans. *J Nat Prod*, **78**, 1511-1517.
1403. Xu, Y.K., Yang, S.P., Liao, S.G. *et al.* (2006) Alkaloids from Gelsemium elegans. *J Nat Prod*, **69**, 1347-1350.
1404. Xu, Z., Chang, F.R., Wang, H.K. *et al.* (2000) Anti-HIV agents 45(1) and antitumor agents 205.(2) two new sesquiterpenes, leitneridanins A and B, and the cytotoxic and anti-HIV principles from Leitneria floridana. *J Nat Prod*, **63**, 1712-1715.
1405. Xue, P., Zhao, Y., Wang, B. *et al.* (2007) Simultaneous determination of seven flavonoids in Potentilla multifida by HPLC. *J Chromatogr Sci*, **45**, 216-219.
1406. Xue, Z., Li, S., Wang, S. *et al.* (2006) Mono-, Bi-, and triphenanthrenes from the tubers of Cremastra appendiculata. *J Nat Prod*, **69**, 907-913.
1407. Yamashita, M., Kaneko, M., Iida, A. *et al.* (2007) Stereoselective synthesis and cytotoxicity of a cancer chemopreventive naphthoquinone from Tabebuia avellanedae. *Bioorg Med Chem Lett*, **17**, 6417-6420.
1408. Yamashita, M., Kaneko, M., Tokuda, H. *et al.* (2009) Synthesis and evaluation of bioactive naphthoquinones from the Brazilian medicinal plant, Tabebuia avellanedae. *Bioorg Med Chem*, **17**, 6286-6291.
1409. Yan, C., Huang, L., Liu, H.C. *et al.* (2014) Spiramine derivatives induce apoptosis of Bax(-/-)/Bak(-/-) cell and cancer cells. *Bioorg Med Chem Lett*, **24**, 1884-1888.
1410. Yan, H., Ma, Z., Peng, S. *et al.* (2013) Anti-inflammatory effect of auraptene extracted from trifoliate orange (Poncirus trifoliate) on LPS-stimulated RAW 264.7 cells. *Inflammation*, **36**, 1525-1532.
1411. Yan, M., Zhu, Y., Zhang, H.J. *et al.* (2013) Anti-inflammatory secondary metabolites from the leaves of Rosa laevigata. *Bioorg Med Chem*, **21**, 3290-3297.
1412. Yan, X.H., Chen, J., Di, Y.T. *et al.* (2010) Anti-tobacco mosaic virus (TMV) Quassinoids from Brucea javanica (L.) Merr. *J Agric Food Chem*, **58**, 1572-1577.
1413. Yan, X.T., Li, W., Sun, Y.N. *et al.* (2014) Identification and biological evaluation of flavonoids from the fruits of Prunus mume. *Bioorg Med Chem Lett*, **24**, 1397-1402.
1414. Yan, Y.X., Hu, X.D., Chen, J.C. *et al.* (2009) Cytotoxic triterpenoid alkaloids from Buxus microphylla. *J Nat Prod*, **72**, 308-311.
1415. Yanfang, Z., Xingping, L., Zongde, Z. *et al.* (2006) Simultaneous determination of andrographolide and dehydroandrographolide in Andrographis paniculata and Chinese medicinal preparations by microemulsion electrokinetic chromatography. *J Pharm Biomed Anal*, **40**, 157-161.
1416. Yang, C., Yuan, C. and Jia, Z. (2003) Xanthanolides, germacranolides, and other constituents from Carpesium longifolium. *J Nat Prod*, **66**, 1554-1557.
1417. Yang, C.H., Cheng, M.J., Chiang, M.Y. *et al.* (2008) Dihydrobenzo[c]phenanthridine alkaloids from stem bark of Zanthoxylum nitidum. *J Nat Prod*, **71**, 669-673.
1418. Yang, G., Feng, H. and Li, Y. (2001) Di- and triterpenoids from Tripterygium wilfordii. *Nat Prod Lett*, **15**, 103-110.
1419. Yang, H., Jeong, E.J., Kim, J. *et al.* (2011) Antiproliferative triterpenes from the leaves and twigs of Juglans sinensis on HSC-T6 cells. *J Nat Prod*, **74**, 751-756.
1420. Yang, H., Sung, S.H. and Kim, Y.C. (2007) Antifibrotic phenanthrenes of Dendrobium nobile stems. *J Nat Prod*, **70**, 1925-1929.

1421. Yang, J.H., Kondratyuk, T.P., Jermihov, K.C. *et al.* (2011) Bioactive compounds from the fern Lepisorus contortus. *J Nat Prod*, **74**, 129-136.
1422. Yang, J.L., Ha, T.K., Dhodary, B. *et al.* (2014) Dammarane triterpenes as potential SIRT1 activators from the leaves of Panax ginseng. *J Nat Prod*, **77**, 1615-1623.
1423. Yang, M.C., Choi, S.U., Choi, W.S. *et al.* (2008) Guaiane sesquiterpene lactones and amino acid-sesquiterpene lactone conjugates from the aerial parts of Saussurea pulchella. *J Nat Prod*, **71**, 678-683.
1424. Yang, M.C., Kwon, H.C., Kim, Y.J. *et al.* (2010) Oploxynes A and B, polyacetylenes from the stems of Oplopanax elatus. *J Nat Prod*, **73**, 801-805.
1425. Yang, P., Liu, D.Q., Liang, T.J. *et al.* (2015) Bioactive constituents from the green alga Caulerpa racemosa. *Bioorg Med Chem*, **23**, 38-45.
1426. Yang, S.S., Cragg, G.M., Newman, D.J. *et al.* (2001) Natural product-based anti-HIV drug discovery and development facilitated by the NCI developmental therapeutics program. *J Nat Prod*, **64**, 265-277.
1427. Yang, S.W., Zhou, B.N., Wisse, J.H. *et al.* (1998) Three new ellagic acid derivatives from the bark of Eschweilera coriacea from the Suriname rainforest. *J Nat Prod*, **61**, 901-906.
1428. Yang, X., Wong, M., Wang, N. *et al.* (2006) A new eudesmane derivative and a new fatty acid ester from Sambucus williamsii. *Chem Pharm Bull (Tokyo)*, **54**, 676-678.
1429. Yang, X.W., Guo, Q.M. and Wang, Y. (2008) [Absorption and transport of 6 coumarins isolated from the roots of Angelica pubescens f. biserrata in human Caco-2 cell monolayer model]. *Zhong Xi Yi Jie He Xue Bao*, **6**, 392-398.
1430. Yang, X.W., Li, M.M., Liu, X. *et al.* (2015) Polycyclic Polyprenylated Acylphloroglucinol Congeners Possessing Diverse Structures from Hypericum henryi. *J Nat Prod*, **78**, 885-895.
1431. Yang, X.W. and Yan, Z.K. (1993) [Studies on the chemical constituents of alkaloids in seeds of Strychnos nux-vomica L]. *Zhongguo Zhong Yao Za Zhi*, **18**, 739-740, 763-734.
1432. Yang, X.W., Zhao, J., Cui, Y.X. *et al.* (1999) Anti-HIV-1 protease triterpenoid saponins from the seeds of Aesculus chinensis. *J Nat Prod*, **62**, 1510-1513.
1433. Yang, Y., Jiang, J., Qimei, L. *et al.* (2010) The fungicidal terpenoids and essential oil from Litsea cubeba in Tibet. *Molecules*, **15**, 7075-7082.
1434. Yang, Y., Yan, Y.M., Wei, W. *et al.* (2013) Anthraquinone derivatives from Rumex plants and endophytic Aspergillus fumigatus and their effects on diabetic nephropathy. *Bioorg Med Chem Lett*, **23**, 3905-3909.
1435. Yao-Kouassi, P.A., Magid, A.A., Richard, B. *et al.* (2008) Isoflavonoid glycosides from the roots of Baphia bancoensis. *J Nat Prod*, **71**, 2073-2076.
1436. Yap, W.S., Gan, C.Y., Low, Y.Y. *et al.* (2011) Grandilodines A-C, biologically active indole alkaloids from Kopsia grandifolia. *J Nat Prod*, **74**, 1309-1312.
1437. Yasuda, K., Kizu, H., Yamashita, T. *et al.* (2002) New sugar-mimic alkaloids from the pods of Angylocalyx pynaertii. *J Nat Prod*, **65**, 198-202.
1438. Ybarra, M.I., Popich, S., Borkosky, S.A. *et al.* (2005) Manoyl oxide diterpenoids from Grindelia scorzonerifolia. *J Nat Prod*, **68**, 554-558.
1439. Ye, M., Xiong, J., Zhu, J.J. *et al.* (2014) Leonurusoleanolides E-J, minor spirocyclic triterpenoids from Leonurus japonicus fruits. *J Nat Prod*, **77**, 178-182.
1440. Ye, X., Yu, S., Liang, Y. *et al.* (2014) Bioactive triterpenoid saponins and phenolic compounds against glioma cells. *Bioorg Med Chem Lett*, **24**, 5157-5163.
1441. Yen, C.T., Lee, C.L., Chang, F.R. *et al.* (2012) Indiosides G-K: steroidal glycosides with cytotoxic and anti-inflammatory activities from Solanum violaceum. *J Nat Prod*, **75**, 636-643.
1442. Yenesew, A., Irungu, B., Derese, S. *et al.* (2003) Two prenylated flavonoids from the stem bark of Erythrina burttii. *Phytochemistry*, **63**, 445-448.
1443. Yeom, G.G., Min, S. and Kim, S.Y. (2014) 2,3,5,6-Tetramethylpyrazine of Ephedra sinica regulates melanogenesis and inflammation in a UVA-induced melanoma/keratinocytes co-culture system. *Int Immunopharmacol*, **18**, 262-269.
1444. Yeon, E.T., Lee, J.W., Lee, C. *et al.* (2015) neo-Clerodane Diterpenoids from Scutellaria barbata and Their Inhibitory Effects on LPS-Induced Nitric Oxide Production. *J Nat Prod*, **78**, 2292-2296.
1445. Yin, H., Luo, J.G. and Kong, L.Y. (2013) Tetracyclic diterpenoids with isomerized isospongian skeleton and labdane diterpenoids from the fruits of Amomum kravanh. *J Nat Prod*, **76**, 237-242.
1446. Yin, S., Wang, X.N., Fan, C.Q. *et al.* (2007) Limonoids from the seeds of the marine mangrove Xylocarpus granatum. *J Nat Prod*, **70**, 682-685.
1447. Yin, W., Deng, X.K., Yin, F.Z. *et al.* (2007) The cytotoxicity induced by brucine from the seed of Strychnos nux-vomica proceeds via apoptosis and is mediated by cyclooxygenase 2 and caspase 3 in SMMC 7221 cells. *Food Chem Toxicol*, **45**, 1700-1708.
1448. Yokosuka, A., Jitsuno, M., Yui, S. *et al.* (2009) Steroidal glycosides from Agave utahensis and their cytotoxic activity. *J Nat Prod*, **72**, 1399-1404.

1449. Yokosuka, A., Mimaki, Y., Sakagami, H. *et al.* (2002) New diarylheptanoids and diarylheptanoid glucosides from the rhizomes of Tacca chantrieri and their cytotoxic activity. *J Nat Prod*, **65**, 283-289.
1450. Yokosuka, A., Mimaki, Y. and Sashida, Y. (2002) Steroidal and pregnane glycosides from the rhizomes of Tacca chantrieri. *J Nat Prod*, **65**, 1293-1298.
1451. Yokosuka, A., Sato, K., Yamori, T. *et al.* (2010) Triterpene glycosides from Curculigo orchioides and their cytotoxic activity. *J Nat Prod*, **73**, 1102-1106.
1452. Yonezawa, T., Lee, J.W., Akazawa, H. *et al.* (2011) Osteogenic activity of diphenyl ether-type cyclic diarylheptanoids derived from Acer nikoense. *Bioorg Med Chem Lett*, **21**, 3248-3251.
1453. Yoo, N.H., Jang, D.S., Yoo, J.L. *et al.* (2008) Erigeroflavanone, a flavanone derivative from the flowers of Erigeron annuus with protein glycation and aldose reductase inhibitory activity. *J Nat Prod*, **71**, 713-715.
1454. Yoon, J.S., Lee, M.K., Sung, S.H. *et al.* (2006) Neuroprotective 2-(2-phenylethyl)chromones of Imperata cylindrica. *J Nat Prod*, **69**, 290-291.
1455. Yoon, J.S., Sung, S.H. and Kim, Y.C. (2008) Neuroprotective limonoids of root bark of Dictamnus dasycarpus. *J Nat Prod*, **71**, 208-211.
1456. Yoon, K.D., Jeong, D.G., Hwang, Y.H. *et al.* (2007) Inhibitors of osteoclast differentiation from Cephalotaxus koreana. *J Nat Prod*, **70**, 2029-2032.
1457. Yoshikawa, K., Tanaka, M., Arihara, S. *et al.* (2000) New oleanene triterpenoid saponins from Madhuca longifolia. *J Nat Prod*, **63**, 1679-1681.
1458. Yoshikawa, M., Murakami, T., Ishiwada, T. *et al.* (2002) New flavonol oligoglycosides and polyacylated sucroses with inhibitory effects on aldose reductase and platelet aggregation from the flowers of Prunus mume. *J Nat Prod*, **65**, 1151-1155.
1459. Yoshikawa, M., Xu, F., Morikawa, T. *et al.* (2003) Anastatins A and B, new skeletal flavonoids with hepatoprotective activities from the desert plant Anastatica hierochuntica. *Bioorg Med Chem Lett*, **13**, 1045-1049.
1460. Yoshimura, M., Yamakami, S., Amakura, Y. *et al.* (2012) Diarylheptanoid sulfates and related compounds from Myrica rubra bark. *J Nat Prod*, **75**, 1798-1802.
1461. Youkwan, J., Sutthivaiyakit, S. and Sutthivaiyakit, P. (2010) Citrusosides A-D and furanocoumarins with cholinesterase inhibitory activity from the fruit peels of Citrus hystrix. *J Nat Prod*, **73**, 1879-1883.
1462. Youn, U.J., Chen, Q.C., Jin, W.Y. *et al.* (2007) Cytotoxic lignans from the stem bark of Magnolia officinalis. *J Nat Prod*, **70**, 1687-1689.
1463. Youn, U.J., Lee, Y.S., Jeong, H. *et al.* (2009) Identification of antiadipogenic constituents of the rhizomes of Anemarrhena asphodeloides. *J Nat Prod*, **72**, 1895-1898.
1464. Youn, U.J., Park, E.J., Kondratyuk, T.P. *et al.* (2012) Anti-inflammatory sesquiterpene lactones from the flower of Vernonia cinerea. *Bioorg Med Chem Lett*, **22**, 5559-5562.
1465. Yrjonen, T., Eeva, M., Kauppila, T.J. *et al.* (2016) Profiling of Coumarins in Peucedanum palustre (L.) Moench Populations Growing in Finland. *Chem Biodivers*, **13**, 700-709.
1466. Yu, B.W., Meng, L.H., Chen, J.Y. *et al.* (2001) Cytotoxic oxoisoaporphine alkaloids from Menispermum dauricum. *J Nat Prod*, **64**, 968-970.
1467. Yu, F., Wang, Q., Wei, S. *et al.* (2015) Effect of genotype and environment on five bioactive components of cultivated licorice (Glycyrrhiza uralensis) populations in northern China. *Biol Pharm Bull*, **38**, 75-81.
1468. Yu, H.Y., Chen, Z.Y., Sun, B. *et al.* (2014) Lignans from the fruit of Schisandra glaucescens with antioxidant and neuroprotective properties. *J Nat Prod*, **77**, 1311-1320.
1469. Yu, L., Wang, X., Wei, X. *et al.* (2012) Triterpenoid saponins from Xanthoceras sorbifolia Bunge and their inhibitory activity on human cancer cell lines. *Bioorg Med Chem Lett*, **22**, 5232-5238.
1470. Yu, L.L., Hu, W.C., Ding, G. *et al.* (2011) Gusanlungionosides A-D, potential tyrosinase inhibitors from Arcangelisia gusanlung. *J Nat Prod*, **74**, 1009-1014.
1471. Yu, Y., Song, W., Zhu, C. *et al.* (2011) Homosecoiridoids from the flower buds of Lonicera japonica. *J Nat Prod*, **74**, 2151-2160.
1472. Yu, Y., Xie, Z.L., Gao, H. *et al.* (2009) Bioactive iridoid glucosides from the fruit of Gardenia jasminoides. *J Nat Prod*, **72**, 1459-1464.
1473. Yu, Y.M., Yang, J.S., Peng, C.Z. *et al.* (2009) Lactones from Angiopteris caudatiformis. *J Nat Prod*, **72**, 921-924.
1474. Yu, Y.U., Kang, S.Y., Park, H.Y. *et al.* (2000) Antioxidant lignans from Machilus thunbergii protect CCl4-injured primary cultures of rat hepatocytes. *J Pharm Pharmacol*, **52**, 1163-1169.
1475. Yuan, C.M., Zhang, Y., Tang, G.H. *et al.* (2013) Khayseneganins A-H, limonoids from Khaya senegalensis. *J Nat Prod*, **76**, 327-333.
1476. Yuan, D., Ma, B., Wu, C. *et al.* (2008) Alkaloids from the leaves of Uncaria rhynchophylla and their inhibitory activity on NO production in lipopolysaccharide-activated microglia. *J Nat Prod*, **71**, 1271-1274.

1477. Yuan, Q.Y. and Liu, X.Q. (2012) [Chemical constituents from Pleione bulbocodioides]. *Zhong Yao Cai*, **35**, 1602-1604.
1478. Yuan, T., Wan, C., Gonzalez-Sarrias, A. *et al.* (2011) Phenolic glycosides from sugar maple (Acer saccharum) bark. *J Nat Prod*, **74**, 2472-2476.
1479. Yun, B.S., Ryoo, I.J., Lee, I.K. *et al.* (1999) Two bioactive pentacyclic triterpene esters from the root bark of Hibiscus syriacus. *J Nat Prod*, **62**, 764-766.
1480. Zaheer-Ul-Haq, Z.U., Wellenzohn, B., Liedl, K.R. *et al.* (2003) Molecular docking studies of natural cholinesterase-inhibiting steroidal alkaloids from Sarcococca saligna. *J Med Chem*, **46**, 5087-5090.
1481. Zaheer ul, H., Wellenzohn, B., Tonmunphean, S. *et al.* (2003) 3D-QSAR studies on natural acetylcholinesterase inhibitors of Sarcococca saligna by comparative molecular field analysis (CoMFA). *Bioorg Med Chem Lett*, **13**, 4375-4380.
1482. Zaugg, J., Eickmeier, E., Ebrahimi, S.N. *et al.* (2011) Positive GABA(A) receptor modulators from Acorus calamus and structural analysis of (+)-dioxosarcoguaiacol by 1D and 2D NMR and molecular modeling. *J Nat Prod*, **74**, 1437-1443.
1483. Zelefack, F., Guilet, D., Fabre, N. *et al.* (2009) Cytotoxic and antiplasmodial xanthones from Pentadesma butyracea. *J Nat Prod*, **72**, 954-957.
1484. Zelova, H., Hanakova, Z., Cermakova, Z. *et al.* (2014) Evaluation of anti-inflammatory activity of prenylated substances isolated from Morus alba and Morus nigra. *J Nat Prod*, **77**, 1297-1303.
1485. Zeng, L., Wu, F.E., Oberlies, N.H. *et al.* (1996) Five new monotetrahydrofuran ring acetogenins from the leaves of Annona muricata. *J Nat Prod*, **59**, 1035-1042.
1486. Zeng, N., Shen, Y., Li, L.Z. *et al.* (2011) Anti-inflammatory triterpenes from the leaves of Rosa laevigata. *J Nat Prod*, **74**, 732-738.
1487. Zhang, C.P. (1989) [Studies on triterpenoids of total glucosides of Tripterygium wilfordii (T II)]. *Zhongguo Yi Xue Ke Xue Yuan Xue Bao*, **11**, 322-325.
1488. Zhang, C.P., Zhang, Y.G., Zheng, Q.T. *et al.* (1989) [The isolation and structure identification of triptotriterpenic acid C]. *Yao Xue Xue Bao*, **24**, 225-228.
1489. Zhang, C.R., Liu, H.B., Feng, T. *et al.* (2009) Alkaloids from the leaves of Daphniphyllum subverticillatum. *J Nat Prod*, **72**, 1669-1672.
1490. Zhang, F., Wang, J.S., Gu, Y.C. *et al.* (2012) Cytotoxic and anti-inflammatory triterpenoids from Toona ciliata. *J Nat Prod*, **75**, 538-546.
1491. Zhang, G., Shimokawa, S., Mochizuki, M. *et al.* (2008) Chemical constituents of Aristolochia constricta: antispasmodic effects of its constituents in guinea-pig ileum and isolation of a diterpeno-lignan hybrid. *J Nat Prod*, **71**, 1167-1172.
1492. Zhang, G.L., Li, N., Wang, Y.H. *et al.* (2007) Bioactive lignans from Peperomia heyneana. *J Nat Prod*, **70**, 662-664.
1493. Zhang, G.P., Xiao, Z.Y., Rafique, J. *et al.* (2009) Antiplasmodial isoflavanones from the roots of Sophora mollis. *J Nat Prod*, **72**, 1265-1268.
1494. Zhang, H., Bazzill, J., Gallagher, R.J. *et al.* (2013) Antiproliferative withanolides from Datura wrightii. *J Nat Prod*, **76**, 445-449.
1495. Zhang, H., Samadi, A.K., Gallagher, R.J. *et al.* (2011) Cytotoxic withanolide constituents of Physalis longifolia. *J Nat Prod*, **74**, 2532-2544.
1496. Zhang, H., Samadi, A.K., Rao, K.V. *et al.* (2011) Cytotoxic oleanane-type saponins from Albizia inundata. *J Nat Prod*, **74**, 477-482.
1497. Zhang, H., Tao, L., Fu, W.W. *et al.* (2014) Prenylated benzoylphloroglucinols and xanthones from the leaves of Garcinia oblongifolia with antienteroviral activity. *J Nat Prod*, **77**, 1037-1046.
1498. Zhang, H., Wu, Q., Li, W. *et al.* (2014) Absorption and metabolism of three monoester-diterpenoid alkaloids in Aconitum carmichaeli after oral administration to rats by HPLC-MS. *J Ethnopharmacol*, **154**, 645-652.
1499. Zhang, H., Yang, F., Qi, J. *et al.* (2010) Homoisoflavonoids from the fibrous roots of Polygonatum odoratum with glucose uptake-stimulatory activity in 3T3-L1 adipocytes. *J Nat Prod*, **73**, 548-552.
1500. Zhang, H., Zhang, D.D., Lao, Y.Z. *et al.* (2014) Cytotoxic and anti-inflammatory prenylated benzoylphloroglucinols and xanthones from the twigs of Garcinia esculenta. *J Nat Prod*, **77**, 1700-1707.
1501. Zhang, H.F., Yan, L.H., Zhang, Q.W. *et al.* (2013) [Flavonoids from leaves of Epimedium pubescens]. *Zhongguo Zhong Yao Za Zhi*, **38**, 1942-1946.
1502. Zhang, H.J., Ma, C., Nguyen, V.H. *et al.* (2006) Miliusanes, a class of cytotoxic agents from Miliusa sinensis. *J Med Chem*, **49**, 693-708.
1503. Zhang, H.J., Tan, G.T., Hoang, V.D. *et al.* (2003) Natural anti-HIV agents. Part IV. Anti-HIV constituents from Vatica cinerea. *J Nat Prod*, **66**, 263-268.
1504. Zhang, H.J., Tan, G.T., Santarsiero, B.D. *et al.* (2003) New Sesquiterpenes from Litsea verticillata. *J Nat Prod*, **66**, 609-615.

1505. Zhang, J., Wang, S., Li, Y. *et al.* (2013) Anti-diarrheal constituents of Alpinia oxyphylla. *Fitoterapia*, **89**, 149-156.
1506. Zhang, J., Yao, E., Wang, J. *et al.* (2007) [Extraction and identification of volatile constituents in the flowers of Aglaia odorata Lour]. *Se Pu*, **25**, 422-424.
1507. Zhang, J.S., Wang, X.M., Dong, X.H. *et al.* (2009) [Studies on chemical constituents of Swertia mussotii]. *Zhong Yao Cai*, **32**, 511-514.
1508. Zhang, J.X., Guan, S.H., Feng, R.H. *et al.* (2013) Neolignanamides, lignanamides, and other phenolic compounds from the root bark of Lycium chinense. *J Nat Prod*, **76**, 51-58.
1509. Zhang, L.B., Ji, J., Lei, C. *et al.* (2012) Isoprenylated flavonoid and adipogenesis-promoting constituents of Dodonaea viscosa. *J Nat Prod*, **75**, 699-706.
1510. Zhang, L.J., Chiou, C.T., Cheng, J.J. *et al.* (2010) Cytotoxic polyisoprenyl benzophenonoids from Garcinia subelliptica. *J Nat Prod*, **73**, 557-562.
1511. Zhang, M.L., Irwin, D., Li, X.N. *et al.* (2012) PPARgamma agonist from Chromolaena odorata. *J Nat Prod*, **75**, 2076-2081.
1512. Zhang, P.P., Gao, S.S., Zhang, T.T. *et al.* (2012) [Sesquiterpenes from stem of Schisandra glaucescens]. *Zhongguo Zhong Yao Za Zhi*, **37**, 3426-3429.
1513. Zhang, S., Zhao, M., Bai, L. *et al.* (2006) Bioactive guaianolides from siyekucai (Ixeris chinensis). *J Nat Prod*, **69**, 1425-1428.
1514. Zhang, S.Y., Zhang, Q.H., Zhao, W. *et al.* (2012) Isolation, characterization and cytotoxic activity of benzophenone glucopyranosides from Mahkota Dewa (Phaleria macrocarpa (Scheff.) Boerl). *Bioorg Med Chem Lett*, **22**, 6862-6866.
1515. Zhang, W., Huang, X.J., Zhang, S.Y. *et al.* (2015) Geleganidines A-C, Unusual Monoterpenoid Indole Alkaloids from Gelsemium elegans. *J Nat Prod*, **78**, 2036-2044.
1516. Zhang, W., Zhang, X.X., Liu, C.F. *et al.* (2013) [Study on chemical constituents of Kochia scoparia]. *Zhong Yao Cai*, **36**, 921-924.
1517. Zhang, W.D., Han, G.Y. and Liang, H.Q. (1992) [Studies on the alkaloid constituents of Jiangyou fu-zi Aconitum carmichaeli from Sichuan]. *Yao Xue Xue Bao*, **27**, 670-673.
1518. Zhang, X., Xu, J.K., Wang, J. *et al.* (2007) Bioactive bibenzyl derivatives and fluorenones from Dendrobium nobile. *J Nat Prod*, **70**, 24-28.
1519. Zhang, X.F., Wang, H.M., Song, Y.L. *et al.* (2006) Isolation, structure elucidation, antioxidative and immunomodulatory properties of two novel dihydrocoumarins from Aloe vera. *Bioorg Med Chem Lett*, **16**, 949-953.
1520. Zhang, X.L., Wang, L., Li, F. *et al.* (2013) Cytotoxic phorbol esters of Croton tiglium. *J Nat Prod*, **76**, 858-864.
1521. Zhang, Y., Liu, Y.B., Li, Y. *et al.* (2013) Sesquiterpenes and alkaloids from the roots of Alangium chinense. *J Nat Prod*, **76**, 1058-1063.
1522. Zhang, Y.B., Li, W. and Yang, X.W. (2012) Biotransformation of columbianadin by rat hepatic microsomes and inhibition of biotransformation products on NO production in RAW 264.7 cells in vitro. *Phytochemistry*, **81**, 109-116.
1523. Zhang, Y.M. and Zhang, P.Z. (2015) [Lignans from Stem Bark of Styrax perkinsiae]. *Zhong Yao Cai*, **38**, 1202-1205.
1524. Zhang, Y.N., Zhong, X.G., Zheng, Z.P. *et al.* (2007) Discovery and synthesis of new immunosuppressive alkaloids from the stem of Fissistigma oldhamii (Hemsl.) Merr. *Bioorg Med Chem*, **15**, 988-996.
1525. Zhang, Z., Wang, X., Yang, W. *et al.* (2016) Five 2-(2-Phenylethyl)chromones from Sodium Chloride-Elicited Aquilaria sinensis Cell Suspension Cultures. *Molecules*, **21**.
1526. Zhao, A.H., Zhang, Y.B. and Yang, X.W. (2016) Simultaneous determination and pharmacokinetics of sixteen Angelicae dahurica coumarins in vivo by LC-ESI-MS/MS following oral delivery in rats. *Phytomedicine*, **23**, 1029-1036.
1527. Zhao, C., Shao, J., Cao, D. *et al.* (2009) [Chemical constituents of Galium verum]. *Zhongguo Zhong Yao Za Zhi*, **34**, 2761-2764.
1528. Zhao, F., Watanabe, Y., Nozawa, H. *et al.* (2005) Prenylflavonoids and phloroglucinol derivatives from hops (Humulus lupulus). *J Nat Prod*, **68**, 43-49.
1529. Zhao, J.M., Li, N., Zhang, H. *et al.* (2011) Novel dammarane-type sapogenins from Panax ginseng berry and their biological activities. *Bioorg Med Chem Lett*, **21**, 1027-1031.
1530. Zhao, M., Bai, L., Wang, L. *et al.* (2007) Bioactive cardenolides from the stems and twigs of Nerium oleander. *J Nat Prod*, **70**, 1098-1103.
1531. Zhao, M., Zhang, S., Fu, L. *et al.* (2006) Taraxasterane- and ursane-type triterpenes from Nerium oleander and their biological activities. *J Nat Prod*, **69**, 1164-1167.

1532. Zhao, W., Huang, X.X., Yu, L.H. *et al.* (2014) Tomensides A-D, new antiproliferative phenylpropanoid sucrose esters from Prunus tomentosa leaves. *Bioorg Med Chem Lett*, **24**, 2459-2462.
1533. Zhao, W., Pu, J.X., Du, X. *et al.* (2011) Structure and cytotoxicity of diterpenoids from Isodon adenolomus. *J Nat Prod*, **74**, 1213-1220.
1534. Zhao, W., Zhou, T., Fan, G. *et al.* (2007) Isolation and purification of lignans from Magnolia biondii Pamp by isocratic reversed-phase two-dimensional liquid chromatography following microwave-assisted extraction. *J Sep Sci*, **30**, 2370-2381.
1535. Zhao, Y., Geng, C.A., Chen, H. *et al.* (2015) Isolation, synthesis and anti-hepatitis B virus evaluation of p-hydroxyacetophenone derivatives from Artemisia capillaris. *Bioorg Med Chem Lett*, **25**, 1509-1514.
1536. Zhao, Y., Huang, S.X., Yang, L.B. *et al.* (2009) Cytotoxic ent-kaurane diterpenoids from Isodon henryi. *Planta Med*, **75**, 65-69.
1537. Zhao, Y., Pu, J.X., Huang, S.X. *et al.* (2009) ent-Kaurane diterpenoids from Isodon pharicus. *J Nat Prod*, **72**, 988-993.
1538. Zhao, Y., Pu, J.X., Huang, S.X. *et al.* (2009) ent-Kaurane diterpenoids from Isodon scoparius. *J Nat Prod*, **72**, 125-129.
1539. Zhao, Y.M., Wang, J., Liu, H.B. *et al.* (2015) Microwave-assisted Extraction of Alantolactone and Isoalantolactone from Inula helenium. *Indian J Pharm Sci*, **77**, 116-120.
1540. Zheng, C.J., Huang, B.K., Han, T. *et al.* (2009) Nitric oxide scavenging lignans from Vitex negundo seeds. *J Nat Prod*, **72**, 1627-1630.
1541. Zheng, C.J., Huang, B.K., Wang, Y. *et al.* (2010) Anti-inflammatory diterpenes from the seeds of Vitex negundo. *Bioorg Med Chem*, **18**, 175-181.
1542. Zheng, C.J., Zhu, J.Y., Yu, W. *et al.* (2013) Labdane-type diterpenoids from the fruits of Vitex trifolia. *J Nat Prod*, **76**, 287-291.
1543. Zheng, Q.X., Xu, Z.J., Sun, X.F. *et al.* (2003) Eudesmane derivatives and other sesquiterpenes from Laggera alata. *J Nat Prod*, **66**, 1078-1081.
1544. Zheng, X.Q., Koyama, Y., Nagai, C. *et al.* (2004) Biosynthesis, accumulation and degradation of theobromine in developing Theobroma cacao fruits. *J Plant Physiol*, **161**, 363-369.
1545. Zhou, B.N., Bahler, B.D., Hofmann, G.A. *et al.* (1998) Phenylethanoid glycosides from Digitalis purpurea and Penstemon linarioides with PKCalpha-inhibitory activity. *J Nat Prod*, **61**, 1410-1412.
1546. Zhou, B.N., Baj, N.J., Glass, T.E. *et al.* (1997) Bioactive labdane diterpenoids from Renealmia alpinia collected in the Suriname rainforest. *J Nat Prod*, **60**, 1287-1293.
1547. Zhou, G.X., Chen, R.Y., Zhang, Y.J. *et al.* (2000) New annonaceous acetogenins from the roots of Uvaria calamistrata. *J Nat Prod*, **63**, 1201-1204.
1548. Zhou, G.X., Wijeratne, E.M., Bigelow, D. *et al.* (2004) Aspochalasins I, J, and K: three new cytotoxic cytochalasans of Aspergillus flavipes from the rhizosphere of Ericameria laricifolia of the Sonoran Desert. *J Nat Prod*, **67**, 328-332.
1549. Zhou, G.X., Zhou, L.E., Chen, R.Y. *et al.* (1999) Calamistrins A and B, two new cytotoxic monotetrahydrofuran annonaceous acetogenins from Uvaria calamistrata. *J Nat Prod*, **62**, 261-264.
1550. Zhou, H., Hamazaki, A., Fontana, J.D. *et al.* (2004) New ring C-seco limonoids from Brazilian Melia azedarach and their cytotoxic activity. *J Nat Prod*, **67**, 1544-1547.
1551. Zhou, J., Li, C.J., Yang, J.Z. *et al.* (2014) Lupane triterpenoids from the stems of Euonymus carnosus. *J Nat Prod*, **77**, 276-284.
1552. Zhou, K., Zhao, F., Liu, Z. *et al.* (2009) Triterpenoids and flavonoids from celery (Apium graveolens). *J Nat Prod*, **72**, 1563-1567.
1553. Zhou, X., He, X., Wang, G. *et al.* (2006) Steroidal saponins from Solanum nigrum. *J Nat Prod*, **69**, 1158-1163.
1554. Zhu, Y., Miao, Z., Ding, J. *et al.* (2008) Cytotoxic dihydroagarofuranoid sesquiterpenes from the seeds of Celastrus orbiculatus. *J Nat Prod*, **71**, 1005-1010.
1555. Zhu, Y., Pu, B.Q., Xie, G.Y. *et al.* (2014) Dynamic changes of flavonoids contents in the different parts of rhizome of Belamcanda chinensis during the thermal drying process. *Molecules*, **19**, 10440-10454.
1556. Zhu, Y., Zhang, P., Yu, H. *et al.* (2007) Anti-Helicobacter pylori and thrombin inhibitory components from Chinese dragon's blood, Dracaena cochinchinensis. *J Nat Prod*, **70**, 1570-1577.
1557. Zi, J., Li, S., Liu, M. *et al.* (2008) Glycosidic constituents of the tubers of Gymnadenia conopsea. *J Nat Prod*, **71**, 799-805.
1558. Zi, J. and Peters, R.J. (2013) Characterization of CYP76AH4 clarifies phenolic diterpenoid biosynthesis in the Lamiaceae. *Org Biomol Chem*, **11**, 7650-7652.
1559. Ziaei, A., Ramezani, M., Wright, L. *et al.* (2011) Identification of spathulenol in Salvia mirzayanii and the immunomodulatory effects. *Phytother Res*, **25**, 557-562.
1560. Ziegler, S.J. and Sticher, O. (1989) HPLC of S-Alk(en)yl-L-cysteine Derivatives in Garlic including Quantitative Determination of (+)-S-Allyl-L-cysteine Sulfoxide (Alliin). *Planta Med*, **55**, 372-378.

1561. Zou, K., Zhu, S., Meselhy, M.R. *et al.* (2002) Dammarane-type Saponins from Panax japonicus and their neurite outgrowth activity in SK-N-SH cells. *J Nat Prod*, **65**, 1288-1292.
1562. Zou, Z.M. and Cong, P.Z. (1991) [Studies on the chemical constituents from roots of Agastache rugosa]. *Yao Xue Xue Bao*, **26**, 906-910.
